# Highly reversible extrinsic electrocaloric effects over a wide temperature range in epitaxially strained SrTiO_3_ films

**DOI:** 10.1038/s41563-024-01831-1

**Published:** 2024-03-21

**Authors:** S. Zhang, J. Deliyore-Ramírez, S. Deng, B. Nair, D. Pesquera, Q. Jing, M. E. Vickers, S. Crossley, M. Ghidini, S. Kar-Narayan, G. G. Guzmán-Verri, X. Moya, N. D. Mathur

**Affiliations:** 1 College of Science, National University of Defense Technology, Changsha, China; 2 Department of Materials Science, University of Cambridge, Cambridge, UK; 3 Centro de Investigación en Ciencia e Ingeniería de Materiales (CICIMA), Universidad de Costa Rica, San José, Costa Rica; 4 Escuela de Física, Universidad de Costa Rica, San José, Costa Rica; 5 Beijing Advanced Innovation Center for Materials Genome Engineering, University of Science and Technology Beijing, Beijing, China; 6 James Watt School of Engineering, University of Glasgow, Glasgow, UK; 7 DiFeST, University of Parma, Parma, Italy; 8 Diamond Light Source, Chilton, Didcot, UK

**Keywords:** Ferroelectrics and multiferroics, Phase transitions and critical phenomena, Surfaces, interfaces and thin films

## Abstract

Electrocaloric effects have been experimentally studied in ferroelectrics and incipient ferroelectrics, but not incipient ferroelectrics driven ferroelectric using strain. Here we use optimally oriented interdigitated surface electrodes to investigate extrinsic electrocaloric effects in low-loss epitaxial SrTiO_3_ films near the broad second-order 243 K ferroelectric phase transition created by biaxial in-plane coherent tensile strain from DyScO_3_ substrates. Our extrinsic electrocaloric effects are an order of magnitude larger than the corresponding effects in bulk SrTiO_3_ over a wide range of temperatures including room temperature, and unlike electrocaloric effects associated with first-order transitions they are highly reversible in unipolar applied fields. Additionally, the canonical Landau description for strained SrTiO_3_ films works well if we set the low-temperature zero-field polarization along one of the in-plane pseudocubic <100> directions. In future, similar strain engineering could be exploited for other films, multilayers and bulk samples to increase the range of electrocaloric materials for energy efficient cooling.

## Abstract (web summary)

Electrocaloric effects are large in a limited set of materials that display hysteretic first-order phase transitions. Here epitaxial SrTiO_3_ thin films are strain engineered to achieve anhysteretic second-order phase transitions, with electrocaloric effects enhanced by one order of magnitude over bulk.

## Main

Electrocaloric (EC) effects are reversible or nominally reversible^1^ thermal changes that arise when changes of electric field drive changes in the magnitude of the local electrical polarization. While analogous magnetocaloric effects have been exploited for almost a century to achieve ultra-low temperatures in scientific laboratories^2^, the current push for environmentally friendly cooling and heating has led to a proliferation of prototypes based on EC, magnetocaloric and mechanocaloric materials near phase transitions close to room temperature^1,3–9^. Many of the EC prototypes^10–16^ are based on EC effects that arise in BaTiO_3_ and PbSc_0.5_Ta_0.5_O_3_ near ferroelectric transitions that can be modified via grain size^17^, doping^18^, strain^19^ and pressure^20^. Here we experimentally explore EC effects that arise in epitaxial films of SrTiO_3_ (STO) near a ferroelectric transition that is created, rather than modified, by strain. Unlike the widely studied EC effects associated with hysteretic first-order transitions^1^, our extrinsic EC effects are highly reversible both above and below the transition temperature, partly because the strain-induced transition is second order, and partly because we minimize the irreversible formation of ferroelectric domains below the Curie temperature by applying and removing unipolar fields.

Bulk STO is a well-known dielectric whose rich physics^21^ includes an incipient ferroelectric transition^22^ that enhances EC effects at cryogenic temperatures^23^. The ferroelectric transition can be mechanically induced in single crystals at 4.2 K (ref. ^24^) and at much higher temperatures in epitaxially grown films^25,26^ of the type we explore here. Specifically, we study pseudocubic (pc) films of STO (001)_pc_ on orthorhombic (o) substrates of DSO (110)_o_ (DSO denotes DyScO_3_; all STO films described in this paper are (001)_pc_ oriented). If these STO//DSO films display some relaxation (principal in-plane strains just below 1%) then they possess a low-temperature zero-field polarization along one of the in-plane <110>_pc_ directions^25,27^. By contrast, our coherently strained STO films with negligible relaxation (principal in-plane strains of ~1%) possess a low-temperature zero-field polarization along the longer of the two in-plane <100>_pc_ lattice vectors^28^.

Detailed electrical measurements along this direction were performed via silver interdigitated electrodes (IDEs) in order to map the dielectric response *ε*_film_ and the electrical polarization *P*_film_ of the STO film while varying the temperature *T* and electric field *E* (here and elsewhere, we use dielectric response to describe real parts of relative permittivities). Both *ε*_film_ and *P*_film_ were quantified after performing equivalent measurements of a DSO substrate with equivalent IDEs, and conformally mapping the repeating film–substrate–air units in the IDE geometry to the parallel-plate geometry, as shown for permittivity but not polarization in a PhD thesis^29^ and references therein. Conformal mapping assumes isotropic and homogeneous media. Our addressed film can be considered homogeneous because it comprises a single domain above the Curie temperature *T*_C_ as well as at most (some) fields and temperatures during unipolar (bipolar) cycles below *T*_C_. Any ferroelectric domains run between adjacent electrodes^26^ and can thus be lumped together, conformally mapped and additively combined. Our addressed film is effectively isotropic because field and polarization are electrostatically confined to the film plane by dissimilar dielectric responses of the film, substrate and air^29,30^. The dielectric response of our DSO substrate is highly isotropic^25^, and field independence implies homogeneity at temperatures of interest, despite the IDE geometry. Similarly, the dielectric response of bulk STO is isotropic, and approximate field independence above 100 K (ref. ^31^) implies approximate homogeneity at temperatures of interest, despite the IDE geometry.

As expected, dielectric measurements show evidence of a second-order phase transition, a Curie temperature of *T*_C_ ≈ 243 K (refs. ^25,32,33^) and weak relaxor behaviour^30,34^. However, our loss tangent of 2–4% is an order of magnitude smaller than previously reported values for both relaxed^25,30,34^ and coherently strained^28^ films. Highly reversible extrinsic EC effects are identified via thermodynamic analysis of isothermal electrical polarization data that we obtain both directly and, for interest, by integrating dielectric data. Using our maximum field of |*E*| = 20 kV cm^–1^, the peak isothermal entropy change in the film is |Δ*S*_film_| ≈ 1.8 kJ K^–1^ m^–3^ at 242 K, exceeding the corresponding EC effects in bulk STO by one order of magnitude over a wide temperature range. (Numerical data reported here and in the Supplementary Information are based on unipolar data at *E* ≥ 0.)

Our EC effects match well with predictions based on the canonical Landau model for STO films under equibiaxial in-plane strain^32,33^ (our ~1% principal in-plane strains differ by just 0.02%). Previous such predictions^35,36^ assume unrealistic breakdown fields and implicitly assume an inhomogeneous polarization given that structural relaxation is required to set the low-temperature zero-field polarization along one of the in-plane <110>_pc_ directions^25,27^ (the canonical Landau model predicts <110>_pc_ following a revision^33^ of the initial <100>_pc_ prediction^32^). Here we use the canonical Landau model^32,33^ to describe with good fidelity the behaviour (dielectric properties, electrical polarization, EC effects) of our coherently strained films after modifying one parameter to set the low-temperature zero-field polarization along an in-plane <100>_pc_ direction^27^, and after modifying two other parameters to set polarization magnitude and Curie temperature. The Landau assumption of homogeneous polarization is good as we conformally map a single domain in coherently strained films to the parallel-plate geometry.

The experimental study of EC effects in epitaxial films is rare^37–39^, as is the study^40,41^ of EC effects associated with second-order transitions, where the electric field suppresses polarization fluctuations^42^, unlike first-order transitions, where the driven transition yields latent heat. Our strain-induced transition expands the limited set of existing EC materials and should inspire further studies in various geometries.

## Epitaxially strained STO

**Fig. 1:**
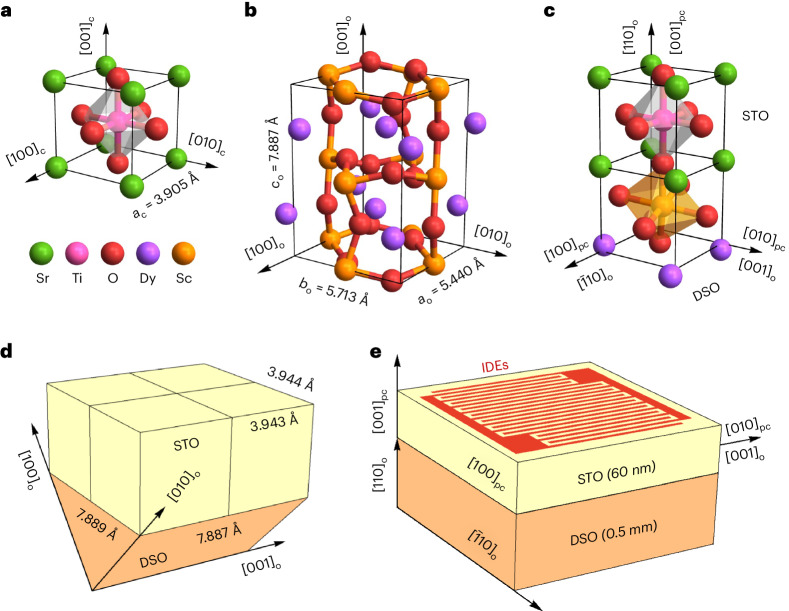
Strained STO (001)_pc_ films on DSO (110)_o_ substrates. **a**, The cubic unit cell of STO^43^. **b**, The orthorhombic *Pbnm* unit cell of DSO^25^. **c**, One pseudocubic unit cell of the strained STO film on one pseudocubic unit cell of the DSO substrate (the top half of the rotated ScO_6_ octahedron^29^ is distorted for structural continuity). **d**, Four pseudocubic unit cells of interfacial STO on half a DSO unit cell (orange triangle is scalene). The STO in-plane lattice parameters are epitaxially expanded by ~1% to *a*_pc_ ≈ 3.943 Å and *b*_pc_ ≈ 3.944 Å. **e**, Not-to-scale schematic of the ~60-nm-thick STO film and the 0.5-mm-thick DSO substrate. Interdigitated silver electrodes permit electric fields to be applied across 50 μm gaps along the STO [100]_pc_ direction parallel to DSO $\left[\bar{1}10\right]_{\mathrm{o}}$.

The cubic unit cell of bulk STO (*a*_c_ = 3.905 Å; Fig. 1a)^43^ and the orthorhombic *Pbnm* unit cell of DSO (*a*_o_ = 5.440 Å, *b*_o_ = 5.713 Å and *c*_o_ = 7.887 Å; Fig. 1b)^25^ permit cube-on-cube epitaxy (Fig. 1c) because DSO (110)_o_ presents an approximately square $(\sqrt{a_{\mathrm{o}}^2+b_{\mathrm{o}}^2}=7.889{\mathring{\mathrm{A}}})\times(c_{\mathrm{o}}=7.887{\mathring{\mathrm{A}}})$ template that is well matched to four STO (001)_pc_ unit cells, whose in-plane lattice parameters are expanded to *a*_pc_ ≈ 3.944 Å and *b*_pc_ ≈ 3.943 Å (Fig. 1d), such that principal in-plane strains of ~1% differ by just 0.02%. Our ~60-nm-thick STO (001)_pc_ films were grown on DSO (110)_o_ substrates at high temperature using pulsed laser deposition (Methods). Cooling to room temperature did not introduce substantial differential strain as the two materials possess similar thermal expansivities^44^.

Silver IDEs (thickness, 2 µm; width, 25 µm; gap *g* = 50 µm) were deposited by aerosol jet printing (Methods) for electrical measurements along the [100]_pc_ direction parallel to ${[\overline{1}10]}_{\mathrm{{o}}}$ in DSO (Fig. 1e; Supplementary Note 1 shows that the low-temperature zero-field polarization direction lies along this direction). As detailed in the Methods, a single sample of STO//DSO was used to obtain all data, with a few exceptions that do not affect numerical results in the main article. Optical images of STO//DSO with IDEs appear in Supplementary Note 2. Electrostatic simulations in Supplementary Note 3 confirm that the electric field lies primarily in the STO film plane, and varies in magnitude by just 3%. Thus the application of a voltage *V* across the IDE gap may be considered to result in a scalar electric field *E* = *V*/*g*.

**Fig. 2:**
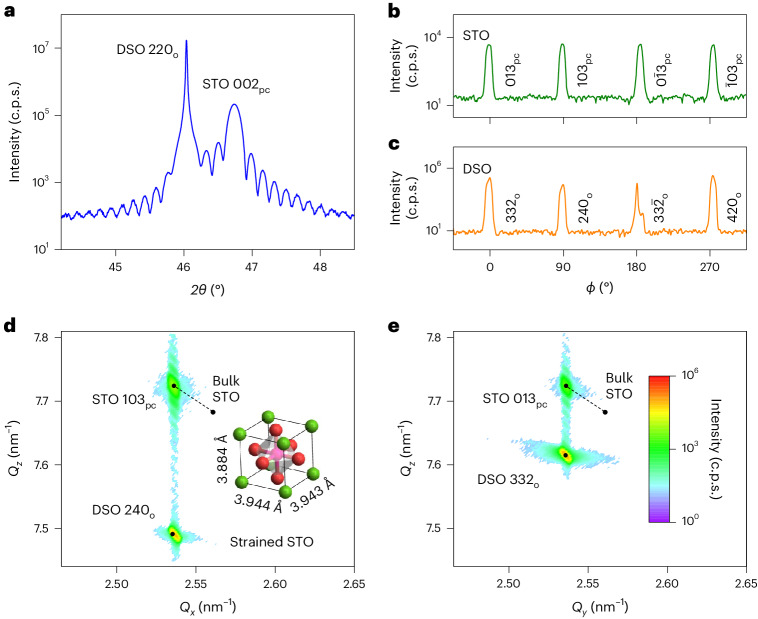
XRD measurements of STO//DSO. **a**, A 2*θ* – *ω* scan (c.p.s., counts per second). The STO 002_pc_ reflection implies an out-of-plane film lattice parameter of *c*_pc_ = 3.884 ± 0.001 Å. The Laue fringes imply a film thickness of 58.2 ± 0.5 nm. **b**, STO *ϕ* scan obtained at 2*θ* = 77.48°. **c**, DSO *ϕ* scan obtained at 2*θ* = 76.35° in the range –45° ≤ *ϕ* ≤ 45° (332_o_ reflection), 75.08° in the range 45° < *ϕ* ≤ 135° (240_o_ reflection), 76.35° in the range 135° < *ϕ* ≤ 225° ($33\bar{2}_{\mathrm{o}}$ reflection) and 77.71° in the range 225° < *ϕ* ≤ 315° (420_o_ reflection). **d**,**e**, Reciprocal space maps (intensity scale in **e**) reveal that most of the film is fully strained with in-plane lattice parameters of *a*_pc_ = 3.944 ± 0.001 Å (**d**) and *b*_pc_ = 3.943 ± 0.001 Å (**e**) (*Q*_*x*_, *Q*_*y*_ and *Q*_*z*_ correspond to inverse lattice spacings along Cartesian axes, *z* is out-of-plane). Black dots calculated from pseudocubic STO film lattice parameters (inset of **d**); DSO substrate lattice parameters (*a*_o_ = 5.440 Å, *b*_o_ = 5.713 Å, *c*_o_ = 7.887 Å)^25^; and the bulk STO lattice parameter (*a*_c_ = 3.905 Å)^43^. Dashed lines link black dots for the STO film and bulk STO. Data for sample 1 obtained prior to IDE deposition.

High-resolution X-ray diffraction (XRD) confirms that the unelectroded STO film is single phase and epitaxially strained (Fig. 2). A 2*θ* *–* *ω* scan showing the 220_o_ DSO and 002_pc_ STO reflections confirms that the film is (001)_pc_ oriented (Fig. 2a) (the diffracted X-ray beam lies at 2*θ* to the incident beam, which lies at *ω* to the film plane). For the 002_pc_ STO reflection, we find an out-of-plane film lattice parameter (*c*_pc_ = 3.884 ± 0.001 Å) that implies compressive out-of-plane strain (–0.5%), while nearby Laue fringes imply a film thickness of 58.2 ± 0.5 nm. Supplementary Note 4 shows the 003_pc_ STO reflection (which also implies *c*_pc_ = 3.884 ± 0.001 Å); small-angle reflectivity data (which imply a similar film thickness of 59 ± 2 nm); the narrow 002_pc_ STO rocking curve of width 0.06°; and the narrower 220_o_ DSO rocking curve of width 0.01°. The scans of azimuthal angle *ϕ* shown in Fig. 2b,c confirm that the in-plane [100]_pc_ and [010]_pc_ directions in the STO film are aligned with the ${[\overline{1}10]}_{{\mathrm{o}}}$ and [001]_o_ directions in the DSO substrate, respectively.

XRD reciprocal space maps (Fig. 2d,e) show that most of the STO film experiences a biaxial in-plane strain of ~1%, and that similar in-plane lattice parameters (*a*_pc_ = 3.944 ± 0.001 Å from Fig. 2d, *b*_pc_ = 3.943 ± 0.001 Å from Fig. 2e) match the expected values (Fig. 1d) with a precision that appears to belie experimental error. For both in-plane directions, a small relaxation towards the bulk STO lattice parameter (intensity near top-left of dashed lines, Fig. 2d,e) is likely associated with the free surface. The out-of-plane STO lattice parameter obtained from both reciprocal space maps is *c*_pc_ = 3.884 ± 0.001 Å, which matches our finding from the 2*θ* – *ω* scan (Fig. 2a). Note that the ~1% strain along the in-plane [100]_pc_ and [010]_pc_ directions, and the approximately –0.5% strain along the out-of-plane [001]_pc_ direction, imply a STO Poisson’s ratio of 0.2 (Supplementary Note 5), consistent with previous reports^45^.

**Fig. 3:**
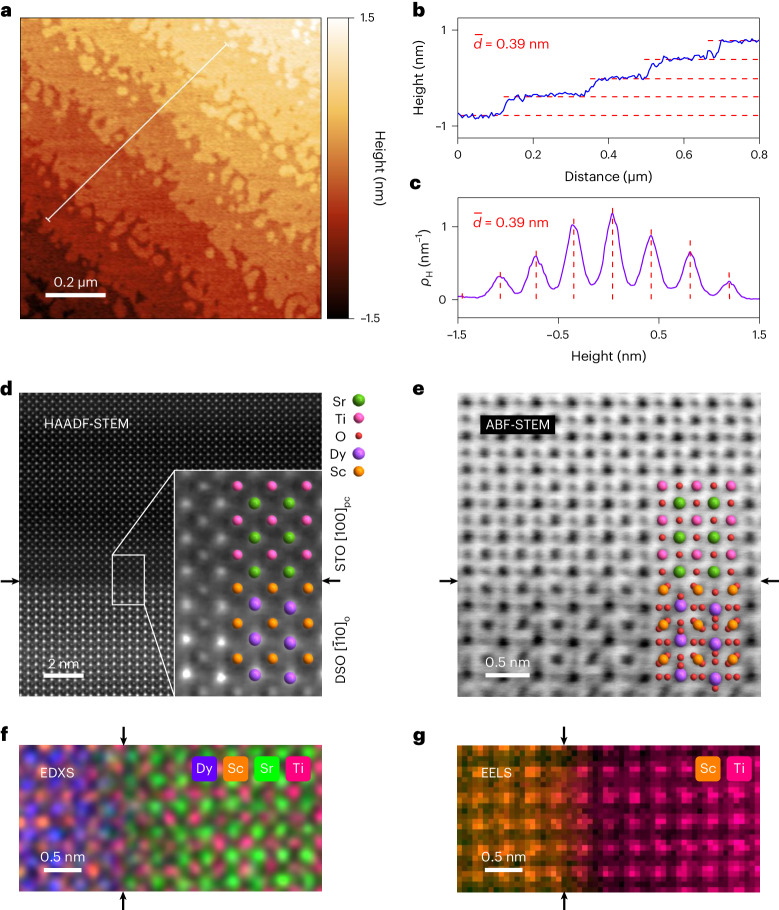
Surface and cross-sectional images of STO//DSO. **a**–**c**, Atomic terraces of average step height $\bar{d} = 0.39 \ {\mathrm{nm}}$ are observed via a 1 μm × 1 μm AFM image (**a**), a transect along the white line in this image (**b**) and the height distribution density function *ρ*_H_ for this image (**c**). **d**–**g**, Visualization of the ScO_2_–SrO interface (arrowed), with the colour code in **d**. **d**,**e**, HAADF-STEM image (**d**) and ABF-STEM image (**e**) down the collinear STO [100]_pc_ and DSO ${\left[\bar{1}10\right]}_{\text{o}}$ zone axes, with a zoomed-in view in the inset in **d** and coloured atoms overlayed. O atoms are readily visible in **e**. **f**, Atomically resolved EDXS map. **g**, Atomically resolved EELS map at the Sc L_2,3_ edge (orange) and the Ti L_2,3_ edge (pink). Large-area AFM, HAADF-STEM and ABF-STEM images appear in Supplementary Note 6. AFM data for virgin sample 1 prior to IDE deposition. STEM data for sample 3.

A 1 μm × 1 μm atomic force microscopy (AFM) image reveals smooth terraces that evidence layer-by-layer STO film growth (Fig. 3a). The terraces are separated by steps whose unit cell height (0.39 ± 0.01 nm) we identify using both a specific transect (Fig. 3b) and the entire image (Fig. 3c). A high-angle annular dark-field (HAADF) scanning transmission electron microscopy (STEM) image (Fig. 3d) reveals good epitaxy with no dislocations, a smooth DSO–STO interface, ScO_2_ (B-site) termination for DSO and SrO (A-site) termination for STO. These observations are equally apparent from an annular bright-field (ABF) scanning transmission electron microscopy image (Fig. 3e), where one also sees oxygen atoms and thus rotated ScO_6_ octahedra^29^. Subjecting part of the HAADF-STEM image to a fast Fourier transform (Supplementary Fig. 6c,d) yields film lattice parameters of *b*_pc_ = 3.94 ± 0.07 Å and *c*_pc_ = 3.88 ± 0.07 Å, which agree with the aforementioned XRD values to within error. The aforementioned interfacial cation terminations are also apparent from an energy-dispersive X-ray spectroscopy (EDXS) map (Fig. 3f) that resolves the A-site Dy and Sr cations well, and an electron energy-loss spectroscopy (EELS) map (Fig. 3g) that resolves only the B-site Sc and Ti cations.

## Electrical measurements

As detailed in the Methods, we obtained dielectric measurements with an impedance analyser and polarization measurements with a ferroelectric tester. Combining the conformally mapped contributions of the constituent layers including air (Supplementary Note 7) permits us to convert the capacitance *C*_meas_(*T*) and charge *Q*_meas_(*T*,*E*) measured for DSO with IDEs into the dielectric response *ε*_sub_(*T*) and polarization *P*_sub_(*T*,*E*) of the DSO substrate (Supplementary Note 8), such that we may then convert the capacitance *C*_meas_(*T*,*E*) and charge *Q*_meas_(*T*,*E*) measured for STO//DSO with IDEs into the dielectric response *ε*_film_(*T*,*E*) and polarization *P*_film_(*T*,*E*) of the STO film (Supplementary Note 9). Supplementary Note 9 also shows that if the electrical data for DSO are simply subtracted from the electrical data for STO//DSO then one obtains similar plots of *ε*_film_(*T*,*E*) and *P*_film_(*T*,*E*), implying that the subtractive method is approximately valid (although only the conformal method returns the correct value of *ε*_sub_ ≈ 21 at room temperature; Supplementary Note 8). The conformal method is also used to convert the capacitance *C*_meas_(*T*,*E*) and charge *Q*_meas_(*T*,*E*) measured for bulk STO with IDEs into the dielectric response *ε*_bulk_(*T*,*E*) and polarization *P*_bulk_(*T*,*E*) of bulk STO (Supplementary Note 10).

**Fig. 4:**
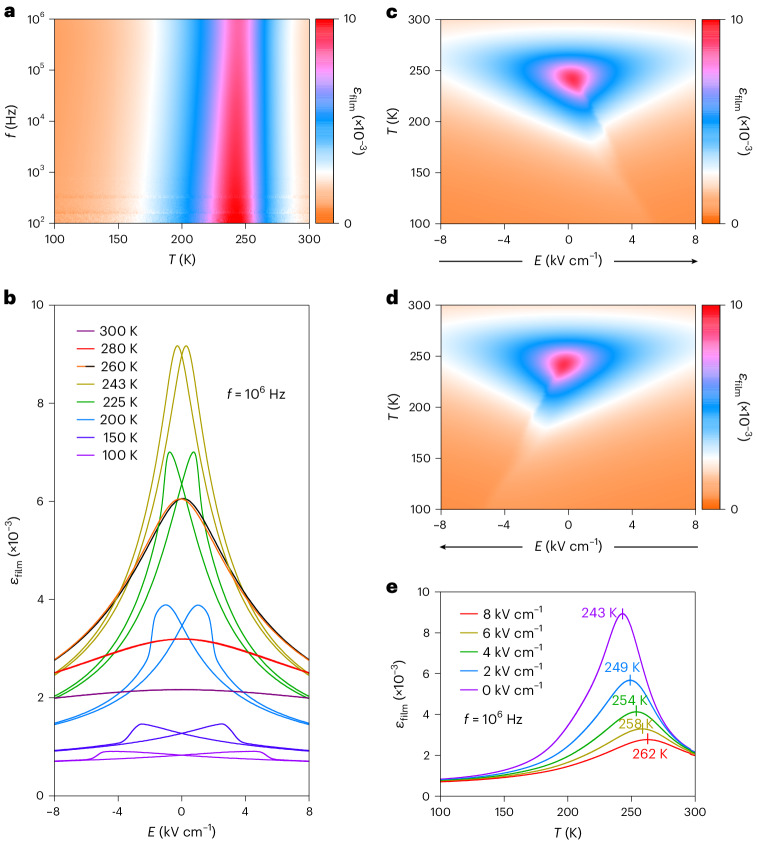
In-plane dielectric response of the strained STO film. **a**, Dielectric response *ε*_film_(*T*,*f*) based on isothermal *ε*_film_(*f*) measured in zero bias at 1,614 values of temperature *T* and 200 values of frequency *f*. We choose to identify *T*_C_ ≈ 243 K via the peak at an intermediate frequency. **b**–**e**, Plots constructed from isothermal *ε*_film_(*E*) with 200 data points obtained at 10^6^ Hz while sweeping the bias field at *f* ≈ 1 Hz. **b**, Bipolar *ε*_film_(*E*) at eight of 4,375 measurement temperatures. The weak hysteresis at 260 K is distinguished using orange (black) for the downfield (upfield) sweep. **c**,**d**, The *ε*_film_(*E*,*T*) constructed from bipolar *ε*_film_(*E*) at all 4,375 measurement temperatures for upfield (**c**) and downfield (**d**) sweeps (indicated by arrows). **e**, Isofield *ε*_film_(*T*) transects through **d** at positive bias fields (the corresponding isofield measurements are similar; Supplementary Fig. 23e). All data measured on cooling. Data based on measurements of sample 1 and DSO substrate 1. Source data

The dielectric response *ε*_film_(*T*,*f*) of the film was identified from zero-field cooling data obtained at 200 values of frequency *f* (Fig. 4a; Supplementary Note 11 shows *ε*_film_(*f*) and *ε*_film_(*T*) transects). The smooth peak in *ε*_film_(*T*) at each frequency evidences a second-order transition, while a weak relaxor component^30,34^ is evidenced by both the weak frequency dependence just below the peak temperature and a small, reproducible thermal hysteresis (Supplementary Note 11). The position of the peak in *ε*_film_(*T*) shifts from 242.0 K to 244.2 K across our range of measurement frequencies, implying a small uncertainty in the Curie temperature, and we choose to identify *T*_C_ ≈ 243 K near the middle of this range.

Our highest frequency (10^6^ Hz) is used for subsequent dielectric measurements because the noise is lowest (Supplementary Note 11). At this frequency, the dielectric loss due to the weak relaxor behaviour peaks near 2% in zero field (Supplementary Fig. 23c) and near 4% in a finite field (Supplementary Fig. 24). These loss tangents respresent an order of magnitude improvement over previous reports^25,28,30,34^, consistent with our AFM and STEM images that evidence large areas of excellent crystalline quality (the images in Fig. 3 are complemented by large-area images in Supplementary Fig. 6).

Bipolar cycles at eight measurement temperatures reveal that *ε*_film_(*E*) (Fig. 4b) displays a peak near *T*_C_ that is split below *T*_C_ due to ferroelectric hysteresis. This peak and its splitting below *T*_C_ can also be appreciated by plotting *ε*_film_(*E*,*T*) separately for up (Fig. 4c) and down (Fig. 4d) field sweeps that were obtained isothermally at all 4,375 measurement temperatures. Isofield *ε*_film_(*T*) transects through Fig. 4d at selected values of *E* ≥ 0 are shown in Fig. 4e. At zero bias field, the *ε*_film_(*T*) peak happens to coincide with our chosen value of *T*_C_ = 243 K, despite the discrepancies of measurement frequency and electrical history^29^. At finite bias fields, the peak magnitude is suppressed and the peak temperature *T*_0_(*E*) is upshifted due to the suppression of thermal fluctuations. (This upshift does not represent a field-induced shift of the phase transition, as the phase transition is second order.)

**Fig. 5:**
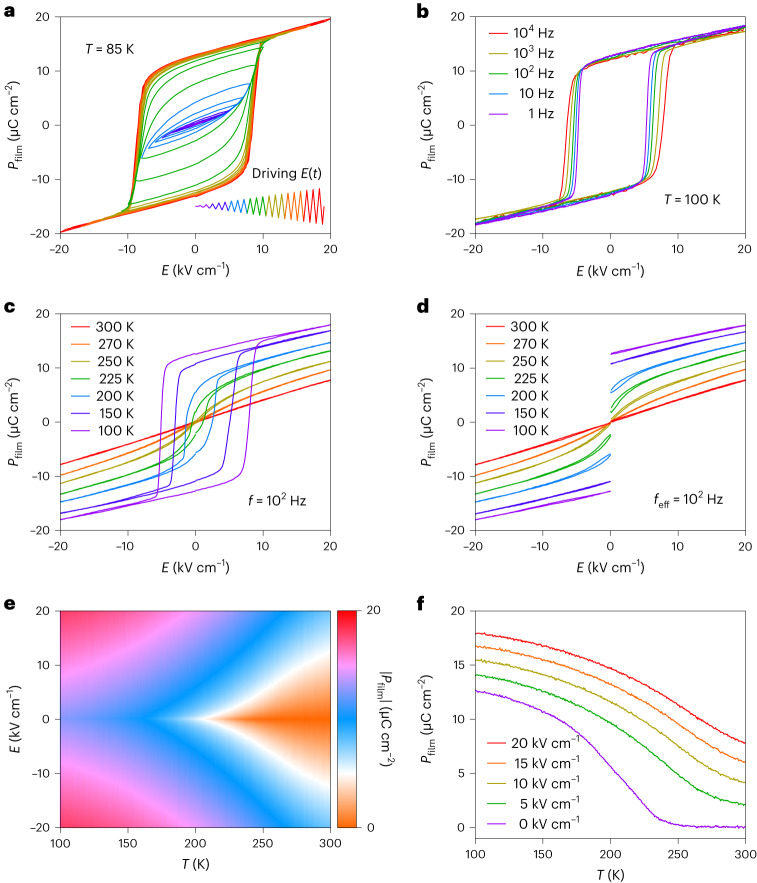
Electrical polarization of the strained STO film. **a**, Bipolar *P*_film_(*E*) at 85 K on increasing the magnitude of *E*(*t*) (inset; *t*, time) by 1 kV cm^–1^ in each successive cycle of period 10 ms. **b**, Bipolar *P*_film_(*E*) at 100 K for selected values of *f*. **c**,**d**, For seven of 668 measurement temperatures, we show bipolar *P*_film_(*E*) with 1,000 data points (**c**) and unipolar *P*_film_(*E*) with 500 data points (**d**), obtained using field-sweep rates of the same magnitude (*f* = 10^2^ Hz in **c**; effective frequency *f*_eff_ = 10^2^ Hz in **d** as shown in Supplementary Note 12). **e**, The |*P*_film_(*T*,*E*)| from field-removal branches of all unipolar *P*_film_(*E*) plots. **f**, Isofield *P*_film_(*T*) transects through **e** at zero field and selected positive fields. Data in **a** and **b** measured after zero-field cooling. Data in **c**–**e** measured on heating. Data based on measurements of sample 1 and DSO substrate 1. Source data

Increasing the amplitude of an a.c. electric field up to 20 kV cm^–1^ is more than sufficient to generate a major *P*_film_(*E*) loop at 85 K (Fig. 5a), and we used this amplitude for all subsequent polarization measurements. On varying the frequency between 1 Hz and 10^4^ Hz at 100 K (Fig. 5b), the high-field polarization shows little variation with frequency due to low leakage, while the coercive field shows some frequency variation due to switching kinetics^46^ and/or current decay after each voltage step^47^.

Bipolar (Fig. 5c) and unipolar (Fig. 5d) polarization data *P*_film_(*E*) were obtained sequentially (Supplementary Note 12) after heating to each of our 668 isothermal measurement temperatures in the range 100–300 K, and data for seven such measurement temperatures appear in Fig. 5c,d (similar cooling data could equally be used to identify EC effects associated with the second-order transition). At 100 K, the bipolar loop is reasonably square, such that the remanent polarization of 12.5 μC cm^–2^ (similar to previous reports^28,34^) represents the spontaneous polarization, which we use in our Landau parameterization. At higher temperatures, loop squareness, height and width are all reduced. Above the Curie temperature at 250 K, the small finite remanence may arise because of the weak relaxor behaviour (Fig. 4a) and/or the small dielectric loss (Supplementary Note 11).

The unipolar *P*_film_(*E*) plots (Fig. 5d) match well the outer branches of the corresponding bipolar plots (Fig. 5c) and show good reversibility at all temperatures. This good reversibility is important in three ways. First, it confers good reversibility on the corresponding EC effects of interest. Second, it implies that the outer branches of our 668 unipolar plots yield a single-valued |*P*_film_(*T*,*E*)| map (Fig. 5e) for evaluating EC effects via the indirect method. Third, it implies that ferroelectric domains do not compromise the Landau assumption of homogeneous film polarization. Cross-sections of the |*P*_film_(*T*,*E*)| map at zero field and selected positive fields yield isofield *P*_film_(*T*) plots (Fig. 5f). The zero-field *P*_film_(*T*) plot is consistent with a second-order transition, but the aforementioned remanence in the paraelectric phase precludes accurate determination of the Curie temperature.

## Electrocaloric effects

**Fig. 6:**
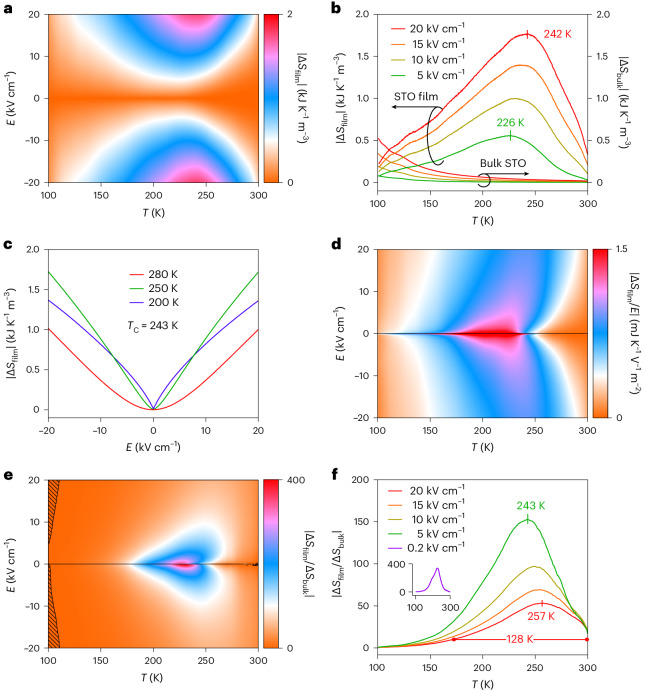
EC effects in the strained STO film. **a**, $|\Delta S_{\mathrm{film}}(T,E)| = |{\int }_{0}^{E} (\partial P_{{\mathrm{film}}} / \partial T)_{{E}^{\prime}} {\mathrm{d}}E^{\prime}|$ derived from Fig. 5e. **b**, Four isofield |Δ*S*_film_(*T*)| transects through **a** at selected positive fields, and the corresponding data for bulk STO (Supplementary Fig. 18b). **c**, Isothermal |Δ*S*_film_(*E*)| transects through **a** at selected temperatures. **d**, EC strength |Δ*S*_film_(*T*,*E*)/*E*|. **e**, |Δ*S*_film_(*T*,*E*)/Δ*S*_bulk_(*T*,*E*)| is larger (smaller) than unity in unhashed (hashed) regions (interpolation yields data intervals of 1 K and 0.02 kV cm^–1^). **f**, Isofield transects through **e** at positive fields represent the ratio of isofields such as those in **b** (inset shows the 0.2 kV cm^–1^ transect). Data based on measurements of sample 1, DSO substrate 1 and a single sample of bulk STO (Supplementary Note 10). Source data

We use the indirect method^4^ to determine the reversible field-driven isothermal entropy change $|\Delta S_{\mathrm{film}}(T,E)| = |{\int }_{0}^{E} (\partial P_{{\mathrm{film}}} / \partial T)_{{E}^{\prime}} {\mathrm{d}}E^{\prime}|$ (Fig. 6a) by smoothing the unipolar |*P*_film_(*T*,*E*)| data (Fig. 5e) to generate |*P*_film_(*T*)| spline fits at each measurement field (selected *P*_film_(*T*) spline fits appear in Figure 5f; the relevant Maxwell relation (∂*P*_film_/∂*T*)_*E*_ = (∂*S*_film_/∂*E*)_*T*_ is valid given the aforementioned reversibility; *E*′ is the dummy variable of integration). Isofield |Δ*S*_film_(*T*)| transects at positive fields (Fig. 6b) show that increasing the field expands the |Δ*S*_film_(*T*)| peak, whose maximum (|Δ*S*_film_| ≈ 1.8 kJ K^–1^ m^–3^) dwarfs corresponding values for bulk STO (Fig. 6b; data from Supplementary Note 10) and the DSO substrate (|Δ*S*_sub_| ≈ 10^–4^ kJ K^–1^ m^–3^; Supplementary Note 13). We elaborate on the comparison with bulk STO in the next paragraph. For the film, Supplementary Note 14 discusses isothermal |Δ*S*_film_(*E*)| transects (Fig. 6c) through |Δ*S*_film_(*T*,*E*)| (Fig. 6a); Supplementary Note 15 discusses isothermal and isofield transects through a map of EC strength |Δ*S*_film_(*T*,*E*)/*E*| (Fig. 6d); Supplementary Note 16 describes adiabatic temperature change; Supplementary Note 17 presents EC data constructed from polarization data obtained by integrating dielectric data (Fig. 4c,d); and Supplementary Note 18 demonstrates reproducibility.

The EC effects in our strained film are much larger than EC effects in bulk STO in a range of temperatures above 100 K, as seen from |Δ*S*_film_(*T*,*E*)| and |Δ*S*_bulk_(*T*,*E*)| in Fig. 6b, their quotient |Δ*S*_film_(*T*,*E*)/Δ*S*_bulk_(*T*,*E*)| in Fig. 6e and its positive-field transects in Fig. 6f. For our largest field of 20 kV cm^–1^, EC effects experience an order of magnitude strain-induced enhancement (|Δ*S*_film_/Δ*S*_bulk_| ≥ 10) that extends over more than 128 K, persists at room temperature and peaks to |Δ*S*_film_/Δ*S*_bulk_| = 53 at 257 K. This high-field enhancement represents our main result. For the smaller field of 0.2 kV cm^–1^ (inset, Fig. 6f), EC effects experience a two order of magnitude strain-induced enhancement (|Δ*S*_film_/Δ*S*_bulk_| ≥ 100) that persists over 75 K and peaks to |Δ*S*_film_/Δ*S*_bulk_| = 339 at 226 K.

Bespoke Landau predictions do well at reproducing the dielectric response, polarization and EC effects in our strained STO film (Supplementary Note 19) and bulk STO (Supplementary Note 20). Given that |Δ*S*_bulk_| grows on cooling towards 100 K (Fig. 6b), we extended our bulk STO Landau predictions below 100 K by building on Supplementary Note 19 with parameters for the 105 K antiferrodistortive transition from the literature^48^. We find a good match with cryogenic results (|Δ*S*_bulk_| ≈ 0.1 kJ K^–1^ m^–3^ with 7 kV cm^–1^ at 20 K)^23^, and for our maximum field of 20 kV cm^–1^ we find a peak (|Δ*S*_bulk_| ≈ 1 kJ K^–1^ m^–3^ at 40 K) that is smaller than our film peak (|Δ*S*_film_| ≈ 1.8 kJ K^–1^ m^–3^ at 242 K). Therefore peak EC effects in our strained film exceed the corresponding peak EC effects in bulk STO.

## Outlook

Epitaxial oxide films present large areas of high-quality crystalline material in a single orientation, but are rarely used to study EC effects^37–39^ even though EC effects are not well understood. Our demonstration that epitaxial strain yields highly reversible extrinsic EC effects over a wide temperature range immediately implies that extrinsic EC effects should be investigated in many systems, via Landau theory and experimentally. Fixed ~1% strains can be achieved in epitaxial films on single-crystal substrates, and variable strains reaching this magnitude can be achieved in epitaxial films after transfer from lattice-matched growth substrates to electroactive substrates^49^. Excitingly, epitaxially grown oxide films can experience much larger strains (4–10%) after transfer to electrically driven electroactive polymers^50^, magnetically driven magnetostrictive metals^51^ and mechanically driven polymers^26,52^. Active volumes may be increased by fabricating multilayers, where one type of layer generates strain and might also display caloric effects, while the other type of layer displays strain-induced EC effects of the type we report here.

Our work could also inspire the development of stress-induced EC effects in bulk oxides. If the stress is variable then the resulting elastocaloric effects could be enhanced by the electric field used to drive EC effects. The use of two fields is well known in the growing body of work on multicalorics, where hysteresis can be transferred from one field variable to another, reducing the magnitude of each field and thus reducing the risk of breakdown^53^. Overall, our approach promises to expand the limited library of EC materials and will hopefully inspire others to apply our method for converting poor EC materials into good EC materials.

## Methods

### Samples

Sample 0 was used to determine the orientation of the in-plane polarization (Supplementary Note 1). All other data were obtained using sample 1 with the following exceptions: scanning electron microscopy data were obtained using sample 2 (Supplementary Note 2); STEM data were obtained using sample 3 with no IDEs; and EC data for sample 1 were reproduced using samples 2 and 4 (Supplementary Note 18). The IDEs on sample 1 were deposited after collecting first AFM data, and then XRD data.

### Film growth

A flowing oxygen ambient of 10 Pa was used to pre-anneal DSO (110)_o_ substrates for 30 min at 800 °C before depositing STO at 760 °C via pulsed laser deposition with a KrF excimer laser (wavelength *λ* = 248 nm; 1 Hz; 113 mJ over 8.6 mm^2^ and thus 1.3 J cm^–2^ per pulse). After deposition, we reduced the temperature at a rate of –5 °C min^–1^ to 700 °C, annealed for 1 h in 50 kPa of oxygen and then cooled to room temperature at –10 °C min^–1^. All STO films were nominally ~60 nm thick. Laue fringes associated with the 002_pc_ STO reflection were used to determine the following film thicknesses for calculations: 56.1 ± 0.5 nm (sample 0), 58.2 ± 0.5 nm (sample 1), 57.4 ± 0.5 nm (sample 2) and 56.7 ± 0.5 nm (sample 4). The DSO substrate of sample 0 measured 5 mm × 5 mm × 0.5 mm. All other DSO substrates, including the electroded substrate with no film (Supplementary Note 8), measured 3 mm × 5 mm × 0.5 mm.

### Electrode preparation

IDEs were printed with an aerosol jet printer (Optomec Aerosol Jet 200) using silver nanoparticle ink (Clariant PRELECT TPS 50 Nano Ag ink) mixed 1:1 with deionized water. A N_2_ flow of 30 sccm drove the sonicated ink mist towards a 150-μm-diameter printing tip, where the sheath flow was 55 sccm. A printing speed of 1 mm s^–1^ resulted in 2-µm-thick IDEs (width, 25 µm; gap, 50 µm). Annealing at 200 °C for 3 h burned off ink surfactant to improve conductivity. More information about the method appears in ref. ^54^. Optical images of the IDEs appear in Supplementary Notes 1 and 2.

### X-ray diffraction

We used a PANalytical Empyrean high-resolution X-ray diffractometer with a primary monochromator that selects Cu Kα_1_ radiation (*λ* = 1.540598 Å). A positive offset (*ω* > 2*θ* – *ω*) was adopted for the asymmetric XRD reciprocal space map measurements to compress the diffraction beam for better 2*θ* resolution.

### Atomic force microscopy

STO surface topography was probed in tapping mode using a Veeco Digital Instruments AFM instrument equipped with a Nanoscope V controller. Second-order flattening and/or plane fitting removed apparent variations of height over length scales that are long compared with terrace spacing.

### Electron microscopy

Scanning electron microscopy data were obtained using a Hitachi TM3030. STEM imaging, EDXS elemental mapping and EELS spectrum imaging were performed using an aberration-corrected FEI Titan Themis G2 microscope operated at 300 kV. The convergent semi-angle was 25 mrad for both STEM imaging and EELS mapping. The collection angle was 48–200 mrad for HAADF and 12–45 mrad for ABF. Two-dimensional EDXS images were acquired based on the Super-X EDXS system (four-quadrant SDD EDXS detector), which has a high signal-collection efficiency for fast EDXS mapping. Two-dimensional EELS maps of the Sc L edge and the Ti L edge were performed with a dispersion of 0.25 eV per channel. Core-loss peak positions were calibrated using a simultaneously acquired zero-loss spectrum in DualEELS.

### Variable-temperature electrical measurements

We performed two-terminal dielectric and electrical polarization measurements using a homemade cryogenic probe^55^ whose sample stage was covered with electrically insulating Kapton tape. Evacuation reduced the pressure to 0.07 mbar at room temperature, and subsequent cryopumping with liquid nitrogen reduced the pressure further to 0.05 mbar. We used a Lakeshore 336 temperature controller to achieve average ramp rates of ±1 K min^–1^, which are slow enough to collect electrical data at nominally constant temperatures that we identify with a resolution of 0.2 K or better.

### Dielectric measurements

Dielectric measurements were performed using an Agilent 4294A impedance analyser in the ‘Parallel Capacitance and Dissipation Factor’ mode to obtain the measured capacitance *C*_meas_, which we converted into the dielectric response *ε*_film_ of the STO film under study (Supplementary Notes 7–10). The root mean square amplitude of the sinusoidal driving voltage was 1 V, implying 0.28 kV cm^–1^. The maximum d.c. bias voltage was 40 V, implying 8 kV cm^–1^.

### Electrical polarization measurements

Electrical polarization measurements were performed using a Radiant Precision Premier II to obtain the measured charge *Q*_meas_, which we converted into the polarization *P*_film_ of the STO film under study (Supplementary Notes 7–10). The maximum applied voltage was 100 V, implying 20 kV cm^–1^.

## Online content

Any methods, additional references, Nature Portfolio reporting summaries, source data, extended data, supplementary information, acknowledgements, peer review information; details of author contributions and competing interests; and statements of data and code availability are available at 10.1038/s41563-024-01831-1.

## Supplementary information

Supplementary Information Supplementary Notes 1–20 and References.

## Source data

Source Data Fig. 4 In-plane dielectric response of the strained STO film data plotted in Fig. 4a–e.

Source Data Fig. 5 Electrical polarization of the strained STO film data plotted in Fig. 5a–f.

Source Data Fig. 6 EC effects in the strained STO film data plotted in Fig. 6a–f.

## Data Availability

Source data are provided with this paper. All other relevant data are available within the paper and its Supplementary Information files.
