# Publisher Correction: Highly reversible extrinsic electrocaloric effects over a wide temperature range in epitaxially strained SrTiO_3_ films

**DOI:** 10.1038/s41563-025-02321-8

**Published:** 2025-07-30

**Authors:** S. Zhang, J. Deliyore-Ramírez, S. Deng, B. Nair, D. Pesquera, Q. Jing, M. E. Vickers, S. Crossley, M. Ghidini, S. Kar-Narayan, G. G. Guzmán-Verri, X. Moya, N. D. Mathur

**Affiliations:** 1 College of Science, National University of Defense Technology, Changsha, China; 2 Department of Materials Science, University of Cambridge, Cambridge, UK; 3 Centro de Investigación en Ciencia e Ingeniería de Materiales (CICIMA), Universidad de Costa Rica, San José, Costa Rica; 4 Escuela de Física, Universidad de Costa Rica, San José, Costa Rica; 5 Beijing Advanced Innovation Center for Materials Genome Engineering, University of Science and Technology Beijing, Beijing, China; 6 James Watt School of Engineering, University of Glasgow, Glasgow, UK; 7 DiFeST, University of Parma, Parma, Italy; 8 Diamond Light Source, Chilton, Didcot, UK

**Keywords:** Ferroelectrics and multiferroics, Phase transitions and critical phenomena, Surfaces, interfaces and thin films

Correction to: *Nature Materials* 10.1038/s41563-024-01831-1, published online 21 March 2024.

The author list, author contributions and acknowledgements have been updated since the initially published version of this article. This Publisher Correction is a response to an agreement resulting from a dispute resolution process at the University of Cambridge. The changes are made in the HTML and PDF versions of the article.

