# Supplementary material for: Highly reversible extrinsic electrocaloric effects over a wide temperature range in epitaxially strained SrTiO3 films
Source: Nat Mater. 2024 Mar 21;23(5):639–47. doi: 10.1038/s41563-024-01831-1 (PMC11068575; doi:10.1038/s41563-024-01831-1)
Supplement: Supplementary file 1 — Supplementary Notes 1–20 and References. [file 41563_2024_1831_MOESM1_ESM.pdf]

nature materials

Supplementary information https://doi.org/10.1038/s41563-024-01831-1

Highly reversible extrinsic electrocaloric effects over a wide temperature range in epitaxially strained $SrTiO_3$ films

In the format provided by the authors and unedited

Supplementary Information for

Highly reversible extrinsic electrocaloric effects over a wide temperature range in epitaxially strained $SrTiO_3$ films

Contents

Note 1. Orientation of in-plane ferroelectric polarization for STO//DSO 2
Note 2. Images of STO//DSO with interdigitated electrodes 3
Note 3. Simulated electric field around two interdigitated electrodes on STO//DSO 4
Note 4. X-ray measurements of STO//DSO 5
Note 5. Poisson's ratio for STO in STO//DSO 6
Note 6. Additional AFM and TEM data for STO//DSO 7
Note 7. Principle of conformal mapping and IDE capacitance formula 9
(1) Conformal mapping of a ferroelectric film with IDEs 10
(2) Contributions to the capacitance of a ferroelectric film measured with IDEs 11
(3) Expression for the measured capacitance of STO//DSO with IDEs 12
Note 8. Permittivity and polarization of the DSO substrate 14
(1) $C_{meas}(T)$ for DSO → $\varepsilon_{sub}(T)$ for DSO 14
(2) $Q_{meas}(T,E)$ for DSO → $P_{sub}(T,E)$ for DSO 14
Note 9. Dielectric response and polarization of the STO film 16
(1) $C_{meas}(T,E)$ for STO//DSO → $\varepsilon_{film}(T,E)$ for STO 16
(2) $Q_{meas}(T,E)$ for STO//DSO → $P_{film}(T,E)$ for STO 18
Note 10. Ferroelectric, EC and dielectric behaviour of bulk STO 19
(1) $C_{meas}(T,E)$ for bulk STO → $\varepsilon_{bulk}(T,E)$ for bulk STO 19
(2) $Q_{meas}(T,E)$ for bulk STO → $P_{bulk}(T,E)$ for bulk STO 20
(3) Ferroelectric and EC measurements for bulk STO 21
(4) EC effects in bulk STO via polarization from integrated dielectric data 23
Note 11. Dielectric response and loss tangent versus DC bias for STO//DSO 28
Note 12. Bipolar and unipolar polarization in STO//DSO 30
Note 13. EC effects in the DSO substrate 31
Note 14. Isothermal transects through $|\Delta S_{film}(T,E)|$ for STO//DSO 32
Note 15. Isothermal and isofield transects through $|\Delta S_{film}/E(T,E)|$ for STO//DSO 33
Note 16. Adiabatic temperature change $|\Delta T_{film}(T_s,E)|$ for the strained STO film 34
Note 17. EC effects in STO//DSO via polarization from integrated dielectric data 37
Note 18. EC effects in different STO//DSO samples 41
Note 19. Landau theory of EC effects in STO//DSO 42
(1) Energy density 43
(2) The stable ferroelectric phase with S_m = +1% 45
(3) The 19 model parameters 47
(4) STO//DSO: predictions of the model versus results derived from experiment 48
Note 20. Landau predictions for bulk STO versus results derived from experiment 54
Supplementary References 58

Note 1. Orientation of in-plane ferroelectric polarization for STO//DSO

Ferroelectric hysteresis loops were measured at 100 K for different geometries (Fig. S1a,b). The measured polarization data (Fig. S1c) were converted to polarization data P_{film} for the film (Fig. S1d) by employing the corrections for background and geometry that we describe in Supplementary Notes 7-9 (after adapting the geometrical corrections for the specific geometries employed here).

For both the measured and corrected polarization data, loop squareness is largest for Device A. Therefore the local zero-field polarization lies parallel/antiparallel to the in-plane $[100]_{pc}$ direction ($a_{pc} > b_{pc}$) and parallel to DSO $[\bar{1}10]_o$.

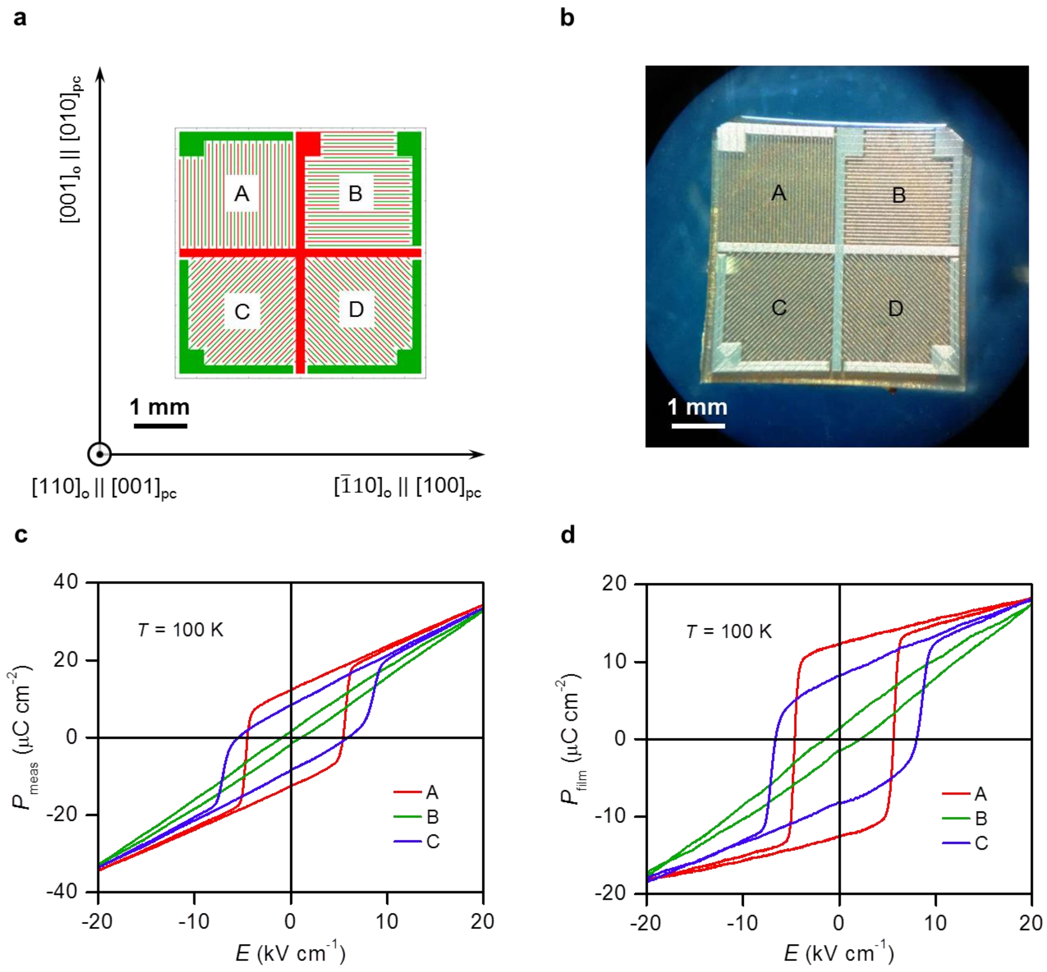

Figure S1. Orientation of in-plane ferroelectric polarization for STO//DSO. (a) Schematic and (b) optical images of Devices A-D, with interdigitated electrodes in four orientations. (c,d) Bipolar plots of (c) measured polarization $P_{meas}(E)$, and (d) film polarization $P_{film}(E)$, for devices A-C at 100 K. Data for Device D, which are similar to data for Device C, are not shown for clarity. Data in (b,c) for Sample 0. Data in (d) for Sample 0 and DSO substrate 1.

Note 2. Images of STO//DSO with interdigitated electrodes

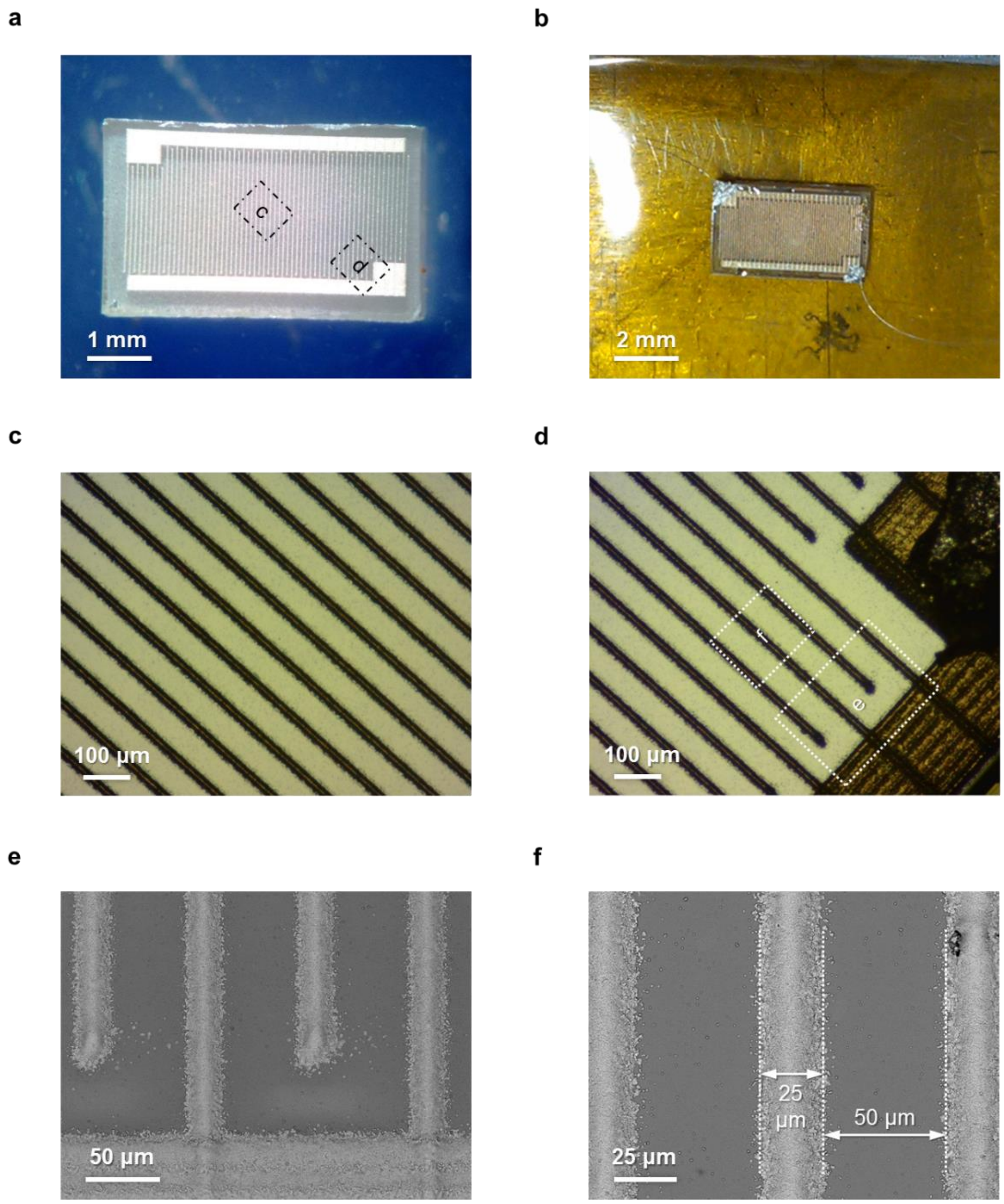

Figure S2. Images of STO//DSO with interdigitated electrodes. (a) Optical image of a pristine sample. (b) Optical image of a sample that has been mounted on a probe for two-terminal electrical measurements via wires attached to contact pads with silver dag (Kapton tape on probe head appears yellow). (c,d) Optical image of the as-marked regions in (a). (e,f) Scanning electron microscopy images of the as-marked regions in (d). Data in (b,c,d) for Sample 1. Data in (a,e,f) for Sample 2.

Note 3. Simulated electric field around two interdigitated electrodes on STO//DSO

Electric fields around the interdigitated electrodes (IDEs) were simulated via finite element analysis using COMSOL Multiphysics®.

Given that the large aspect ratio in each region of addressed film is challenging for both simulation and presentation (electrode separation 50 μm, film thickness ~60 nm), we assume here a film thickness of 1 μm. To increase simulation efficiency, we used a variable element size ranging from 0.012 μm to 6 μm.

The voltage between the IDEs was set to 100 V. The dielectric response of the STO film was taken to be $\varepsilon_{\text{film}}$ = 8000, such that it roughly matches the zero-field peak arising near the Curie temperature of T_C = 243 K (Fig. 4a in the main paper). The dielectric response of the DSO substrate (ε_{sub} = 21) was taken from the data sheet of the vendor (CrysTec). The dielectric response of the pumped air above the sample was taken to be unity.

Fig. S3 shows that the electric field in the addressed STO film lies in-plane and varies by just 3% (19.5 kV $\text{cm}^{-1} \leq |E| \leq$ 20.1 kV cm^{-1}).

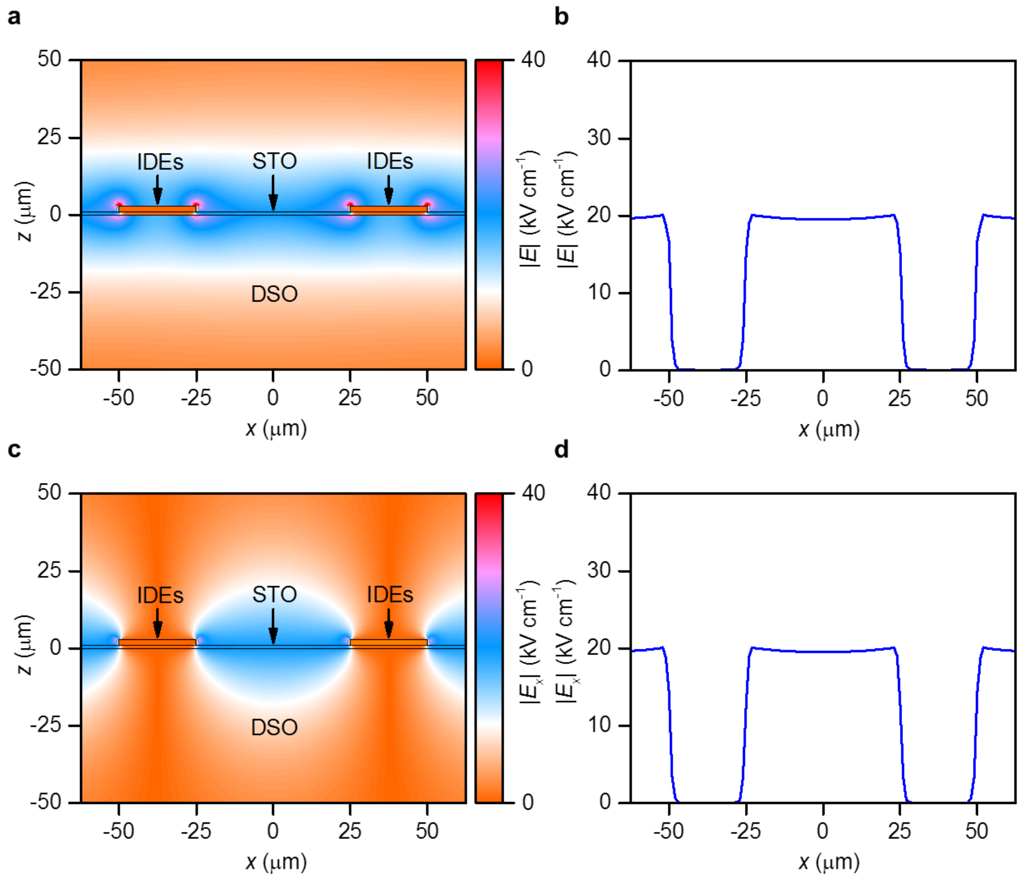

Figure S3. Simulated electric field around two interdigitated electrodes on STO//DSO. For an applied voltage of 100 V, we simulate (a,b) the electric field magnitude $|E|$, and (c,d) the magnitude of the x component $|E_x|$, in (a,c) in the vertical x-z plane that bisects IDEs, and (b,d) after averaging through the STO film. The film in $0 \leq z \leq 1$ μm is addressed by electrodes of cross-section 25 μm × 2 μm and separation 50 μm.

Note 4. X-ray measurements of STO//DSO

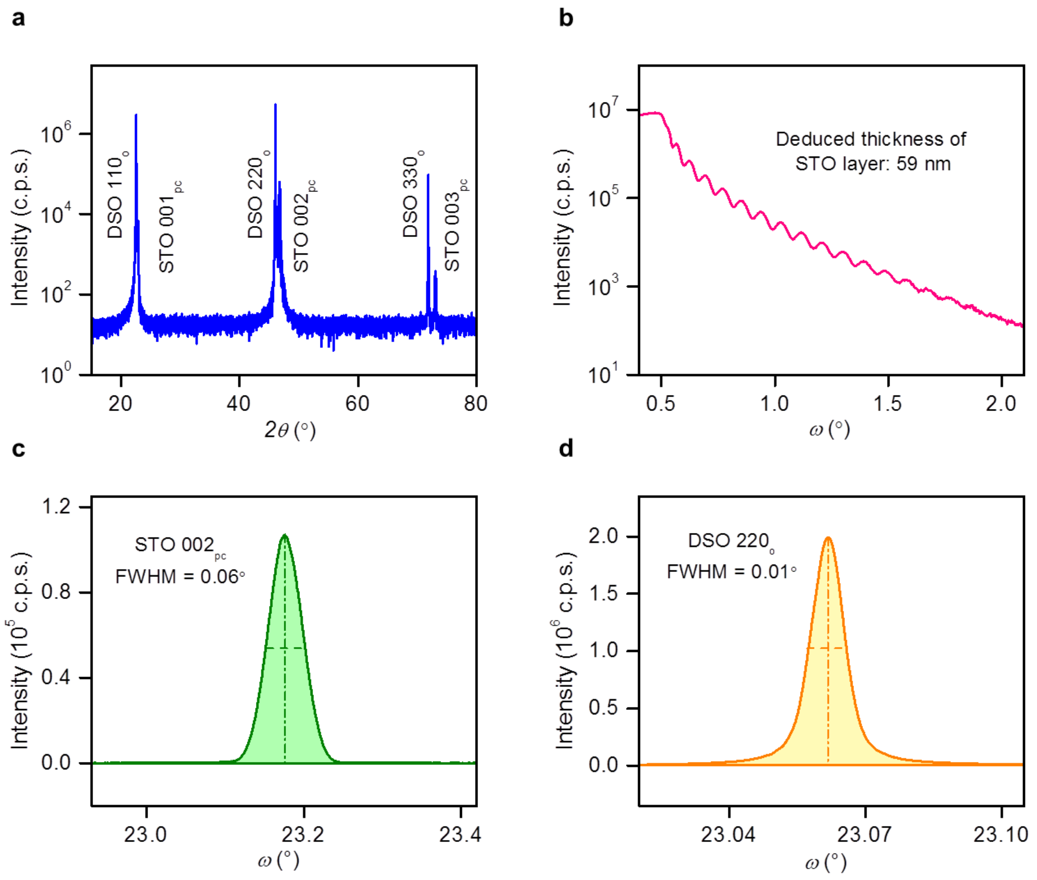

Figure S4. X-ray measurements of STO//DSO. (a) 2θ-ω scan for a wide range of angles compared with Fig. 2a in the main paper. The 002_{pc} and 003_{pc} STO reflections both imply $c_{pc} = 3.884 \pm 0.001$ Å. (b) Small-angle reflectivity data for the STO film implies a thickness (59 ± 2 nm) that is similar to the thickness that we use everywhere else (58.2 ± 0.5 nm from the Laue fringes near the 002_{pc} STO reflection, Fig. 2a in the main paper). (c) Rocking curve with FWHM = 0.06 ° for the 002_{pc} STO reflection in (a). (d) Rocking curve with FWHM = 0.01 °for the 220_{o} DSO reflection in (a). Data for Sample 1.

Note 5. Poisson's ratio for STO in STO//DSO

For an isotropic material, elastic strains $\varepsilon_{x,y,z}$ due to applied stresses $\sigma_{x,y,z}$ depend on the Young's modulus Y and Poisson's ratio v via[S1]:

$$\varepsilon_x = \frac{1}{Y}[\sigma_x - v(\sigma_y + \sigma_z)],\ \ \varepsilon_y = \frac{1}{Y}[\sigma_y - v(\sigma_z + \sigma_x)],\ \ \varepsilon_z = \frac{1}{Y}\,[\sigma_z - v(\sigma_x + \sigma_y)]$$

First, let us consider uniaxial stress with $\sigma_x = \sigma$, and $\sigma_y = \sigma_z = 0$ (Fig. S5a). The equations at top simplify to yield:

$$\varepsilon_x = \frac{\sigma}{Y}\ ,\ \ \varepsilon_y = \varepsilon_z = -v\frac{\sigma}{Y}$$

and eliminating $\frac{\sigma}{Y}$ yields Poisson's ratio $v = -\frac{\varepsilon_{y,z}}{\varepsilon_x}$.

Next, let us consider our epitaxial film with equibiaxial in-plane stress $\sigma_x = \sigma_y = \sigma$, and $\sigma_z = 0$ (Fig. S5b). From the equations at top, we have:

$$\varepsilon_x = \varepsilon_y = \frac{\sigma}{Y}(1 - v),\ \ \varepsilon_z = -2v\frac{\sigma}{Y}$$

and eliminating $\frac{\sigma}{Y}$ yields Poisson's ratio $v = \frac{\varepsilon_z}{\varepsilon_z - 2\varepsilon_x}$.

The approximate values of $\varepsilon_x \sim \varepsilon_y \sim +1\%$ and $\varepsilon_z \sim -0.5\%$ for our STO film yield $v \sim 0.2$, while we obtain $v = 0.21$ from our precise lattice parameters (see the Fig. 2 caption in the main paper) by combining either of the in-plane strains ($\varepsilon_x = 1.008\%$ and $\varepsilon_y = 0.986\%$) with the out-of-plane strain ($\varepsilon_z = -0.538\%$).

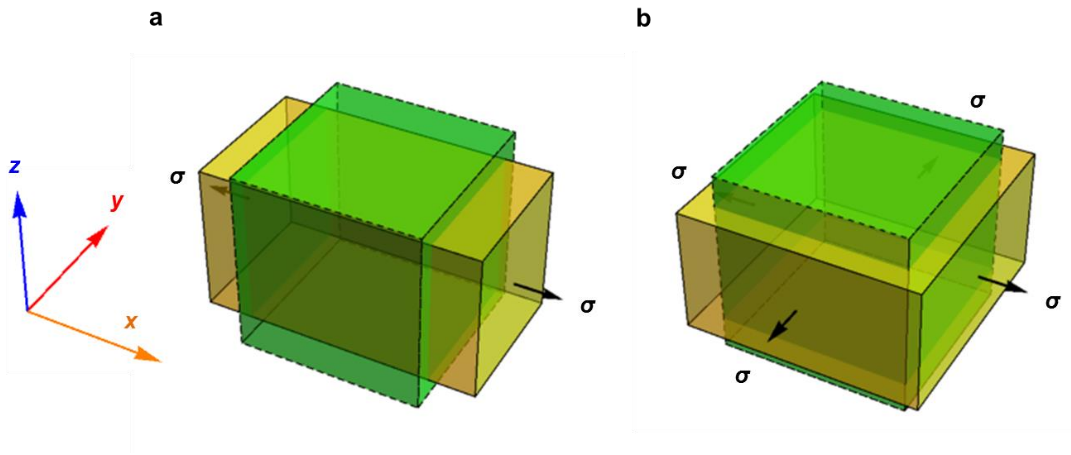

Figure S5. Deformations under uniaxial and equibiaxial stress. An undistorted cube (green) may be distorted (yellow) by (a) uniaxial tensile stress σ, and (b) equibiaxial tensile stress σ. The equibiaxial tensile stress in our epitaxial film lies in the film plane. Diagrams not to scale.

Note 6. Additional AFM and TEM data for STO//DSO

Fig. S6a shows a large-area AFM image.

Fig. S6b shows a large-area ABF-STEM image.

Fig. S6c shows part of a HAADF-STEM image, whose fast Fourier transform (Fig. S6d) yields reciprocal lattice parameters b^*_{pc} ~ 0.2536 Å^{-1} and c^*_{pc} ~ 0.2574 Å^{-1}, corresponding to in-plane and out-of-plane lattice parameters b_{pc} = 3.94 ± 0.07 Å and c_{pc} = 3.88 ± 0.07 Å. These values agree well with b_{pc} = 3.943 ± 0.001 Å and c_{pc} = 3.884 ± 0.001 Å from XRD (Fig. 2d,e in the main paper).

Fig. S6e shows a large-area HAADF-STEM image that is used to map (Fig. S6f) the film and substrate lattice parameters (both denoted b_{pc}) along the direction that lies in the plane of the screen and parallel to the film-substrate interface. The apparent inhomogeneity in the map is due to the relatively large error associated with TEM lattice parameter values. These values are extracted for substrate and film as follows:

- In the substrate, the alternately rotated ScO_6 octahedra[S2,S3] (Fig. 1b of the main paper) yield bimodal pc lattice parameters, which after eliminating the irregular region on the right are found to be b_{pc} = 3.87 ± 0.07 Å and b_{pc} = 4.01 ± 0.07 Å, giving an average value of 3.94 ± 0.07 Å. This value agrees well with the value of b_{pc} ~ 3.943 Å for DSO and thus the STO film (Fig 1d of the main paper).
- In the film, we find b_{pc} = 3.94 ± 0.07 Å, which matches the fast Fourier transform value above.

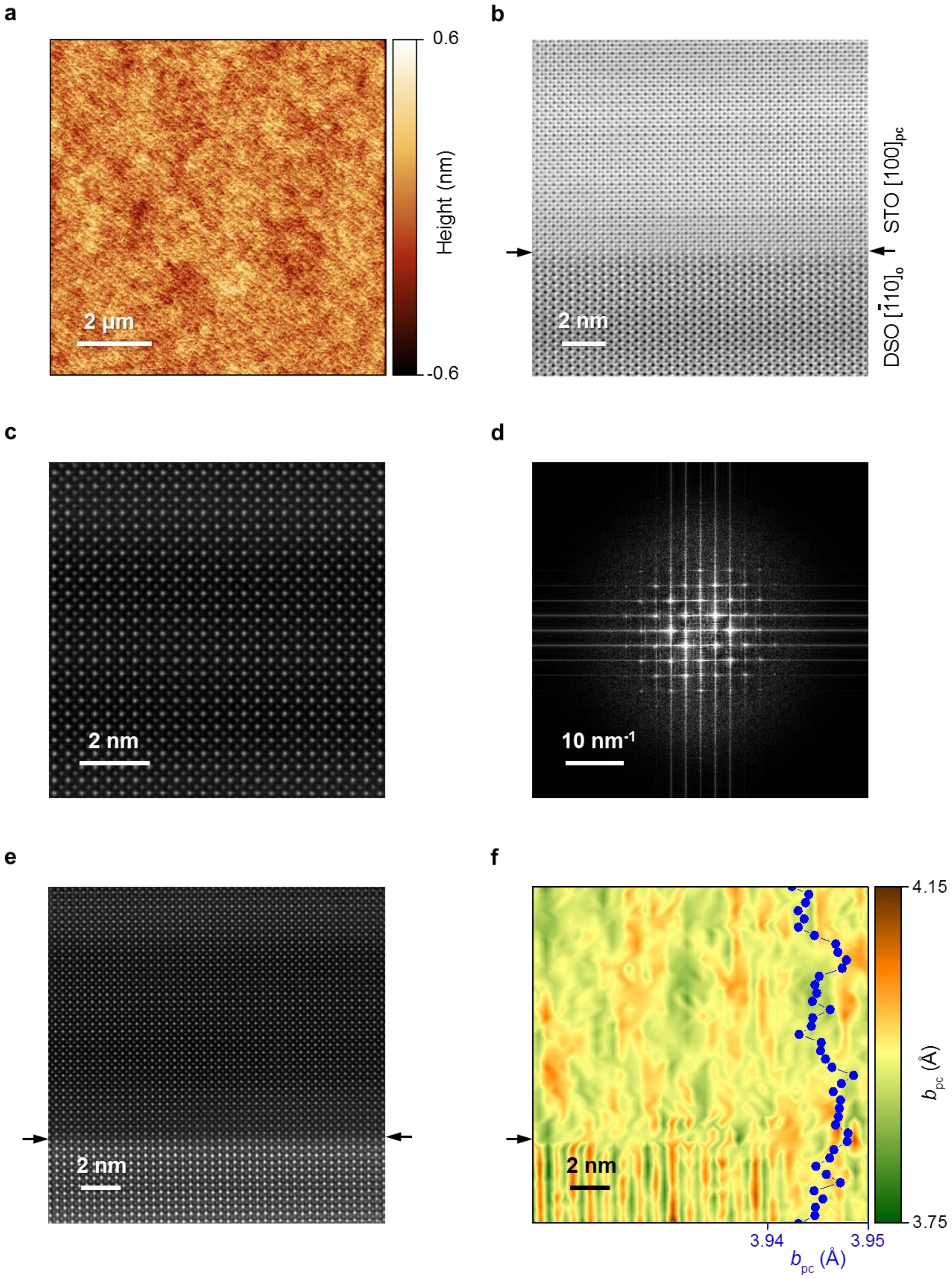

Figure S6. Additional AFM and TEM data for STO//DSO. (a) Large-area (10 µm × 10 µm) flattened AFM image revealing atomic step terraces. (b) Large-area ABF-STEM image looking down the STO $[100]_{pc}$ and DSO $[\bar{1}10]_o$ zone axes near the interface (arrowed). (c) HAADF-STEM image showing the STO film, and (d) its Fast Fourier transform. (e) Large-area HAADF-STEM image, and hence (f) a map of film and substrate in-plane lattice parameters (both denoted b_{pc}) based primarily on Sr and Dy positions (data are interpolated to create a continuous image). Blue dots show the average value of b_{pc} for every row of atomic columns. Data in (a) for Sample 1. Data in (b-f) for Sample 3. The image in (c) is taken from part of the image in Fig. 3d of the main paper. The large-area images in (a,b,e) were obtained independently of the small-area images in Fig. 3a,d,e of the main paper.

Note 7. Principle of conformal mapping and IDE capacitance formula

The work here is based on ref. [S4], and refs [S5,S6] cited therein. After correcting minor errors in refs[S5,S6], we have good agreement with ref. [S7]. The reader may also see ref. [S8] for conformal mapping, and ref. [S9] for the method of combining contributions from the film/substrate/air. Calculations were performed using Mathematica software.

In (1), we show how a single ferroelectric film addressed by IDEs can be mapped to the parallel-plate geometry (the analysis would hold equally well for a dielectric film). In (2), we explain how to calculate the measured capacitance for our hypothetical ferroelectric film, and for simplicity we do not use mathematical formulae yet. In (3), we construct a mathematical expression for the capacitance of our STO//DSO sample with IDEs. First, we adapt this expression to identify DSO capacitance and hence DSO polarization from data (Supplementary Note 8). We are then able to exploit the expression to obtain STO film capacitance and hence STO film polarization from data (Supplementary Note 9). Separately, we use the adapted expression to identify bulk STO capacitance and hence bulk STO polarization from data (Supplementary Note 10). Note that we will use C_{meas} to denote both expressions for measured capacitance, and measured capacitance data.

(1) Conformal mapping of a ferroelectric film with IDEs. Let us consider a hypothetical ferroelectric film that is addressed by an infinite array of infinitely long, infinitely thin IDEs of finite width. In order to appreciate conformal mapping to the parallel-plate geometry, let us assume a film thickness (h = 25 μm) that is comparable with our IDE dimensions (electrode width s = 25 μm, inter-electrode gap g = 50 μm). Using the real and imaginary parts of complex planes to represent horizontal and vertical real-space coordinates, respectively, we may transform a repeating unit of the sample cross-section in the complex y plane (Fig. S7a) to a parallel-plate capacitor geometry in the complex w plane (Fig. S7c) via a transitional geometry in the complex t plane (Fig. S7b). The first transformation is $t = \cosh^2\left(\frac{\pi z}{2h}\right)$. The second transformation is $w = \int_0^t [(t'-t_1)(t'-t_2)(t'-t_3)(t'-t_4)]^{-1/2}\mathrm{d}t'$, with mapping points 1-4 at $(t_{1\text{-}4},0)$ in the t plane, where $t_1 = 1$, $t_2 = \cosh^2\left(\frac{\pi s}{4h}\right) \sim 1.75$, $t_3 = \cosh^2\left[\frac{\pi(s+2g)}{4h}\right] \sim 644$ and $t_4 = \cosh^2\left[\frac{\pi(s+g)}{2h}\right] \sim 3098$. The integration is path independent as the integrand is differentiable, so the integration was performed along straight lines in the complex plane, but we verified for one specific case that the same result is achieved by varying first the real part of t' and then the imaginary part of t', or vice versa). In all three complex planes (y, t and w), one half electrode of width s/2 runs between mapping points 1 and 2, the other half electrode runs between mapping points 3 and 4.

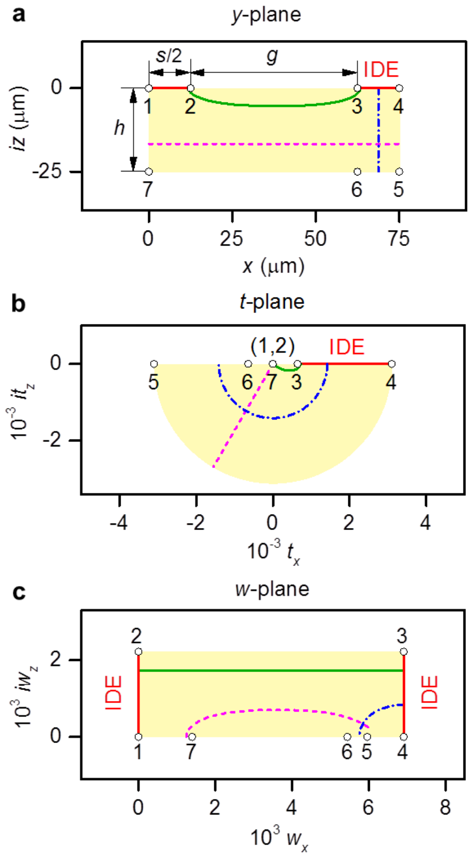

Figure S7. Conformal mapping of a ferroelectric film with IDEs. One repeating unit with IDEs of half width s/2 (red) on a ferroelectric film (yellow) in (a) the original y plane, (b) the transitional t plane, and (c) the final w plane. Straight pink dashed line and straight blue dot-dashed line in (a) necessarily continue to meet at normal incidence after transformation to the t and then w planes. Straight green lines of electric field in (c) necessarily continue to meet IDEs at normal incidence after transformation to the t and then y planes. Mapping points 1-7 are identified using unfilled circles. In (b), mapping points 1, 2 and 7 almost overlap.

(2) Contributions to the capacitance of a ferroelectric film measured with IDEs. Without recourse to formulae that we present in (3), we explain here how the measured capacitance C_{meas} for our hypothetical ferroelectric film with IDEs (Fig. S7a) involves the air on each side out to a distance of h_0 (Fig. S8a), which we let tend to infinity. The expression for C_{meas} is constructed by:

- adding the contribution for the air above the IDEs out to h_0 (this contribution is evaluated after transformation to the parallel-plate geometry, Fig. S8b);
- imagining air everywhere below the IDEs and adding the above contribution for a second time;
- subtracting the contribution of air in the space of thickness h_1 that is occupied by the ferroelectric (this contribution is evaluated after transformation to the parallel-plate geometry, Fig. S8c); and
- adding the contribution of the ferroelectric of thickness h_1 (this contribution is evaluated after transformation to the parallel-plate geometry, Fig. S8d).

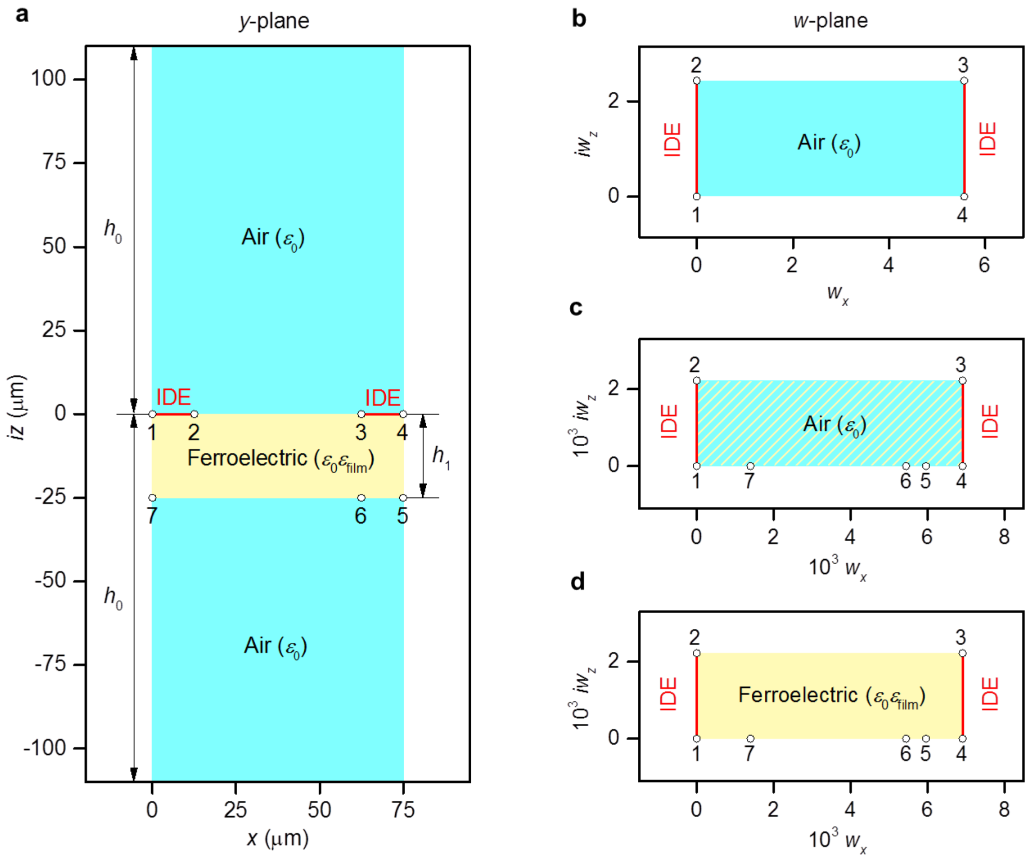

Figure S8. Contributions to the capacitance of a ferroelectric film measured with IDEs. (a) In the y plane, we show one repeating unit of IDEs (red) on a ferroelectric film with dielectric response ε_{film} (yellow). On each side of the film there is air whose permittivity we take as $\varepsilon_0 = 8.85\times10^{-12}$ F m^{-1} (light blue). Transformations to the w plane (cf. Fig. S7) are presented for (b) the air above the IDEs out to h_0, (c) a layer of air that occupies the space occupied by the ferroelectric film, and (d) the ferroelectric film alone. Mapping points 1-7 are identified using unfilled circles. We let $h_0 \to \infty$, such that the aspect ratio defining the air capacitor in (b) tends to $\frac{K(\sqrt{2}/10)}{K(7\sqrt{2}/10)} \sim 0.44$ [see first bullet point in Section (3)]. The value of $h_0 = 110$ μm rendered here achieves 99.12% of this aspect ratio.

(3) Expression for the measured capacitance of STO//DSO with IDEs. By employing the above process of adding and subtracting terms that are evaluated after transformation to the parallel-plate geometry, the measured capacitance C_{meas} of our STO (58 nm)//DSO (0.5 mm) sample with IDEs (Fig. S9) is given by:

$$C_{\mathrm{meas}} = \varepsilon_0 L \left\{ 2\frac{K(k_0)}{K(k_0')} - \frac{K(k_1)}{K(k_1')} + \varepsilon_{\mathrm{sub}} \left[\frac{K(k_1)}{K(k_1')} - \frac{K(k_2)}{K(k_2')} \right] + \varepsilon_{\mathrm{film}} \frac{K(k_2)}{K(k_2')} \right\},$$

where $\varepsilon_{\mathrm{sub}}$ is the dielectric response of the DSO substrate, $\varepsilon_{\mathrm{film}}$ is the dielectric response of the STO film, and $\varepsilon_0 = 8.85 \times 10^{-12}$ F m^{-1} approximates the permittivity of air. The function $K(x)$ denotes the elliptic integral of the first kind, whose argument is either:

$$k_i = \frac{\sinh\left(\frac{\pi s}{4h_i}\right)}{\sinh\left[\frac{\pi(s+2g)}{4h_i}\right]} \sqrt{\left\{\sinh^2\left[\frac{\pi(3s+2g)}{4h_i}\right] - \sinh^2\left[\frac{\pi(s+2g)}{4h_i}\right]\right\} \Big/ \left\{\sinh^2\left[\frac{\pi(3s+2g)}{4h_i}\right] - \sinh^2\left(\frac{\pi s}{4h_i}\right)\right\}}$$

or

$$k_i' = \sqrt{1 - k_i^2},$$

with $i = 0$, 1 or 2. Here, the air is assumed to extend on both sides out to $h_0 \to \infty$ (in which limit $k_0 \to \frac{s}{s+2g}\sqrt{\frac{2s}{2s+g}}$), the STO//DSO sample thickness is $h_1 = 0.5$ mm + 58 nm ~ 0.5 mm, and the STO film thickness is $h_2 = 58$ nm. The IDEs have electrode width $s = 25$ μm, and inter-electrode gap $g = 50$ μm. All 59 electrode pairs taken together have a total length of $L = 110$ mm.

In the above expression for C_{meas}:

- $2\frac{K(k_0)}{K(k_0')}$ represents air on both sides of the IDEs out to h_0 (the 2 is for the two sides) and $h_0 \to \infty$ implies $2\frac{K(k_0)}{K(k_0')} \to 2\frac{K(\sqrt{2}/10)}{K(7\sqrt{2}/10)} \sim 0.88$;
- $-\frac{K(k_1)}{K(k_1')}$ represents the subtraction for air in the space occupied by DSO//STO out to h_1;
- $\varepsilon_{\mathrm{sub}}\frac{K(k_1)}{K(k_1')}$ represents DSO in the space occupied by DSO//STO out to h_1;
- $-\varepsilon_{\mathrm{sub}}\frac{K(k_2)}{K(k_2')}$ represents the subtraction for DSO in the space occupied by STO out to h_2; and
- $\varepsilon_{\mathrm{film}}\frac{K(k_2)}{K(k_2')}$ represents STO out to h_2.

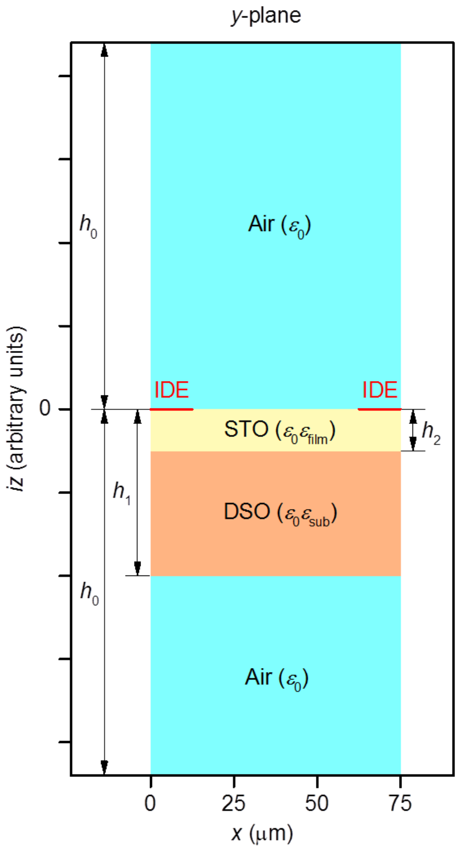

Figure S9. STO//DSO measured with IDEs. In the y plane, we show one repeating unit of IDEs (red) on the STO film (yellow) with dielectric response $\varepsilon_{\rm film}$. The DSO substrate (orange) has dielectric response $\varepsilon_{\rm sub}$. Each side of the STO//DSO sample has air (light blue) with approximate permittivity $\varepsilon_0 = 8.85 \times 10^{-12}$ F m^{-1}. Relative layer thicknesses ($h_0 \rightarrow \infty$, $h_1 \sim 0.5$ mm, $h_2 = 58$ nm) are not to scale.

Note 8. Permittivity and polarization of the DSO substrate

For a DSO substrate with IDEs (Fig. S10), we (1) use the conformal mapping developed in Supplementary Note 7 to convert measured capacitance $C_{meas}(T)$ to dielectric response $\varepsilon_{sub}(T)$ (Fig. S11a), and (2) use this dielectric response $\varepsilon_{sub}(T)$ to convert measured charge $Q_{meas}(T,E)$ to polarization $P_{sub}(T,E)$ (Fig. S11b).

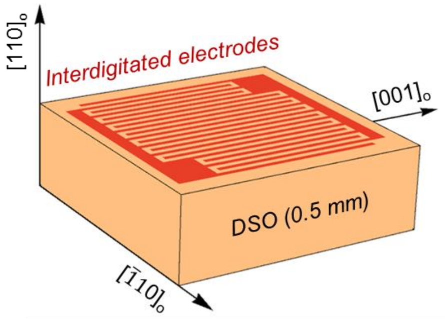

Figure S10. IDEs on a DSO substrate.

(1) $C_{meas}(T)$ for DSO → $\varepsilon_{sub}(T)$ for DSO. We use Supplementary Note 7 to identify $C_{meas}(T) = \varepsilon_0 L \left\{2\frac{K(k_0)}{K(k_0')} + [\varepsilon_{sub}(T) - 1]\frac{K(k_1)}{K(k_1')}\right\}$ for the DSO substrate without the STO film, and we have added temperature arguments to C_{meas} and ε_{sub}, which we assume to be field independent. We also assume that the geometrical parameters linking C_{meas} and ε_{sub} take the values specified in Supplementary Note 7 at all temperatures and fields, such that we ignore thermal expansion and electrostriction. Rearrangement yields $\varepsilon_{sub}(T) = 2.26C_{meas}(T)/(\varepsilon_0 L) - 1.00$, i.e. direct proportionality with a relatively small offset. For the DSO substrate, we may thus transform the measured capacitance $C_{meas}(T)$ into the dielectric response $\varepsilon_{sub}(T)$ (Fig. S11a). The room-temperature value of 20.9 matches well the value of ~21 from the data sheet of the vendor (CrysTec).

Given that $\varepsilon_{sub}(T) = 2.26C_{meas}(T)/(\varepsilon_0 L) - 1.00$ can only be directly evaluated for temperatures at which we have measured $C_{meas}(T)$, we perform a linear fit to obtain $\varepsilon_{sub}(T) = 19.87+1.07T/T_0$ (with $T_0 = 300$ K) in order to evaluate $\varepsilon_{sub}(T)$ at any temperature, as required in Supplementary Note 9.

(2) $Q_{meas}(T,E)$ for DSO → $P_{sub}(T,E)$ for DSO. This conversion is achieved by writing polarization $P_{sub}(T,E) = \varepsilon_0[\varepsilon_{sub}(T) - 1]E$ for the linear dielectric, and using $\varepsilon_{sub}(T) = 2.26C_{meas}(T)/(\varepsilon_0 L) - 1.00$ from above, where $C_{meas}(T) = Q_{meas}(T,E)/V = Q_{meas}(T,E)/(gE)$ given our ubiquitous assumption that $E = V/g$ represents the reasonably homogeneous electric field when voltage V is applied across IDEs with gap g. Substitution yields $P_{sub}(T,E) = 2.26Q_{meas}(T,E)/(gL) - 2.00\varepsilon_0 E$. We may thus transform $Q_{meas}(T,E)$ into $P_{sub}(T,E)$ for the DSO substrate (Fig. S11b). For DSO at 300 K, we have $|P_{sub}| \sim 0.038$ μC cm^{-2} at our highest field of $|E| = 20$ kV cm^{-1} (Fig. S11b), and a gradient dP_{sub}/dE that implies $\varepsilon_{sub} \sim 21$, in agreement with the dielectric data (Fig. S11a).

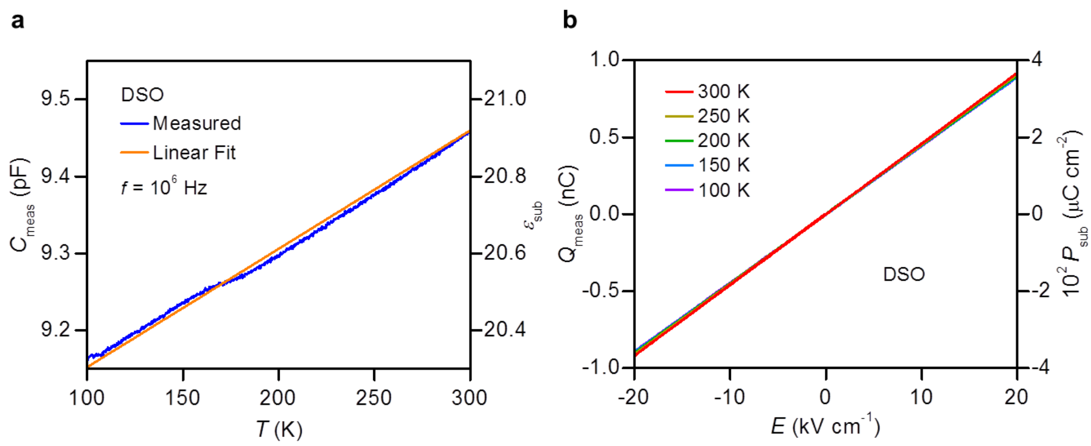

Figure S11. DSO substrate permittivity and polarization from capacitance and charge data measured with IDEs. (a) Zero-bias capacitance $C_{meas}(T)$ measured at 939 temperatures (left axis), and hence the dielectric response $\varepsilon_{sub}(T) = 2.26C_{meas}(T)/(\varepsilon_0 L) - 1.00$ (right axis). A linear fit yields $\varepsilon_{sub}(T) = 19.87+1.07T/T_0$ (orange line) for use in Supplementary Note 9. (b) At five of 636 measurement temperatures, we show measured charge Q_{meas} versus applied field $E \sim V/g$ (left axis), and hence polarization $P_{sub}(T,E) = 2.26Q_{meas}(T,E)/(gL) - 2.00\varepsilon_0 E$ (right axis). Data based on measurements of DSO substrate 1.

Note 9. Dielectric response and polarization of the STO film

Here we apply the conformal mapping developed in Supplementary Note 7 to convert electrical measurements of our STO//DSO sample with IDEs into data for the STO film. In (1), we convert measured capacitance C_{meas} to dielectric response $\varepsilon_{\text{film}}$. In (2), we convert measured charge Q_{meas} to polarization P_{film}. In both (1) and (2), similar results are obtained by subtracting from the measured data the corresponding data for a DSO substrate with equivalent IDEs (Fig. S10). This subtraction assumes a parallel-plate geometry between electrode pairs, a plate separation of g = 50 μm, and a plate area of h_2L (where h_2 = 58 nm is the STO film thickness, and L = 110 mm is the total length of all 59 electrode pairs).

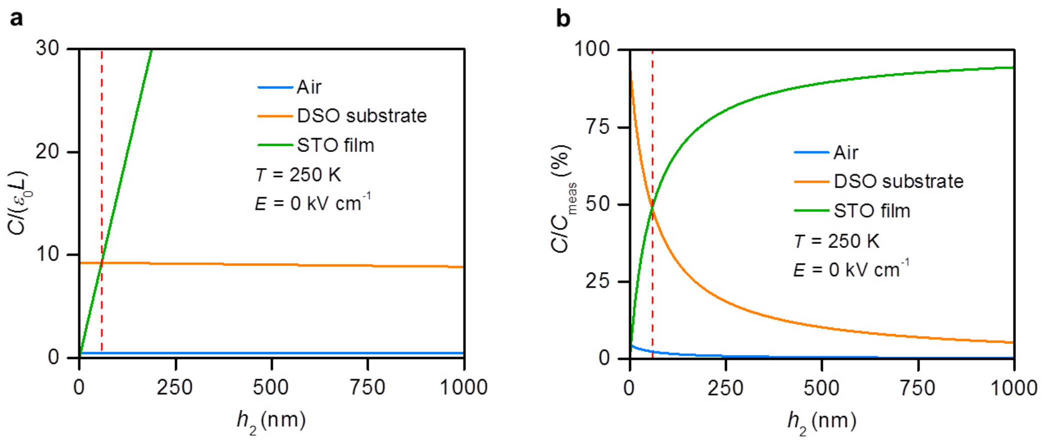

Figure S12. Contributions of air/DSO/STO to C_{meas} as a function of STO film thickness. (a) The capacitance C of each component in our STO//DSO sample exposed to air, measured with IDEs at 250 K and zero bias, versus STO film thickness h_2. (b) The percentage contribution of each such component to the measured capacitance C_{meas}. The value of C for the STO film is proportional to h_2 (58 nm for our film, red dashed line). Note that L = 110 mm is the total length of all 59 electrode pairs, ε_0 is the permittivity of free space, and there is air on both sides of the STO//DSO sample.

(1) $C_{\text{meas}}(T,E)$ for STO//DSO → $\varepsilon_{\text{film}}(T,E)$ for STO. As shown in Supplementary Note 7, the capacitance of our STO (58 nm)//DSO (0.5 mm) sample with IDEs (Fig. S9) is given by $C_{\text{meas}}(T,E) = \varepsilon_0 L\left\{2\frac{K(k_0)}{K(k_0')} - \frac{K(k_1)}{K(k_1')} + \varepsilon_{\text{sub}}(T)\left[\frac{K(k_1)}{K(k_1')} - \frac{K(k_2)}{K(k_2')}\right] + \varepsilon_{\text{film}}(T,E)\frac{K(k_2)}{K(k_2')}\right\}$, now with the appropriate temperature and field arguments (Fig. S12 shows the relative contributions of air/DSO/STO to C_{meas} as a function of film thickness h_2, at 250 K and zero bias). We assume that the geometrical parameters linking C_{meas} with ε_{sub} and $\varepsilon_{\text{film}}$ take the values specified in Supplementary Note 7 at all temperatures and fields, such that we ignore thermal expansion and electrostriction in the DSO substrate, and such that we ignore piezoelectricity and thermal expansion in the laterally clamped STO film. Substituting $\varepsilon_{\text{sub}}(T) = 19.87+1.07T/T_0$ for DSO from Supplementary Note 8 and rearranging yields $\varepsilon_{\text{film}}(T,E) = aC_{\text{meas}}(T,E)/(\varepsilon_0 L) + bT/T_0 + c$, with dimensionless parameters a = 863, b = –407 and c = –7938. Capacitance data $C_{\text{meas}}(T,E)$ for the STO//DSO sample may thus be converted into the dielectric response $\varepsilon_{\text{film}}(T,E)$ for the STO film:

- **Variable T, $E = 0$.** The capacitance $C_{meas}(T,0)$ measured for STO//DSO (red, Fig. S13a) yields the dielectric response $\varepsilon_{film}(T,0)$ for the STO film (green, Fig. S13a). If instead one subtracts $C_{meas}(T,0)$ for DSO (orange, Fig. S13a), and assumes the parallel-plate geometry described at the start of this Note, then one obtains similar results for $\varepsilon_{film}(T,0)$ (blue dashes, Fig. S13a). The corresponding data for $1/\varepsilon_{film}(T,0)$ (Fig. S13b) show good linearity with respect to temperature above the Curie temperature.
- **Variable E, $T = 200$ K.** The bipolar capacitance $C_{meas}(200\text{ K},E)$ measured for STO//DSO (red, Fig. S13c) yields $\varepsilon_{film}(200\text{ K},E)$ (green, Fig. S13c). If instead one subtracts bipolar $C_{meas}(200\text{ K},E)$ data measured for DSO (orange, Fig. S13c), and assumes the parallel-plate geometry described at the start of this Note, then one obtains similar results for $\varepsilon_{film}(200\text{ K},E)$ (blue dashes, Fig. S13c).

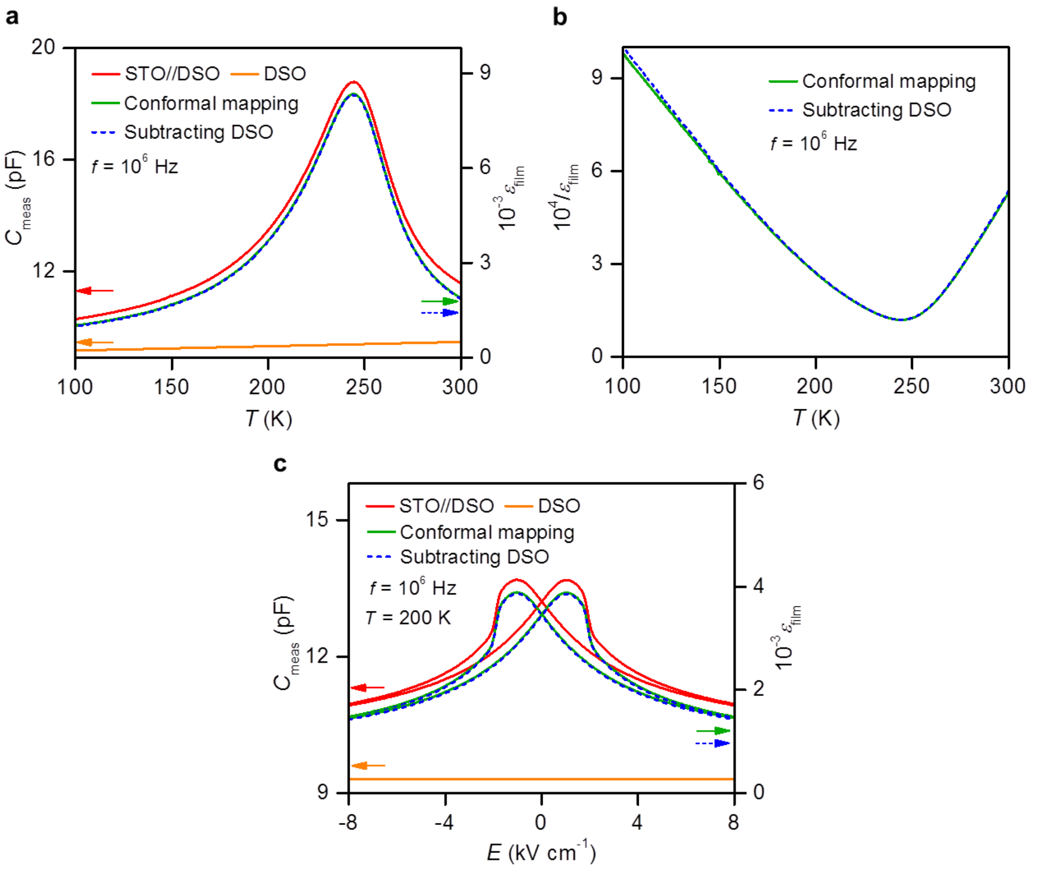

Figure S13. Dielectric response of the STO film from capacitance data for STO//DSO measured with IDEs. (a) Zero-bias capacitance $C_{meas}(T,0)$ measured for STO//DSO at 1614 temperatures (red), and hence the dielectric response $\varepsilon_{film}(T,0)$ of the STO film deduced either via conformal mapping (green), or via subtraction (blue dashes) of the corresponding data for a DSO substrate (orange). (b) Plots of $1/\varepsilon_{film}(T,0)$ corresponding to $\varepsilon_{film}(T,0)$ in (a). (c) Bipolar capacitance $C_{meas}(200\text{ K},E)$ with 200 data points measured for STO//DSO (red), and hence the dielectric response $\varepsilon_{film}(200\text{ K},E)$ of the STO film deduced either via conformal mapping (green), or via subtraction (blue dashes) of the corresponding data for a DSO substrate (orange). All data obtained at 10^6 Hz. Bias field E in (c) swept at ~1 Hz. Data based on measurements of Sample 1 and DSO substrate 1 (we use the DSO fit given in the caption of Fig. S11).

(2) $Q_{meas}(T,E)$ for STO//DSO → $P_{film}(T,E)$ for STO. This conversion is achieved by writing $\partial P_{film}(T,E)/\partial E = \varepsilon_0[\varepsilon_{film}(T,E) - 1]$ for the non-linear dielectric (ferroelectric) above (below) T_C, and using $\varepsilon_{film}(T,E) = aC_{meas}(T,E)/(\varepsilon_0 L) + bT/T_0 + c$ from (1), where $C_{meas}(T,E) = \partial Q_{meas}(T,E)/\partial V = \partial Q_{meas}(T,E)/\partial(gE)$ given our ubiquitous assumption that $E = V/g$ represents the reasonably homogeneous electric field when voltage V is applied across IDEs with gap g. Substitution and integration yield $P_{film}(T,E) = aQ_{meas}(T,E)/(gL) + (bT/T_0 + c - 1)\varepsilon_0 E = 863Q_{meas}(T,E)/(gL) - (407T/T_0 + 7938)\varepsilon_0 E$. Charge data $Q_{meas}(T,E)$ for the STO//DSO sample may thus be converted into polarization $P_{film}(T,E)$ for the STO film:

- **Variable *E*, *T* = 100 K.** The bipolar charge Q_{meas}(100 K,E) measured for STO//DSO (red, Fig. S14a) yields P_{film}(100 K,E) (green, Fig. S14a). If instead one subtracts bipolar Q_{meas}(100 K,E) data measured for DSO (orange, Fig. S14a), and assumes the parallel-plate geometry described at the start of this Note, then one obtains similar results for P_{film}(100 K,E) (blue dashes, Fig. S14a).
- **Variable *T*, *E* = 10 kV cm^{-1}.** The measured Q_{meas}(T,10 kV cm^{-1}) for STO//DSO (red, Fig. S14b) yields the polarization P_{film}(T,10 kV cm^{-1}) for the STO film (green, Fig. S14b). If instead one subtracts the temperature-dependent capacitance Q_{meas}(T,10 kV cm^{-1}) measured for DSO (orange, Fig. S14b), and assumes the parallel-plate geometry described at the start of this Note, then one obtains similar results for P_{film}(T,10 kV cm^{-1}) (blue dashes, Fig. S14b).

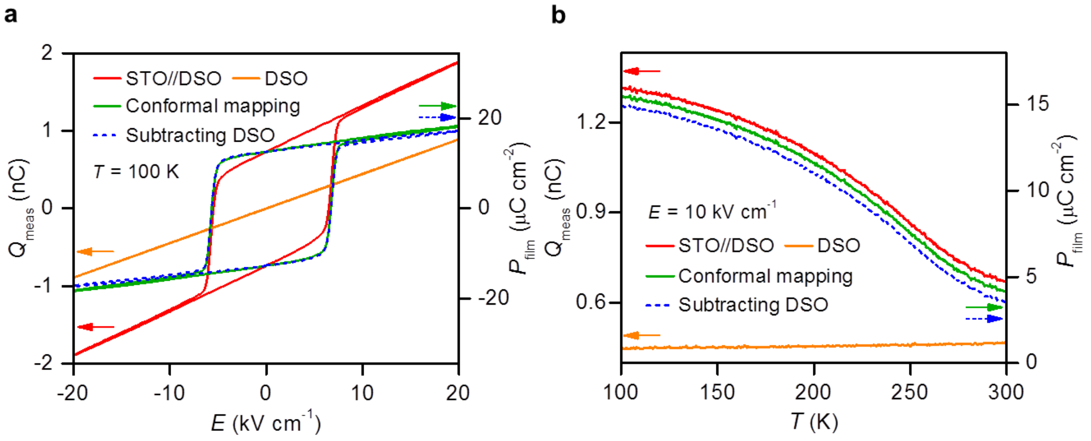

Figure S14. Polarization of the STO film from charge data for STO//DSO measured with IDEs. (a) Bipolar charge Q_{meas}(100 K,E) with 2001 data points measured for STO//DSO (red), and hence the polarization P_{film}(200 K,E) of the STO film deduced either via conformal mapping (green), or via subtraction (blue dashes) of a fit (not shown) to the corresponding data for a DSO substrate (orange). (b) Isofield charge Q_{meas}(T,10 kV cm^{-1}) measured at 668 temperatures for STO//DSO (red), and hence the polarization P_{film}(T,10 kV cm^{-1}) of the STO film deduced either via conformal mapping (green), or via subtraction (blue dashes) of a fit (not shown) to the corresponding data for a DSO substrate measured at 636 temperatures (orange). Data obtained while sweeping bias field E at 10^2 Hz. Data based on measurements of Sample 1 and DSO substrate 1.

Note 10. Ferroelectric, EC and dielectric behaviour of bulk STO

Electrical measurements for a single sample of bulk STO, i.e. an STO substrate, were obtained along a [100] direction using IDEs that are equivalent to those we used for STO//DSO (Supplementary Note 2). In (1), we use the conformal mapping developed in Supplementary Note 8 to convert measured capacitance $C_{meas}(T,E)$ to dielectric response $\varepsilon_{bulk}(T,E)$ (Fig. S15). In (2), we use this dielectric response $\varepsilon_{bulk}(T,E)$ to convert measured charge $Q_{meas}(T,E)$ to polarization $P_{bulk}(T,E)$ (Fig. S16). In (3), we use the conversion in (2) to present a full set of electrical polarization data, and hence evaluate EC effects. In (4), we use the conversion in (1) to present a full set of dielectric data, which we integrate to obtain electrical polarization data, and hence EC data.

(1) $C_{meas}(T,E)$ for bulk STO → $\varepsilon_{bulk}(T,E)$ for bulk STO. Our bulk STO with IDEs has the same geometry as our DSO substrate with IDEs. Therefore the relationship between C_{meas} and ε_{sub} for the DSO substrate [part (1) of Supplementary Note 8] can be used for bulk STO after replacing ε_{sub} with ε_{bulk}, giving $C_{meas}(T,E) = \varepsilon_0 L\left\{2\frac{K(k_0)}{K(k_0')} + \left[\varepsilon_{bulk}(T,E) - 1\right]\frac{K(k_1)}{K(k_1')}\right\}$. Here, as with DSO, we assume that the geometrical parameters linking C_{meas} and ε_{bulk} take the values specified in Supplementary Note 7 at all temperatures and fields, such that we ignore thermal expansion and electrostriction. However, unlike the expression for DSO, we have introduced the field argument, as required (Fig. S15c). Rearrangement yields $\varepsilon_{bulk}(T,E) = 2.26C_{meas}(T,E)/(\varepsilon_0 L) - 1.00$, cf. $\varepsilon_{sub}(T) = 2.26C_{meas}(T)/(\varepsilon_0 L) - 1.00$ for the DSO substrate. For bulk STO, capacitance data $C_{meas}(T,E)$ may thus be converted into the dielectric response $\varepsilon_{bulk}(T,E)$:

- **Variable *T*, *E* = 0.** The measured $C_{meas}(T,0)$ (red, Fig. S15a) yields the dielectric response $\varepsilon_{bulk}(T,0)$ (green, Fig. S15a). If instead $C_{meas}(T,0)$ is scaled by a factor of 396 to achieve $\varepsilon_{bulk}(300\text{ K},0) = 310$ (data for 10^6 Hz in ref. [S10]) then one obtains $\varepsilon_{bulk}(T,0)$ (blue dashes, Fig. S15a) (this scaling implies a thickness of $h_2 = 17$ μm in the parallel-plate geometry that we describe at the end of paragraph one in Supplementary Note 9). The corresponding data for $1/\varepsilon_{bulk}(T,0)$ (Fig. S15b) show good linearity with respect to temperature.
- **Variable *E*, *T* = 100 K.** The measured bipolar capacitance $C_{meas}(100\text{ K},E)$ (red, Fig. S15c) yields $\varepsilon_{bulk}(100\text{ K},E)$ (green, Fig. S15c). If instead $C_{meas}(100\text{ K},E)$ is scaled by the aforementioned factor of 396 then one obtains similar results for $\varepsilon_{bulk}(100\text{ K},E)$ (blue dashes, Fig. S15c).

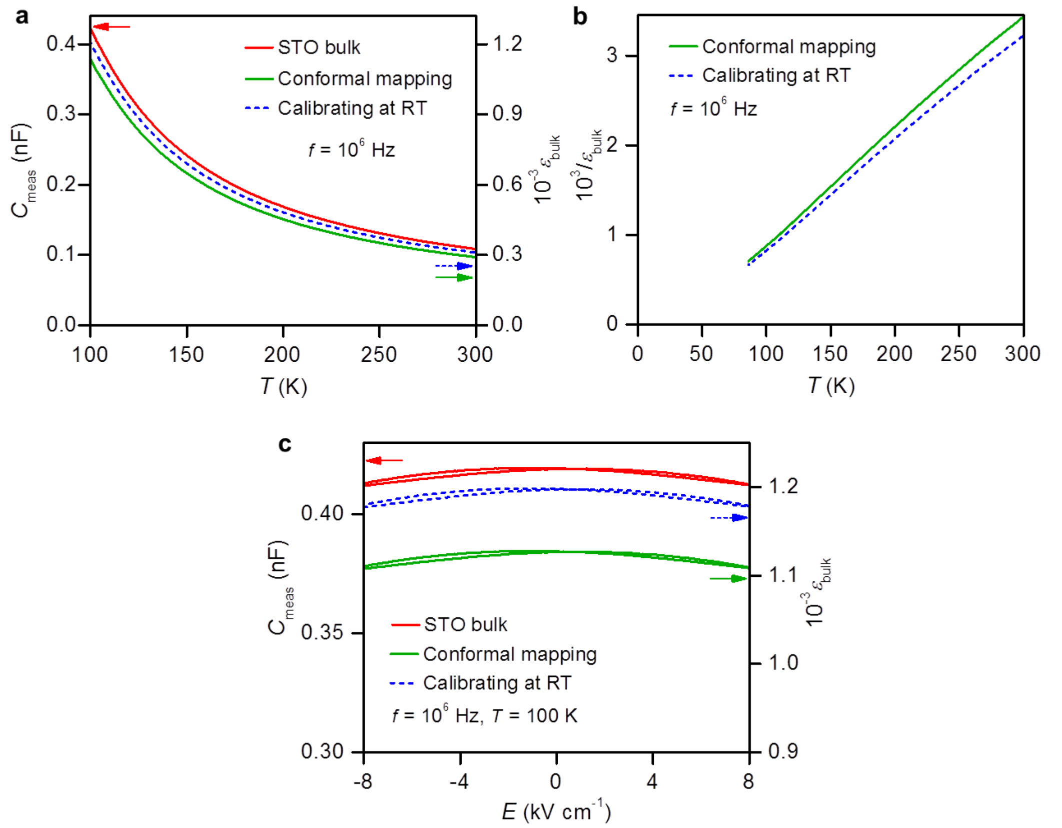

Figure S15. Dielectric response of bulk STO from capacitance data measured with IDEs. (a) Zero-bias capacitance $C_{meas}(T,0)$ measured at 378 temperatures (red), and hence the dielectric response $\varepsilon_{bulk}(T,0)$ deduced either via conformal mapping (green), or by scaling with a factor of 396 to achieve $\varepsilon_{bulk}(300\text{ K},0) = 310$ (data for 10^6 Hz in ref. [S10]) (blue dashes). (b) Plots of $1/\varepsilon_{bulk}(T,0)$ corresponding to $\varepsilon_{bulk}(T,0)$ in (a). The finite-temperature intercepts have been seen elsewhere[S11]. (c) Measured bipolar capacitance $C_{meas}(100\text{ K},E)$ with 200 data points (red), and hence the dielectric response $\varepsilon_{bulk}(100\text{ K},E)$ deduced either via conformal mapping (green), or by scaling with the aforementioned factor of 396. All data obtained at 10^6 Hz. Bias field E in (c) swept at ~1 Hz. Data based on measurements of our single sample of bulk STO.

(2) $Q_{meas}(T,E)$ for bulk STO → $P_{bulk}(T,E)$ for bulk STO. Our bulk STO with IDEs has the same geometry as our DSO substrate with IDEs. Therefore the relationship between Q_{meas} and P_{bulk} for the DSO substrate with IDEs [part (2) of Supplementary Note 8] can be used for bulk STO with IDEs after replacing P_{sub} with P_{bulk}. Hence $P_{bulk}(T,E) = 2.26Q_{meas}(T,E)/(gL)–2.00\varepsilon_0 E$. Charge data $Q_{meas}(T,E)$ may thus be converted into polarization $P_{bulk}(T,E)$:

- **Variable E, T = 100 K.** The measured bipolar charge $Q_{meas}(100\text{ K},E)$ (red, Fig. S16a) yields $P_{bulk}(100\text{ K},E)$ (green, Fig. S16a). If instead $Q_{meas}(100\text{ K},E)$ is scaled by a factor of 396 to achieve a gradient that corresponds to $\varepsilon_{bulk}(300\text{ K},0) = 310$ (data for 10^6 Hz in ref. [S10], ε_{bulk} assumed independent of field and frequency) then one obtains similar results for $P_{bulk}(100\text{ K},E)$ (blue dashes, Fig. S16a).
- **Variable T, E = 10 kV cm^{-1}.** The measured $Q_{meas}(T,10\text{ kV cm}^{-1})$ (red, Fig. S16b) yields $P_{bulk}(T,10\text{ kV cm}^{-1})$ (green, Fig. S16b). If instead $Q_{meas}(T,10\text{ kV cm}^{-1})$ is scaled by the aforementioned factor of 396 then one obtains similar results for $P_{bulk}(T,10\text{ kV cm}^{-1})$ (blue dashes, Fig. S16b).

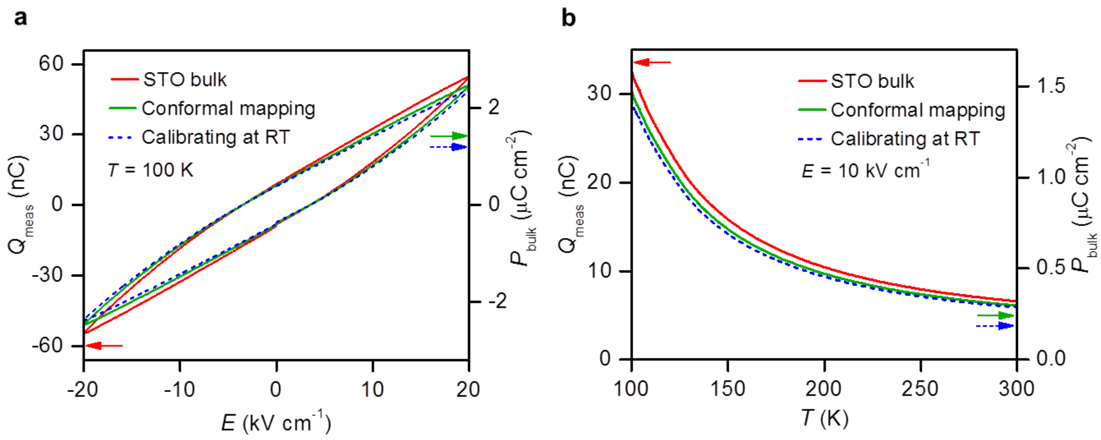

Figure S16. Polarization of bulk STO from charge data measured with IDEs. (a) Bipolar charge $Q_{\rm meas}(100\ {\rm K},E)$ with 2001 data points (red), and hence $P_{\rm bulk}(100\ {\rm K},E)$ deduced either via conformal mapping (green), or by scaling with a factor of 396 to achieve a gradient that corresponds to $\varepsilon_{\rm bulk}(300\ {\rm K},0) = 310$ (data for 10^6 Hz in ref. [S10], $\varepsilon_{\rm bulk}$ assumed independent of field and frequency, hysteresis as expected[S12]) (blue dashes). (b) Isofield charge $Q_{\rm meas}(T,10\ {\rm kV\ cm^{-1}})$ measured at 819 temperatures (red), and hence $P_{\rm bulk}(T,\ 10\ {\rm kV\ cm^{-1}})$ deduced either via conformal mapping (green), or by scaling with the aforementioned factor of 396. Data obtained while sweeping bias field E at 10^2 Hz. Data based on measurements of our single sample of bulk STO.

(3) Ferroelectric and EC measurements for bulk STO. Here we use the conversion in (2), i.e. $P_{\rm bulk}(T,E) = 2.26Q_{\rm meas}(T,E)/(gL)–2.00\varepsilon_0 E$, to present a full set of electrical polarization data (Fig. S17), which are used to evaluate EC effects (Fig. S18). For our maximum field of 20 kV cm^{-1}, we obtained EC effects of $|\Delta S_{\rm bulk}| = 0.53$ kJ K^{-1} m^{-3} and $|\Delta T_{\rm bulk}| = 41$ mK at 100 K, and $|\Delta S_{\rm bulk}| = 0.03$ kJ K^{-1} m^{-3} and $|\Delta T_{\rm bulk}| = 3$ mK at ~250 K. These EC effects in bulk STO are much smaller than the EC effects in our strained STO film, i.e. $|\Delta S_{\rm film}| = 1.8$ kJ K^{-1} m^{-3} (Fig. 6a,b in the main paper) and $|\Delta T_{\rm film}| = 0.17$ K (Fig. S29a,b).

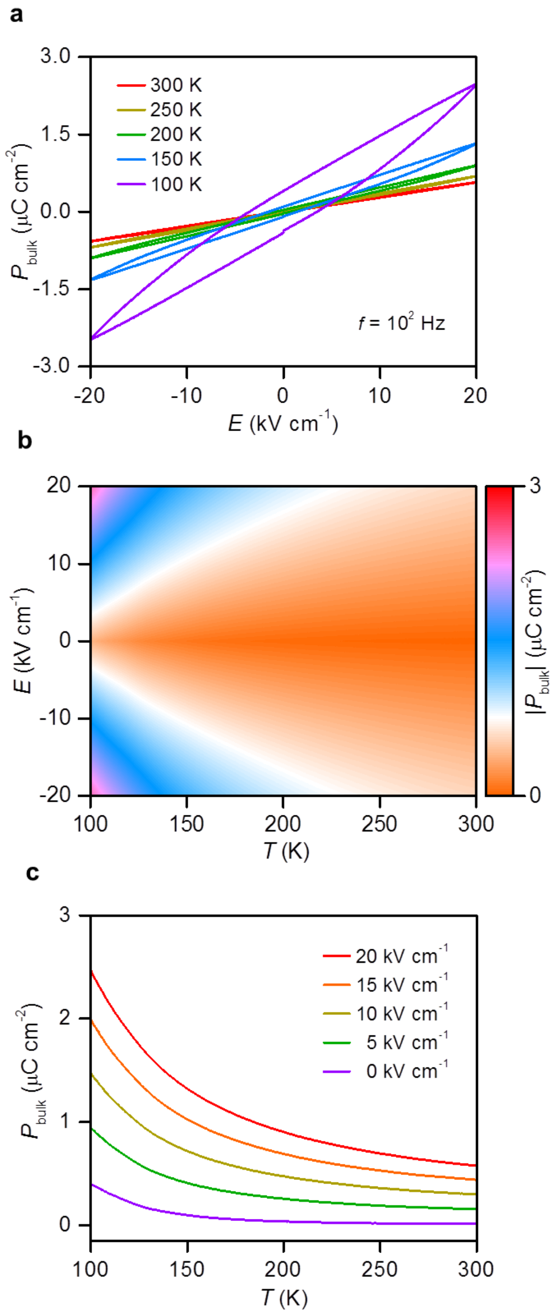

Figure S17. Electrical polarization of bulk STO. (a) Five of 819 bipolar $P_{bulk}(E)$ plots with 2001 data points and low-temperature hysteresis[S12]. (b) $|P_{bulk}(T,E)|$ from field-removal branches of all 819 bipolar $P_{bulk}(E)$ plots. (c) Isofield $P_{bulk}(T)$ transects through (b) at zero field and selected positive fields. Data based on measurements of our single sample of bulk STO.

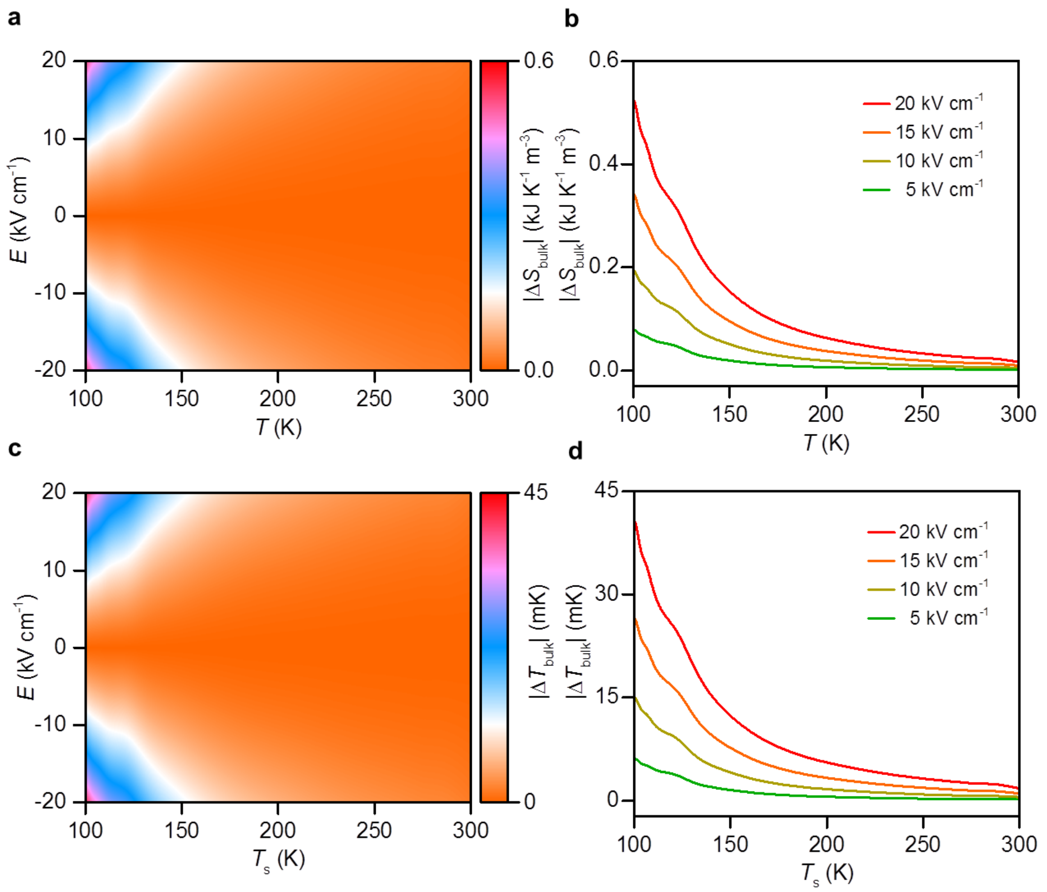

Figure S18. EC effects in bulk STO. (a) $|\Delta S_{\mathrm{bulk}}(T,E)| = |\int_0^E (\partial P_{\mathrm{bulk}}/\partial T)_{E'}\mathrm{d}E'|$ derived from $|P_{\mathrm{bulk}}(T,E)|$ data in Fig. S17b. (b) Isofield $|\Delta S_{\mathrm{bulk}}(T)|$ transects through (a) at selected positive fields. (c) $|\Delta T_{\mathrm{bulk}}(T_s,E)| \sim T_s|\Delta S_{\mathrm{bulk}}(T_s,E)|/c_{\mathrm{bulk}}(T_s)$ derived from both (a) and the volume-normalized heat capacity $c_{\mathrm{bulk}}(T_s)$ (Fig. S30 and caption), where T_s denotes starting temperature. (d) Isofield $|\Delta T_{\mathrm{bulk}}(T_s)|$ transects through (c) at selected positive fields. Data based on measurements of our single sample of bulk STO.

(4) EC effects in bulk STO via polarization from integrated dielectric data. Here we use the conversion in (1), i.e. $\varepsilon_{\mathrm{bulk}}(T,E) = 2.26C_{\mathrm{meas}}(T,E)/(\varepsilon_0 L) - 1.00$, to present a full set of dielectric measurements (Fig. S19) at different values of frequency, temperature and bias field, with a maximum bias field of 8 kV cm^{-1} (the corresponding measurements for our film are shown as Fig. 4 in the main paper).

We then integrate dielectric data obtained while reducing bias-field magnitude ($E < 0$ in Fig. S19c, $E > 0$ in Fig. S19d) to obtain polarization data (Fig. S20a-c) that match well with the corresponding measurements of polarization (Fig. S17), which we present in Fig. S20d-f out to 8 kV cm^{-1} rather than 20 kV cm^{-1}.

As a consequence of this match, both polarization datasets yield similar EC data out to 8 kV cm^{-1} ($|\Delta S_{\mathrm{bulk}}|$ in Fig. S21, $|\Delta T_{\mathrm{bulk}}|$ in Fig. S22).

Note that the integration of dielectric data requires a constant of integration that we take here to be $P_{\mathrm{bulk}}(T,0)$, so it does not represent a truly independent method of determining polarization.

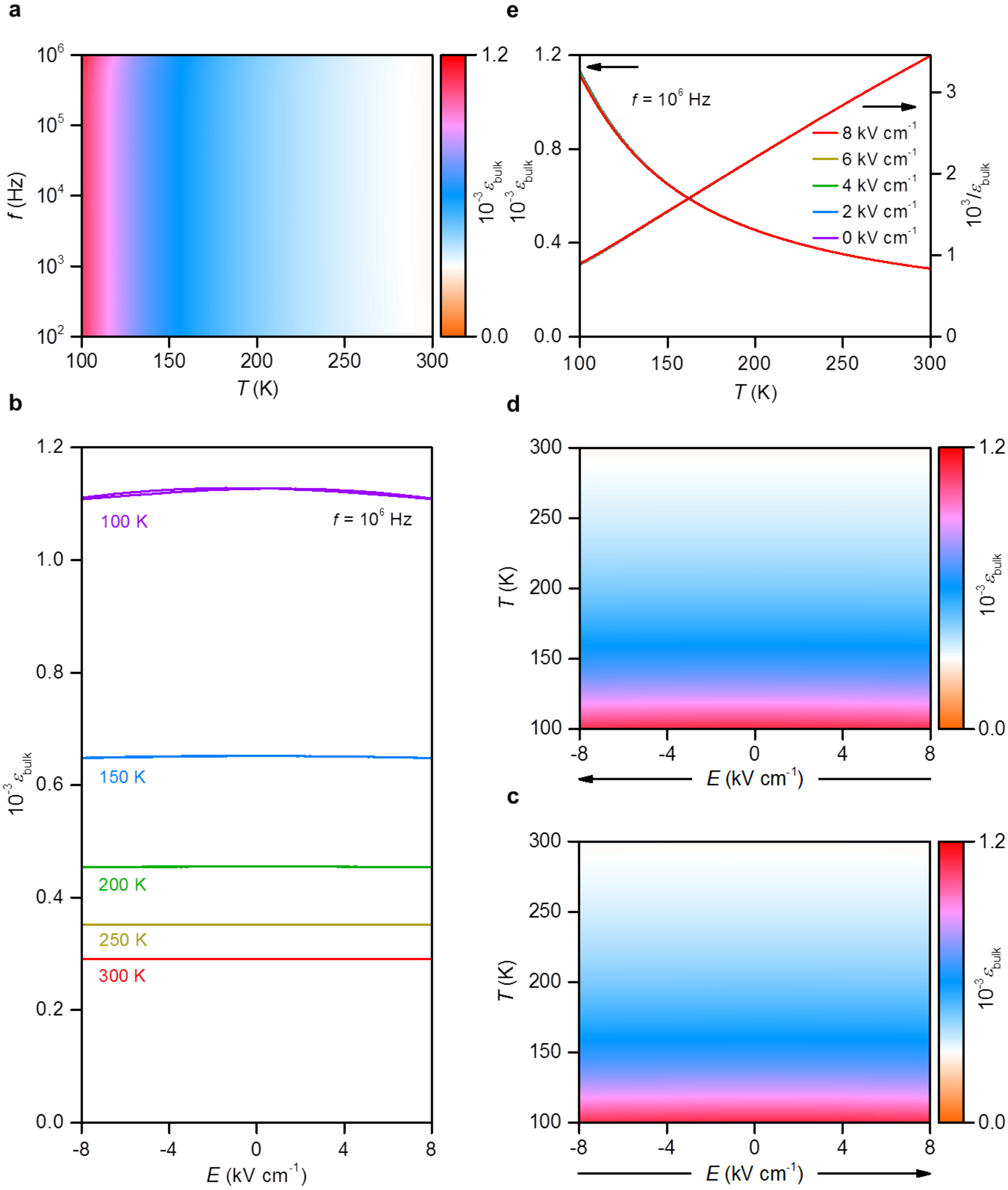

Figure S19. In-plane dielectric response of bulk STO. (a) Dielectric response $\varepsilon_{bulk}(T,f)$ based on isothermal $\varepsilon_{bulk}(f)$ plots measured in zero bias field at 378 values of temperature T and 200 values of frequency f. Panels (b-e) are constructed from isothermal $\varepsilon_{bulk}(E)$ with 200 data points, obtained at 10^6 Hz while sweeping bias field E at $f \sim 1$ Hz. (b) Bipolar $\varepsilon_{bulk}(E)$ at five of 1683 measurement temperatures. (c,d) $\varepsilon_{bulk}(T,E)$ constructed from bipolar $\varepsilon_{bulk}(E)$ at all 1683 measurement temperatures for (c) up and (d) down sweeps of field. (e) Isofield $\varepsilon_{bulk}(T)$ transects through (d) at postive bias fields (left axis) and the corresponding plots of $1/\varepsilon_{bulk}(T)$ (right axis) vary little with bias field, and can be well fitted to the Curie-Weiss law $\varepsilon_{bulk}(T) = M/(T - \theta)$ with $M = 7.8\times10^4$ K and $\theta = 31$ K (dashed grey arrow), similar to previous reports[S11]. Data based on measurements of our single sample of bulk STO.

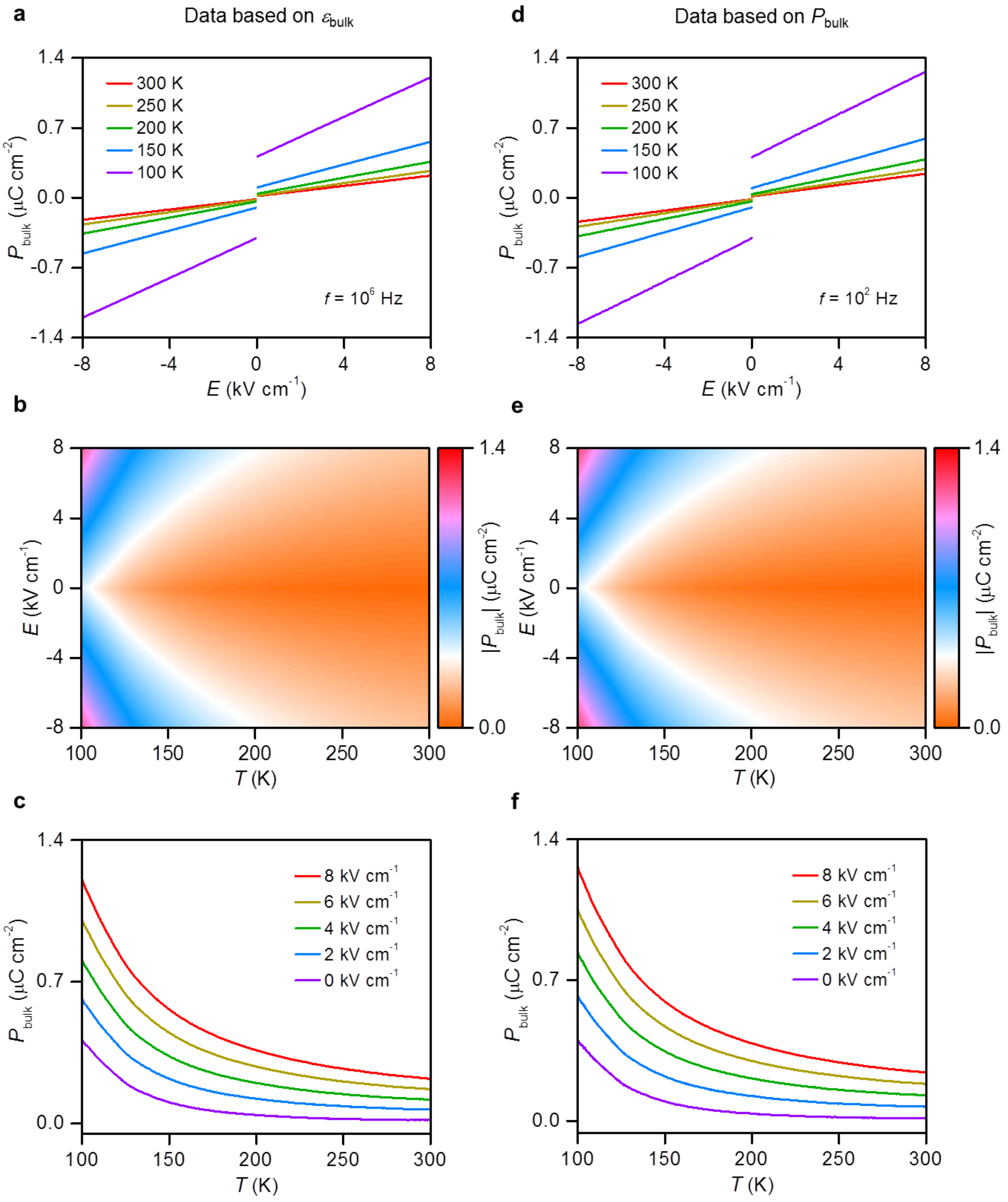

Figure S20. Electrical polarization of bulk STO via integration of dielectric data and via direct measurements. (a-c) Polarization $P_{\text{bulk}}(T,E) = \int_0^E \varepsilon_0\left[\varepsilon_{\text{bulk}}(T,E') - 1\right]\text{d}E' + P_{\text{bulk}}(T,0)$ from isothermal measurements of dielectric response $\varepsilon_{\text{bulk}}(T,E)$ while reducing bias-field magnitude ($E < 0$ in Fig. S19c, $E > 0$ in Fig. S19d) (ε_0 is the permittivity of free space). Integration constant $P_{\text{bulk}}(T,0)$ was identified at all 421 dielectric measurement temperatures by interpolation of remanent bipolar $P_{\text{bulk}}(E)$ values that were evaluated at 819 temperatures (purple plot, Fig. S17c). (a) $P_{\text{bulk}}(E)$ at five measurement temperatures. (b) $|P_{\text{bulk}}(T,E)|$ at all 421 dielectric measurement temperatures. (c) Isofield $P_{\text{bulk}}(T)$ transects through (b) at zero field and selected positive fields. (d-e) Polarization data out to 8 kV cm^{-1} based on field-removal branches of 819 bipolar $P_{\text{bulk}}(E)$ measurements out to 20 kV cm^{-1} (Fig. S17). (d) $P_{\text{bulk}}(E)$ at five measurement temperatures (subset of data in Fig. S17a). (e) $|P_{\text{bulk}}(T,E)|$ at all 819 measurement temperatures (subset of data in Fig. S17b). (f) Isofield $P_{\text{bulk}}(T)$ transects through (e) at zero field and selected positive fields. Data based on measurements of our single sample of bulk STO.

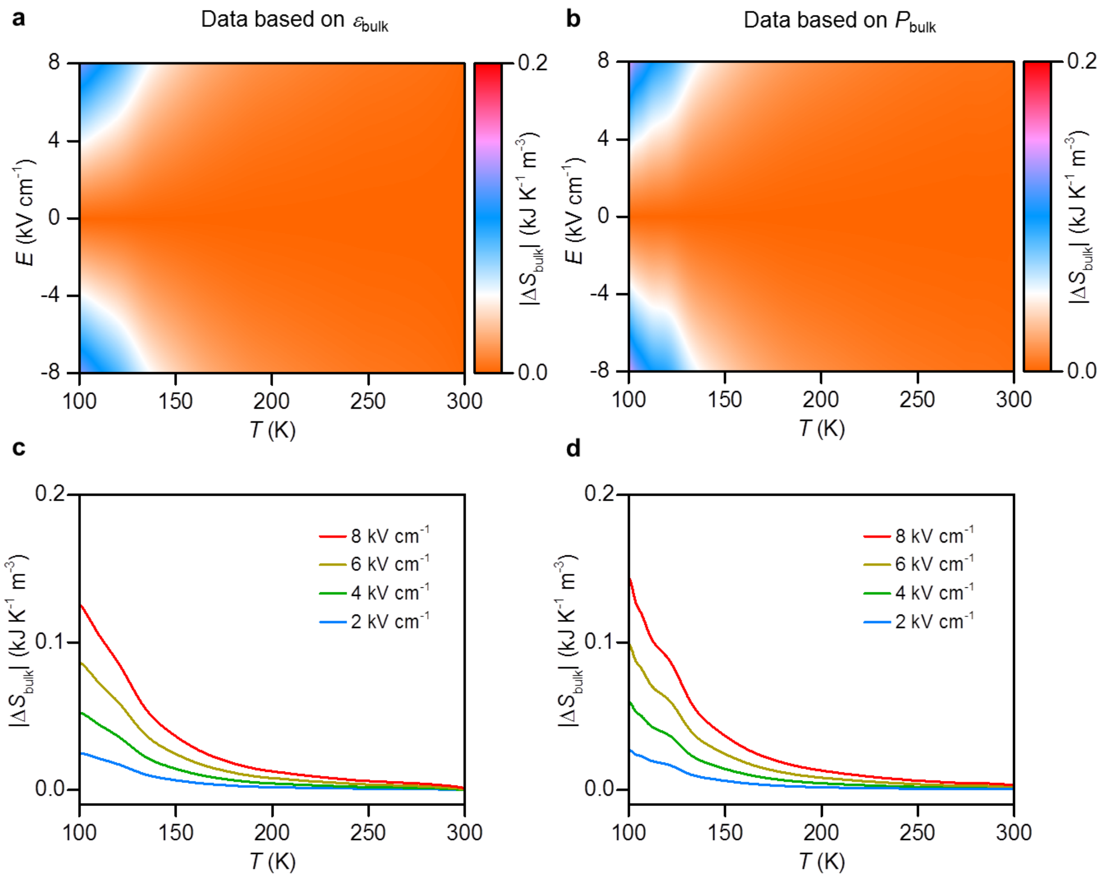

Figure S21. Isothermal EC effects in bulk STO deduced from integration of dielectric data and measurements of polarization. (a,b) $|\Delta S_{\mathrm{bulk}}(T,E)| = |\int_0^E (\partial P_{\mathrm{bulk}}/\partial T)_{E'} \mathrm{d}E'|$ derived from $|P_{\mathrm{bulk}}(T,E)|$ data obtained (a) by integrating dielectric data (Fig. S20b) and (b) by direct measurement (Fig. S20e). (c,d) Isofield $|\Delta S_{\mathrm{bulk}}(T)|$ transects through (a,b), respectively, at selected positive fields. Data based on measurements of our single sample of bulk STO.

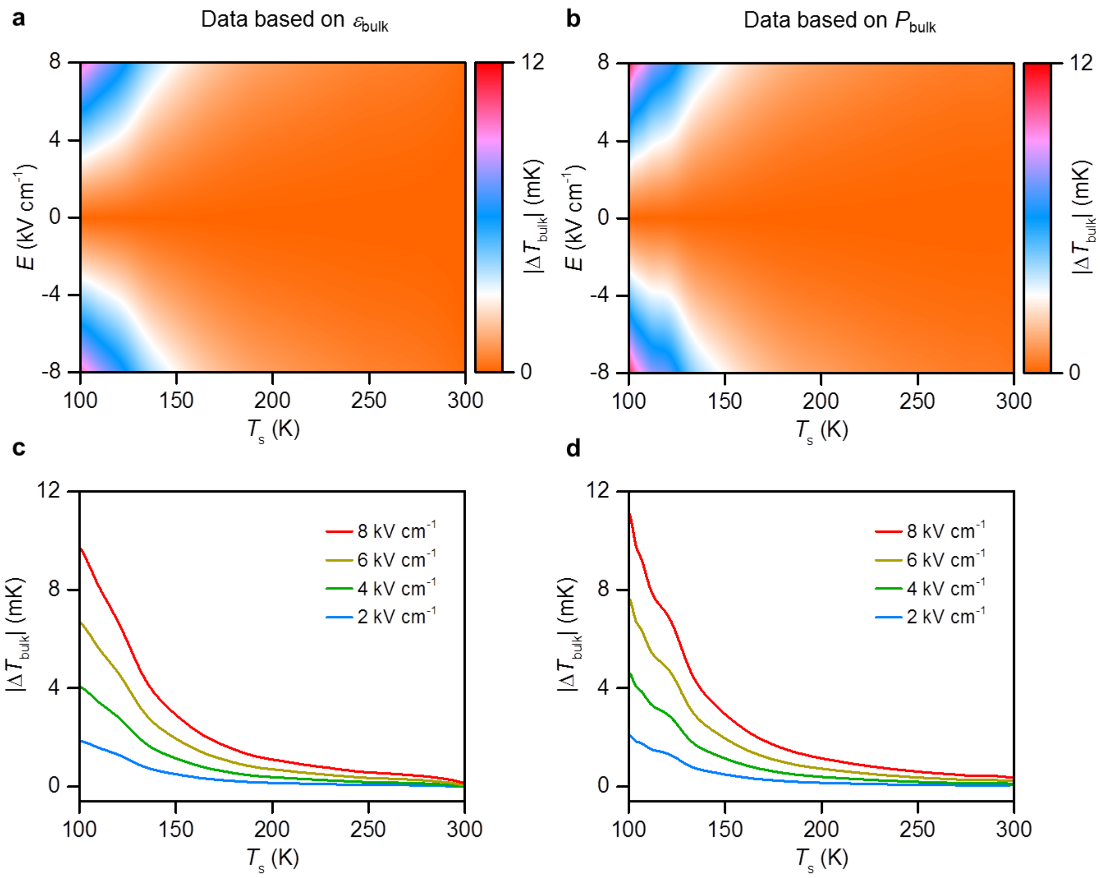

Figure S22. Adiabatic EC effects in bulk STO deduced from integration of dielectric data and measurements of polarization. (a,b) $|\Delta T_{bulk}(T_s,E)| \sim T_s|\Delta S_{bulk}(T_s,E)|/c_{bulk}(T_s)$ derived from $|\Delta S_{bulk}(T,E)|$ and the volume-normalized heat capacity $c_{bulk}(T_s)$ (Fig. S30 and caption), where T_s denotes starting temperature. In (a), we used $|\Delta S_{bulk}(T,E)|$ (Fig. S21a) based on $|P_{bulk}(T,E)|$ (Fig. S20b) obtained via integration dielectric data. In (b), we used $|\Delta S_{bulk}(T,E)|$ (Fig. S21b) based on $|P_{bulk}(T,E)|$ (Fig. S20e) obtained from direct measurements of polarization. (c,d) Isofield $|\Delta T_{bulk}(T_s)|$ transects through (a,b), respectively, at selected positive fields. Data based on measurements of our single sample of bulk STO.

Note 11. Dielectric response and loss tangent versus DC bias for STO//DSO

The relative dielectric permittivity $\varepsilon_0^{-1}\mathrm{d}D_{\mathrm{film}}/\mathrm{d}E$ of the STO film is complex, but the imaginary part (not shown) is much smaller than the real part, which we refer to everywhere as the dielectric response $\varepsilon_{\mathrm{film}}(T,E)$. Here we present dielectric data (Fig. S23) and loss tangent tan δ data (Figs S23-24). The loss tangent data are evaluated by the impedance analyzer when it measures permittivity, and they represent the film alone due to the approximate cancellation of geometrical factors and the DSO background when taking the ratio of imaginary and real parts.

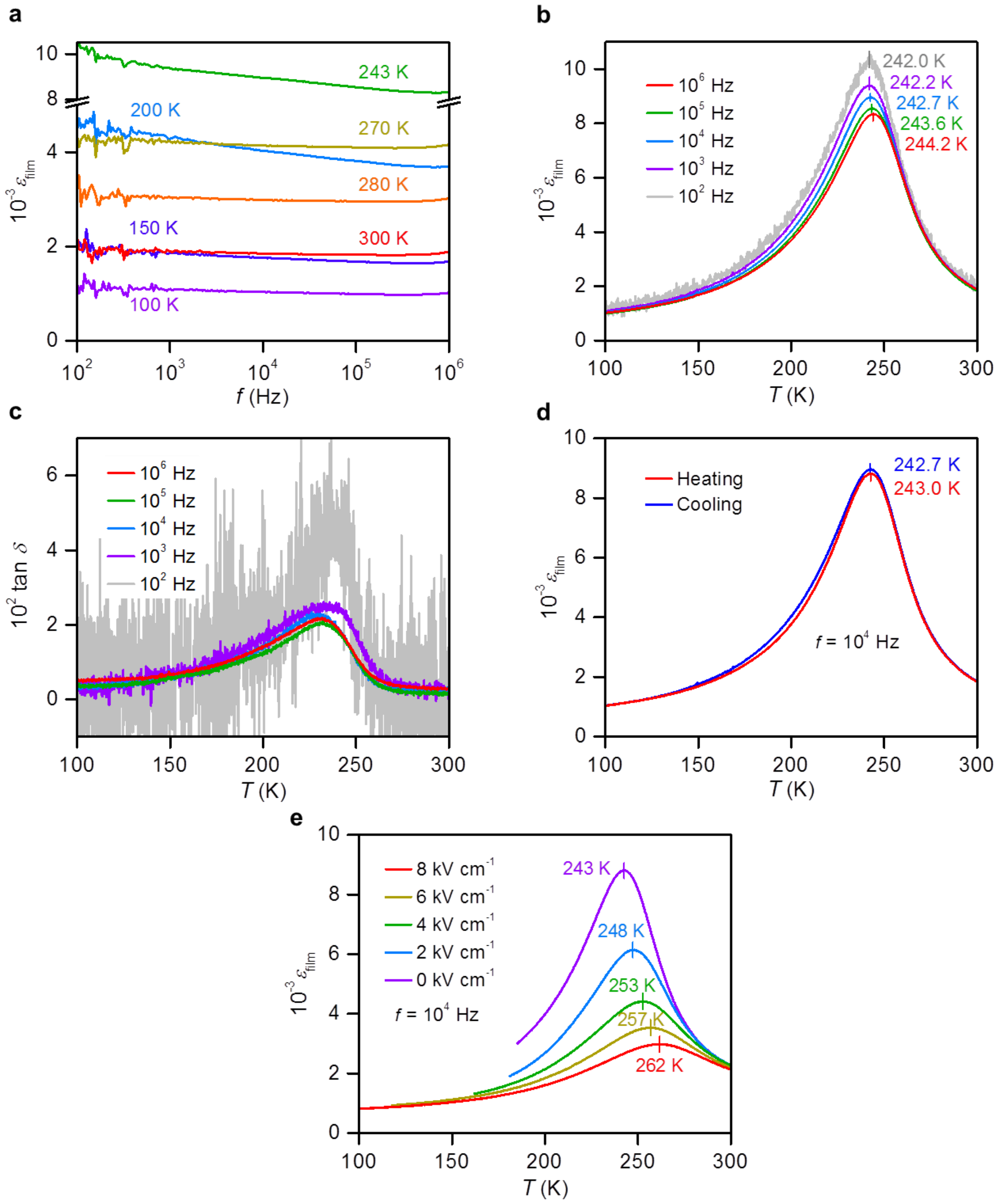

Figure S23. In-plane dielectric data for strained STO. (a) Isothermal and (b) isofrequency transects through $\varepsilon_{\mathrm{film}}(T,f)$ (Fig. 4a in the main paper) measured on zero-field cooling. (c) Loss tangent plots corresponding to the plots in (b). Low-frequency data in (a-c) are noisy. (d) $\varepsilon_{\mathrm{film}}(T)$ at 10^4 Hz on heating (red) and cooling (blue). The small discrepancy at intermediate temperatures is reproducible. (e) $\varepsilon_{\mathrm{film}}(T)$ on heating at selected values of $E \geq 0$. At $E = 0$, the somewhat exact coincidence of the peak with our chosen value of $T_C \sim 243$ K is circumstantial given discrepancies in measurement frequency and temperature-sweep direction. Data in (c) for Sample 1. Data in (a-b, d-e) based on measurements of Sample 1 and DSO substrate 1.

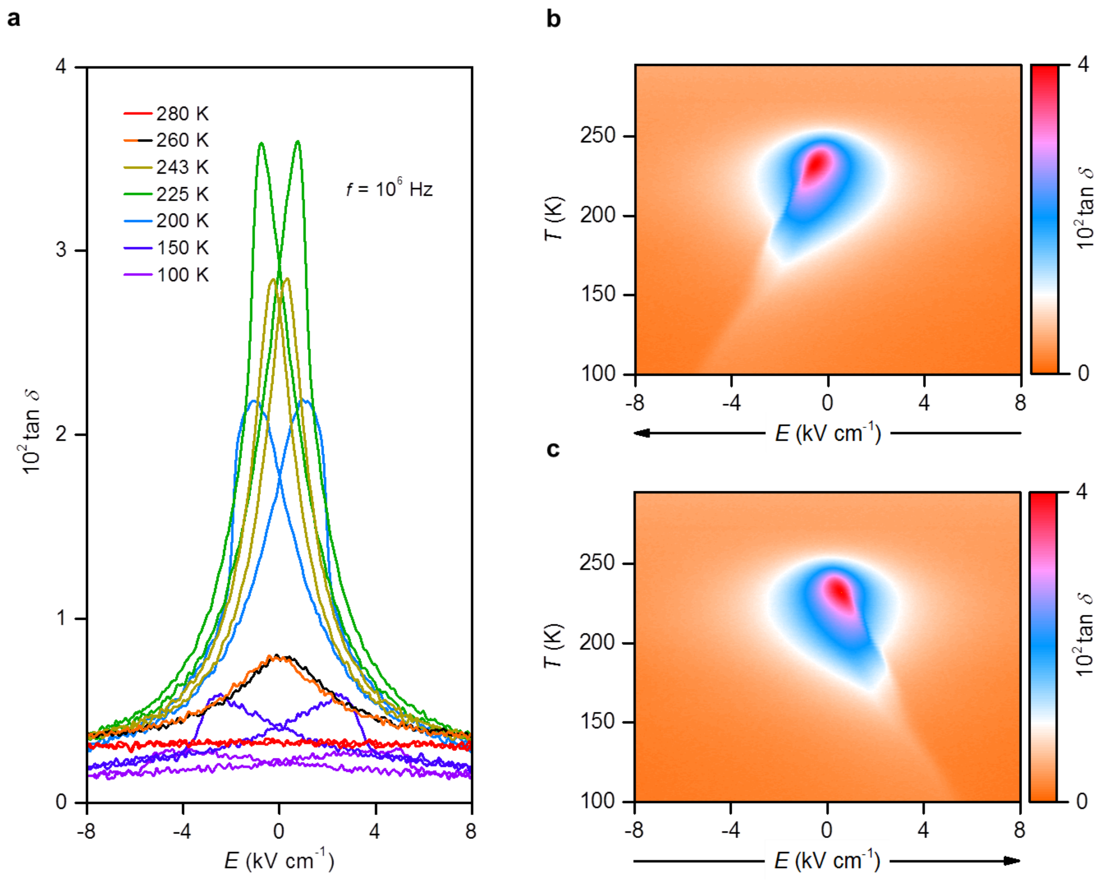

Figure S24. Loss tangent versus DC bias. Panels (a-c) show loss tangent data that correspond to the dielectric response $\varepsilon_{\mathrm{film}}(T,f)$ in Fig. 4c-e of the main paper (data near 300 K are absent here due to corruption by auto-ranging). Data for Sample 1.

Note 12. Bipolar and unipolar polarization in STO//DSO

Electrical polarization data (e.g. at 100 K, Fig. S25a) were acquired using a double-wave electric field[S13] (Fig. S25b) to construct bipolar and unipolar $P_{film}(E)$ plots such as those shown in Fig. S25c (which represents a subset of the data in Fig. 5 of the main paper). The unipolar plots show negligible hysteresis, and match well the outer branches of the corresponding bipolar plots, which are thus highly reversible. The bipolar plots contain 1000 data points, and the unipolar plots contain 500 data points.

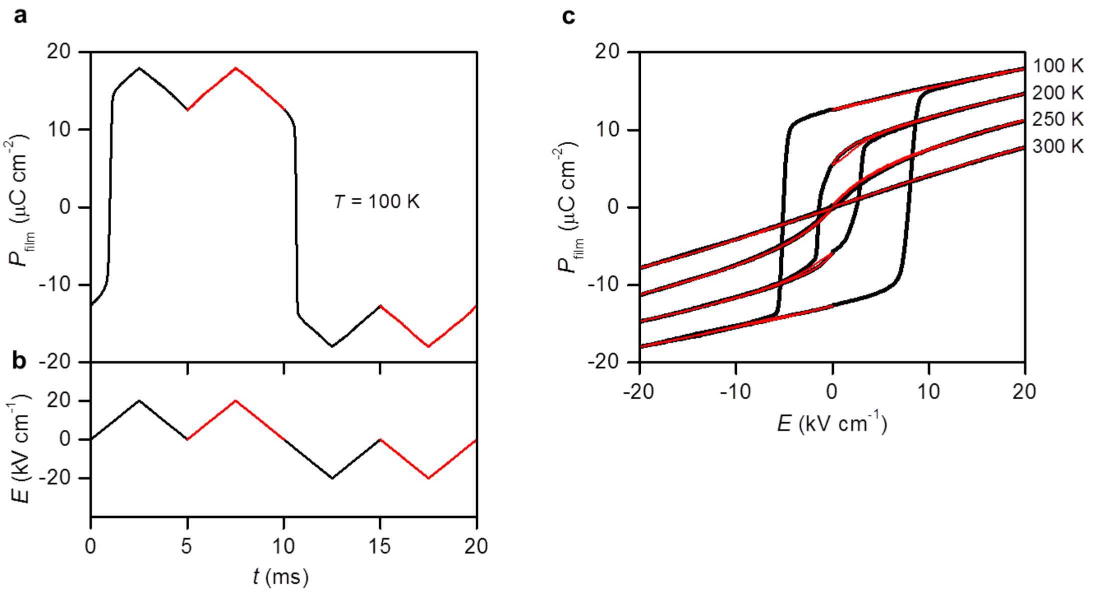

Figure S25. Bipolar and unipolar polarization for STO//DSO. (a-c) Bipolar (unipolar) measurements appear black (red). (a) P_{film} at 100 K, and (b) E versus time t, with 2001 data points. Hence (c) bipolar and unipolar $P_{film}(E)$ at 100 K, along with the corresponding data for three other temperatures. The sample was initialized at each temperature by performing a bipolar cycle prior to t = 0. Data based on measurements of Sample 1 and DSO substrate 1.

Note 13. EC effects in the DSO substrate

Electrical measurements of a DSO substrate were made by addressing it with IDEs that were equivalent to the IDEs used for STO//DSO. Bipolar $P_{sub}(E)$ plots at nearby temperatures (examples in Fig. S11b) were used to map $|P_{sub}(T,E)|$ (not shown), whose cross-sections at selected positive fields yield isofield plots of $P_{sub}(T)$ (Fig. S26a). The entire $|P_{sub}(T,E)|$ map was used to evaluate the isothermal entropy change $|\Delta S_{sub}(T,E)| = |\int_0^E (\partial P_{sub}/\partial T)_{E'} dE'|$ (Fig. S26b) due to an applied electric field E. At our maximum field of 20 kV cm^{-1}, there is no peak in $|\Delta S_{sub}(T,E)|$, and the largest value of $|\Delta S_{sub}| = 0.15$ J K^{-1} m^{-3} is much smaller than the maximum value of $|\Delta S_{film}| \sim 1.8$ kJ K^{-1} m^{-3} for the STO film (Fig. 6a in the main paper).

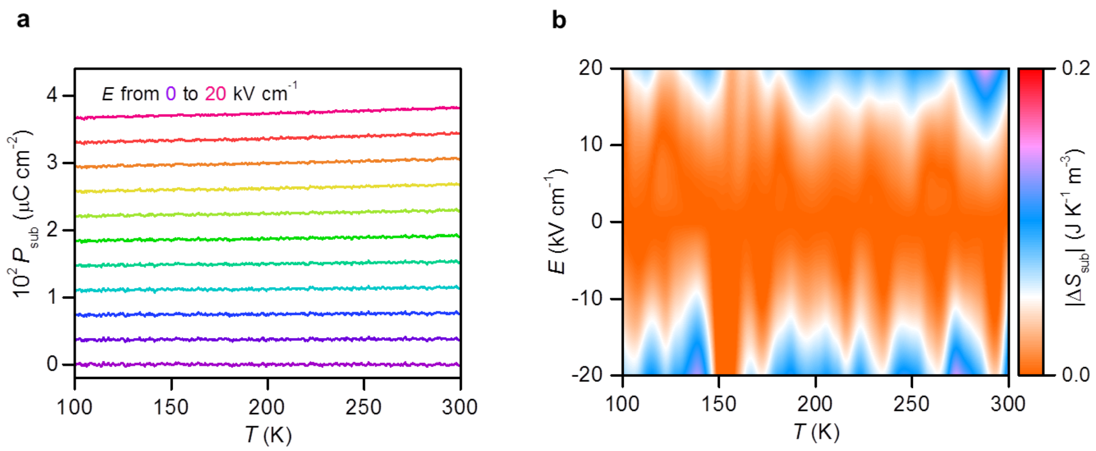

Figure S26. EC effects in the DSO substrate. (a) $P_{sub}(T)$ at constant values of E every 2 kV cm^{-1}. These plots represent transects through a $|P_{sub}(T,E)|$ map (not shown) that was constructed from $P_{sub}(E)$ plots at 636 measurement temperatures (five such plots appear in Fig. S11b). (b) Hence $|\Delta S_{sub}(T,E)|$. The maximum value is $|\Delta S_{sub}| = 0.15$ J K^{-1} m^{-3}. Data based on measurements of DSO substrate 1.

Note 14. Isothermal transects through $|\Delta S_{film}(T,E)|$ for STO//DSO

Fig. 6c in the main paper shows three isothermal $|\Delta S_{film}(E)|$ transects through $|\Delta S_{film}(T,E)|$ (Fig. 6a in the main paper). Fig. S27 shows many such transects, none of which saturate at our maximum field.

Below T_C (Fig. S27a), $|\Delta S_{film}(E)|$ adopts a pinched V-shape because the magnitude of the integrand $(\partial P_{film}/\partial T)_E$ (inferred from Fig. 5f in the main paper) is larger at lower fields and smaller at higher fields.

Above T_C (Fig. S27b), $|\Delta S_{film}(E)|$ adopts a U-shape because the magnitude of $(\partial P_{film}/\partial T)_E$ is smaller at lower fields and larger at higher fields. Just above T_C, the low-field U is relatively narrow as the field-induced enhancement of $(\partial P_{film}/\partial T)_E$ is relatively large.

Each type of transect (pinched V-shape, U-shape, narrow U) is represented in Fig. 6c of the main paper.

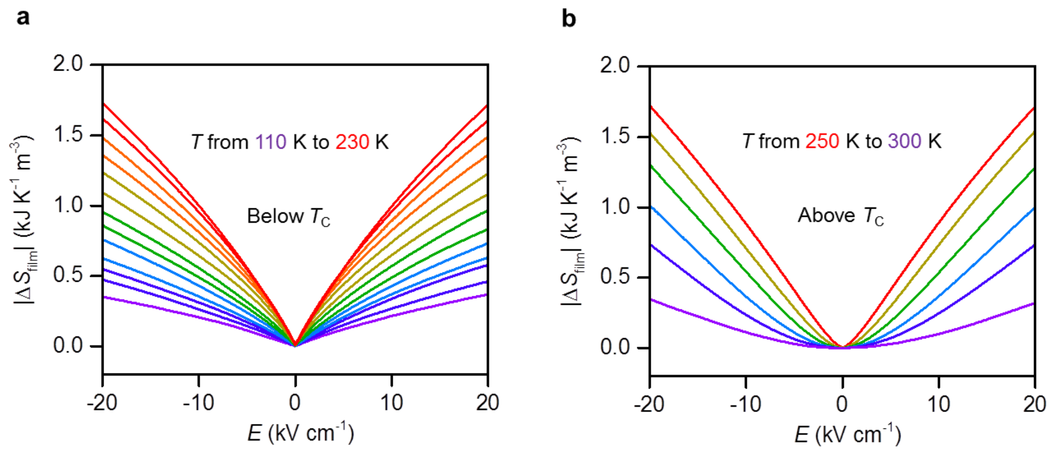

Figure S27. Isothermal transects through $|\Delta S_{film}(T,E)|$ for STO//DSO. Isothermal transects through $|\Delta S_{film}(T,E)|$ (Fig. 6a of the main paper) both (a) below and (b) above T_C. (a) Pinched V-shape plots every 10 K, omitting 240 K data due to great overlap with 230 K data. (b) U-shape plots every 10 K; the U is narrow just above T_C. Data based on measurements of Sample 1 and DSO substrate 1.

Note 15. Isothermal and isofield transects through $|\Delta S_{film}/E(T,E)|$ for STO//DSO

Fig. S28 shows isothermal and isofield transects through our plot of EC strength $|\Delta S_{film}(T,E)/E|$ (Fig. 6d in the main paper). The isothermal transects at 250 K and 280 K (Fig. S28a) show relatively low strengths at low field, consistent with the U-shape $|\Delta S_{film}(E)|$ transects at these temperatures (Fig. S27b). The isothermal transects at 200 K (Fig. S28a) show relatively high strengths at low field, consistent with the pinched V-shape $|\Delta S_{film}(E)|$ transects at this temperature (Fig. S27a).

Our low-field EC strengths exceed 2.0 mJ K^{-1} V^{-1} m^{-2} at 200 K (Fig. S28a), and exceed 1.5 mJ K^{-1} V^{-1} m^{-2} over a wide temperature span of 50 K (Fig. S28b). These strengths are larger than 1.3 mJ K^{-1} V^{-1} m^{-2} for polycrystalline thin-film $PbZr_{0.95}Ti_{0.05}O_3$ (ref. [S14]), and represent some of the largest EC strengths for a film (Table S5 in ref. [S15]).

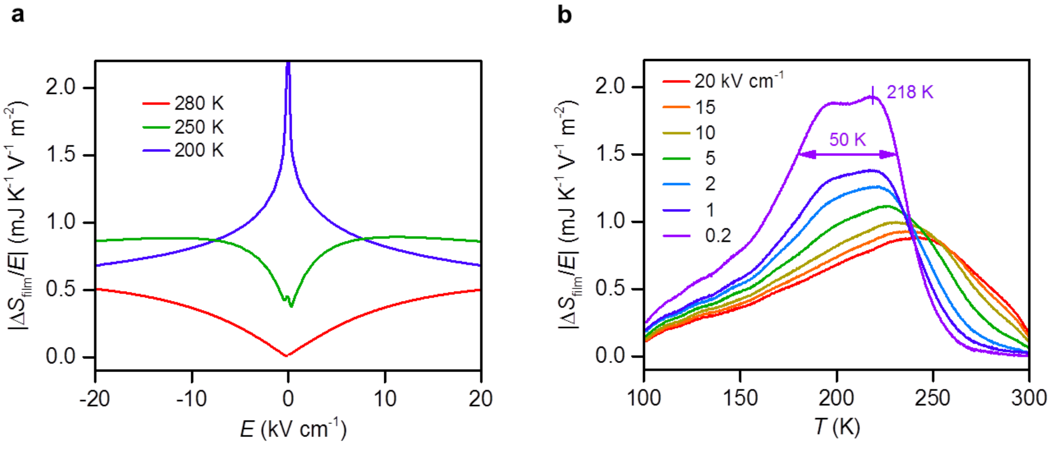

Figure S28. Transects through $|\Delta S_{film}/E(T,E)|$ for STO//DSO. (a) Isothermal and (b) isofield transects through $|\Delta S_{film}/E(T,E)|$ (Fig. 6d of the main paper). In (b), $|\Delta S_{film}/E(T,E)| \geq 1.5$ mJ K^{-1} V^{-1} m^{-2} in a temperature range that spans 50 K (180 K $\leq T \leq$ 230 K, purple arrow). Data based on measurements of Sample 1 and DSO substrate 1.

Note 16. Adiabatic temperature change $|\Delta T_{film}(T_s,E)|$ for the strained STO film

Here we convert $|\Delta S_{film}(T,E)|$ (Fig. 6a in the main paper) to the corresponding adiabatic temperature change $|\Delta T_{film}(T_s,E)| \sim T_s|\Delta S_{film}(T_s,E)|/c(T_s)$ at starting temperature T_s (Fig. S29a), using the zero-field volume-normalized heat capacity $c(T_s)$ (Fig. S30). Both $|\Delta S_{film}(T,E)|$ and $|\Delta T_{film}(T_s,E)|$ take a similar form because $0.79 \leq c(T_s) \leq 1.09$ is reasonably constant in the 100-300 K temperature range of interest (Fig. S30). Adiabatic changes of temperature could in practice be achieved by removing substrate material from a small region of the fully strained film.

Fig. S29b shows isofield transects through $|\Delta T_{film}(T_s,E)|$ (Fig. S29a). The maximum value of $|\Delta T_{film}| \sim 0.17$ K with $|E| = 20$ kV cm^{-1} occurs at $T_s \sim 245$ K, which is similar to $T \sim 242$ K for corresponding peak in $|\Delta S_{film}|$ (Fig. 6a in the main paper).

We compare $|\Delta T_{film}(T_s,E)|$ for our strained film (Fig. S29a) with the corresponding data for bulk STO (Supplementary Note 10) by plotting $|\Delta T_{film}(T_s,E)/\Delta T_{bulk}(T_s,E)|$ in Fig. S29c. Values may be read from selected isofield transects (Fig. S29d), which correspond to the ratio of isofields such as those in Fig. S29b. For our largest field of 20 kV cm^{-1}, there is an order-of-magnitude strain-induced enhancement of EC effects ($|\Delta T_{film}/\Delta T_{bulk}| = 54$ at 257 K), cf. $|\Delta S_{film}/\Delta S_{bulk}| = 53$ at 257 K from Fig. 6f in the main text [the small difference between $|\Delta T_{film}/\Delta T_{bulk}|$ and $|\Delta S_{film}/\Delta S_{bulk}|$ arises because $c_{bulk}(T_s) = 1.014c(T_s)$]. At the low field of 0.2 kV cm^{-1}, the enhancement reaches two orders of magnitude ($|\Delta T_{film}/\Delta T_{bulk}| = 344$ at 226 K), cf. $|\Delta S_{film}/\Delta S_{bulk}| = 339$ at 226 K from Fig. 6f in the main text.

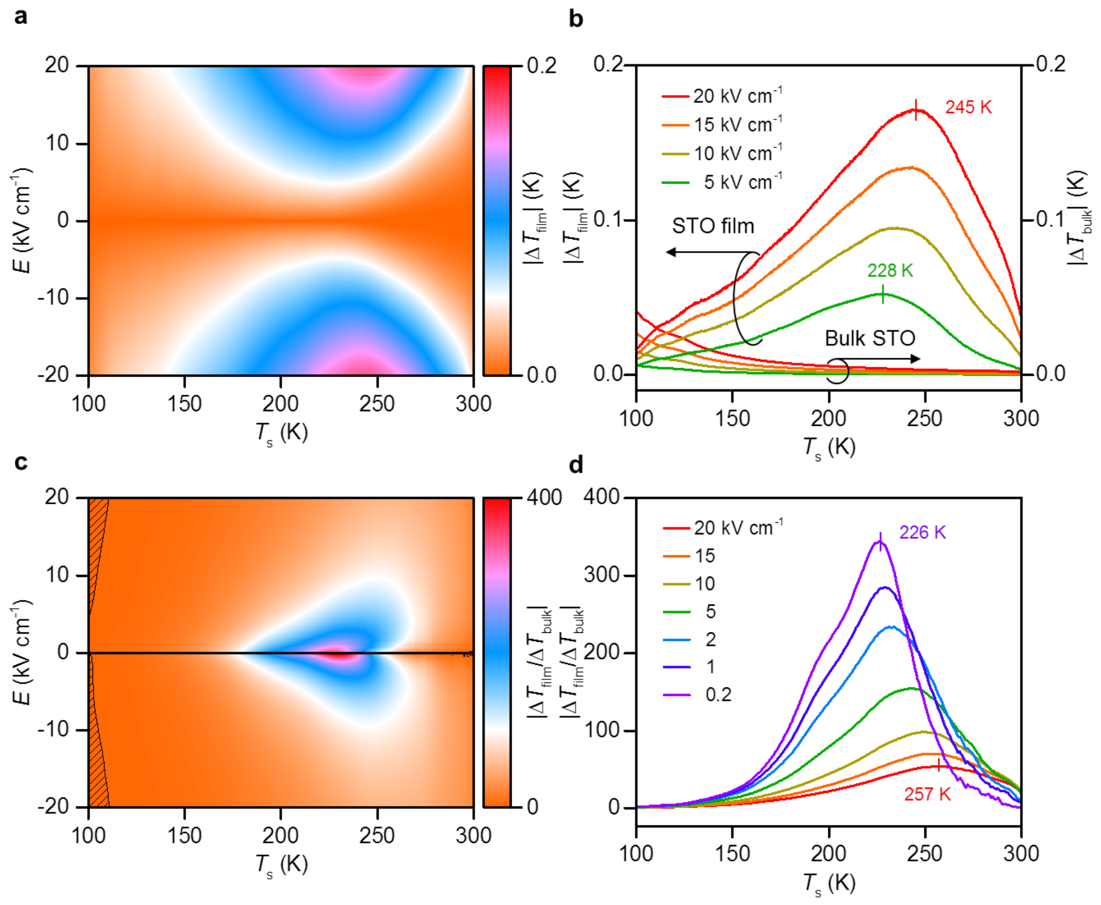

Figure S29. Adiabatic temperature change in the strained STO film. (a) $|\Delta T_{\mathrm{film}}(T_{\mathrm{s}},E)| \sim T_{\mathrm{s}}|\Delta S_{\mathrm{film}}(T_{\mathrm{s}},E)|/c(T_{\mathrm{s}})$ derived from both $|\Delta S_{\mathrm{film}}(T,E)|$ (Fig. 6a in the main paper) and the volume-normalized heat capacity $c(T_{\mathrm{s}})$ (Fig. S30), where T_{s} denotes starting temperature. (b) Isofield $|\Delta T_{\mathrm{film}}(T_{\mathrm{s}})|$ transects through (a) at selected positive fields, and the corresponding data for bulk STO (Supplementary Note 10). (c) $|\Delta T_{\mathrm{film}}(T_{\mathrm{s}},E)/\Delta T_{\mathrm{bulk}}(T_{\mathrm{s}},E)|$ is larger (smaller) than unity in the unhashed (hashed) regions [$|\Delta T_{\mathrm{film}}(T_{\mathrm{s}},E)|$ from (a), $|\Delta T_{\mathrm{bulk}}(T_{\mathrm{s}},E)|$ from Supplementary Note 10), data presented at intervals of 1 K and 0.02 kV cm^{-1} via interpolation]. (d) Isofield transects through (e) at selected positive fields. Data based on measurements of Sample 1, DSO substrate 1, and a single sample of bulk STO.

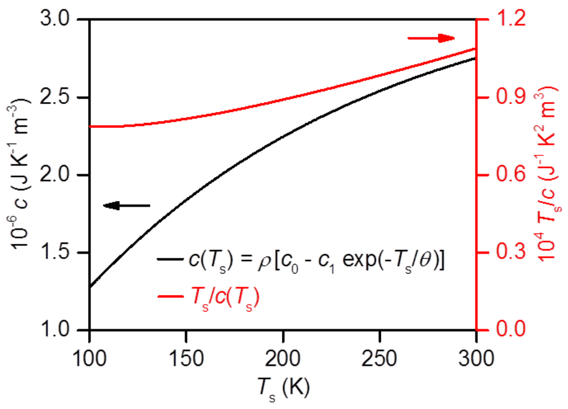

Figure S30. STO film heat capacity and the resulting $\Delta S \rightarrow \Delta T$ conversion factor. Zero-field volume-normalized heat capacity $c(T_s)$ (black) obtained from the bulk specific heat capacity $[c_0 - c_1\exp(-T_s/\theta)$ with $c_0 = 656$ J kg^{-1} K^{-1}, $c_1 = 773$ J kg^{-1} K^{-1}, $\theta = 154$ K][S16] and a film density of $\rho = 5045$ kg m^{-3} (from molar mass 183.5 g mol^{-1} and the room-temperature unit cell volume of 60.4 Å^3 via Fig. 2d in the main paper). The factor of $T_s/c(T_s)$ (red) in $|\Delta T_{film}(T_s,E)| \sim T_s|\Delta S_{film}(T_s,E)|/c(T_s)$ (Fig. S29a) varies between 0.79 and 1.09 in 100-300 K (T_s denotes starting temperature). The film density of $\rho = 5045$ kg m^{-3} differs by just 1.4% from the bulk density of 5118 kg m^{-3}, yielding $c_{bulk}(T_s) = 1.014c(T_s)$ for Fig. S18c and Fig. S29a,b.

Note 17. EC effects in STO//DSO via polarization from integrated dielectric data

Fig. 4 in the main paper shows the dielectric response ε_{film} for the strained STO film at different values of frequency, temperature and bias field, out to a maximum bias field magnitude of 8 kV cm^{-1}. By integrating the dielectric data obtained while reducing bias-field magnitude ($E < 0$ in Fig. 4c of the main paper, $E > 0$ in Fig. 4d of the main paper), we obtain polarization data P_{film} (Fig. S31a-c) that are similar to the measured polarization data (Fig. S31d-f, obtained from Fig. 5d-f in the main paper by plotting data out to 8 kV cm^{-1} rather than 20 kV cm^{-1}). Both polarization datasets therefore yield similar plots of $|\Delta S_{film}|$ (Fig. S32) and $|\Delta T_{film}|$ (Fig. S33).

Small discrepancies in Figs S31-S33 between data derived from ε_{film} and data derived from P_{film} may be due to the failure of the 8 kV cm^{-1} maximum bias field in our dielectric measurements to eliminate any ferroelectric domains below T_C (Fig. 5c in the main paper), the weak relaxor component (Fig. 4a in the main paper), and the small dielectric loss of 2-4% (Supplementary Note 11). By contrast, integration of the measured dielectric data for bulk STO yields polarization data and hence EC data that match very well with the EC data evaluated from the measured polarization data (Supplementary Note 10).

Note that here, the integration of dielectric data does not yield a completely independent method of evaluating EC effects because we obtained remanent polarizations (constants of integration) from polarization data [$P_{film}(T,0)$ in Fig. 5f, $P_{bulk}(T,0)$ in Supplementary Fig. 17c]. Remanent polarizations cannot be correctly obtained by integrating dielectric data, as the a.c. measurement field modifies the irreversible and dynamic ferroelectric domain switching, but remanent polarization values could be independently obtained from zero-field pyroelectric data.

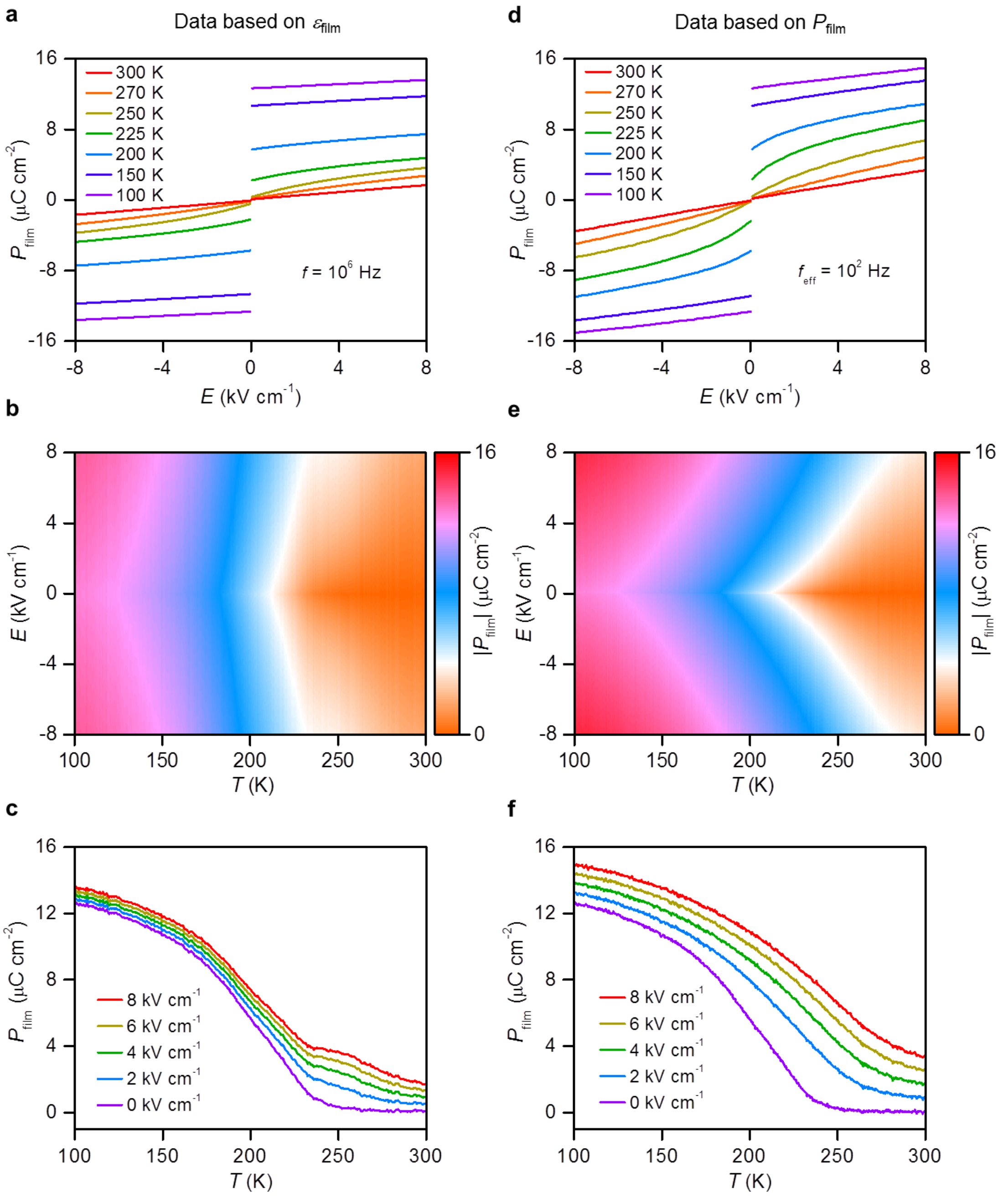

Figure S31. Electrical polarization of the strained STO film via integration of dielectric data and via direct measurements. (a-c) Polarization $P_{film}(T,E) = \int_0^E \varepsilon_0[\varepsilon_{film}(T,E') - 1]dE' + P_{film}(T,0)$ from isothermal measurements of dielectric response $\varepsilon_{film}(T,E)$ while reducing bias-field magnitude ($E < 0$ in Fig. 4c of the main paper, $E > 0$ in Fig. 4d of the main paper) (ε_0 is the permittivity of free space). Integration constant $P_{film}(T,0)$ was identified at all 375 dielectric measurement temperatures by interpolation of remanent unipolar $P_{film}(E)$ values that were evaluated at 668 measurement temperatures (purple plot, Fig. 5f in the main paper). (a) $P_{film}(E)$ at seven measurement temperatures. (b) $|P_{film}(T,E)|$ at all 375 dielectric measurement temperatures. (c) Isofield $P_{film}(T)$ transects through (b) at zero field and selected positive fields. (d-e) Polarization data out to 8 kV cm^{-1} based on field-removal branches of 668 unipolar $P_{film}(E)$ measurements out to 20 kV cm^{-1} (Fig. 5e in the main paper). (d) $P_{film}(E)$ at seven measurement temperatures (subset of data in Fig. 5d in the main paper) (the effective measurement frequency $f_{eff} = 10^2$ Hz for the unipolar $P_{film}(E)$ measurements implies a field-sweep rate whose magnitude would yield bipolar $P_{film}(E)$ plots at frequency 10^2 Hz, Supplementary Note 12). (e) $|P_{film}(T,E)|$ at all 668 measurement temperatures (subset of data in Fig. 5e in the main paper). (f) Isofield $P_{film}(T)$ transects through (e) at zero field and selected positive fields. Data based on measurements of Sample 1 and DSO substrate 1.

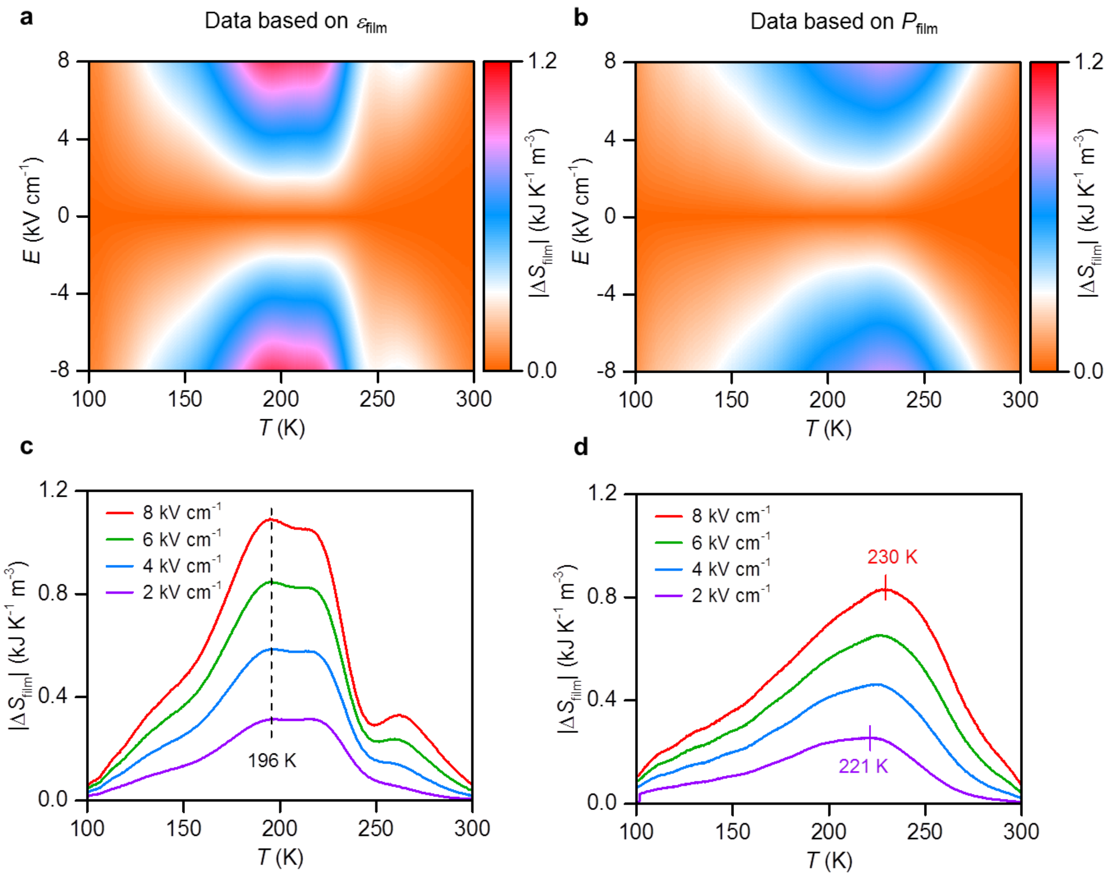

Figure S32. Isothermal EC effects in the strained STO film deduced from integration of dielectric data and measurements of polarization. (a,b) $|\Delta S_{film}(T,E)| = |\int_0^E (\partial P_{film}/\partial T)_{E'} dE'|$ derived from $P_{film}(T,E)$ data obtained by (a) integrating dielectric data (Fig. S31b) and (b) direct measurements (Fig. S31e). (c,d) Isofield $|\Delta S_{film}(T)|$ transects through (a,b), respectively, at selected positive fields. Data based on measurements of Sample 1 and DSO substrate 1.

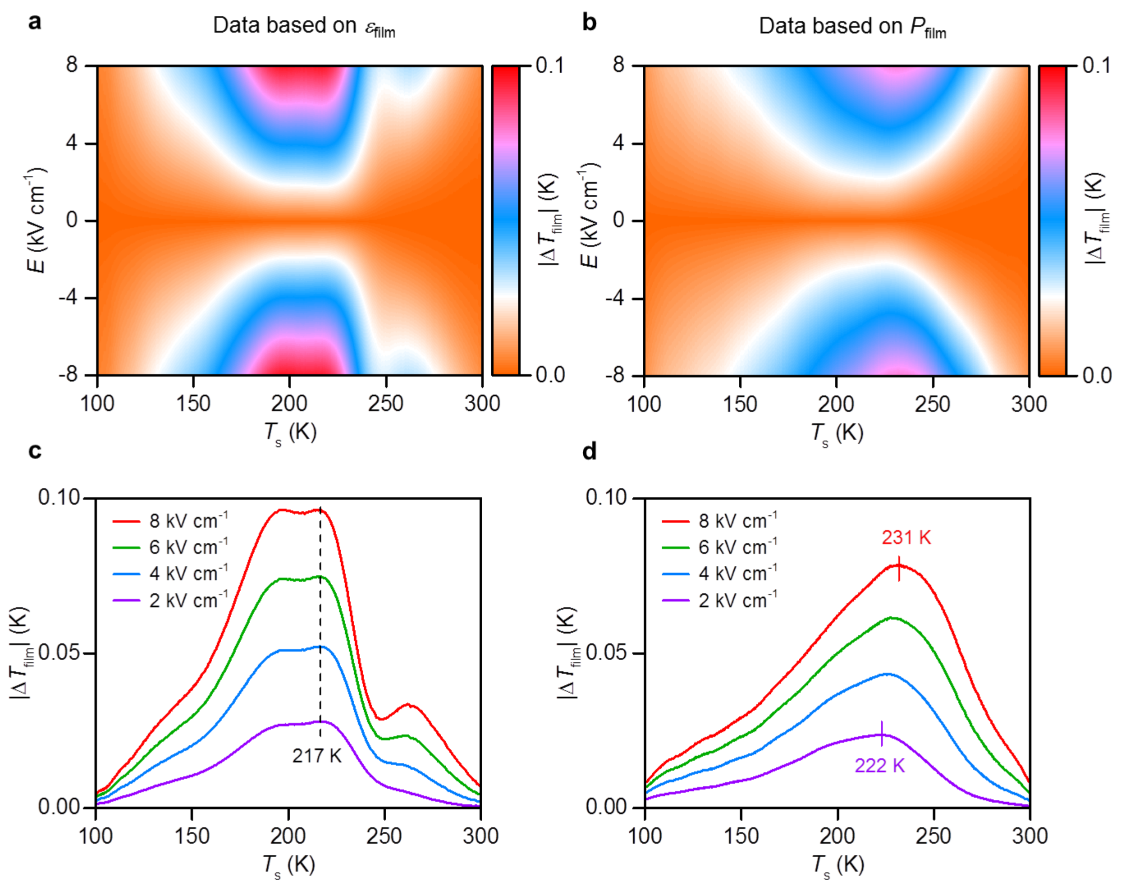

Figure S33. Adiabatic EC effects in the strained STO film deduced from integration of dielectric data and measurements of polarization. (a,b) $|\Delta T_{film}(T_s,E)| \sim T_s|\Delta S_{film}(T_s,E)|/c(T_s)$ derived from $|\Delta S_{film}(T,E)|$ and the volume-normalized heat capacity $c(T_s)$ (Fig. S30), where T_s denotes starting temperature. In (a), we used $|\Delta S_{film}(T,E)|$ (Fig. S32a) based on $|P_{film}(T,E)|$ (Fig. S31b) obtained via integration dielectric data. In (b), we used $|\Delta S_{film}(T,E)|$ (Fig. S32b) based on $|P_{film}(T,E)|$ (Fig. S31e) obtained from direct measurements of polarization. (c,d) Isofield $|\Delta T_{film}(T_s)|$ transects through (a,b), respectively, at selected positive fields. Data based on measurements of Sample 1 and DSO substrate 1.

Note 18. EC effects in different STO//DSO samples

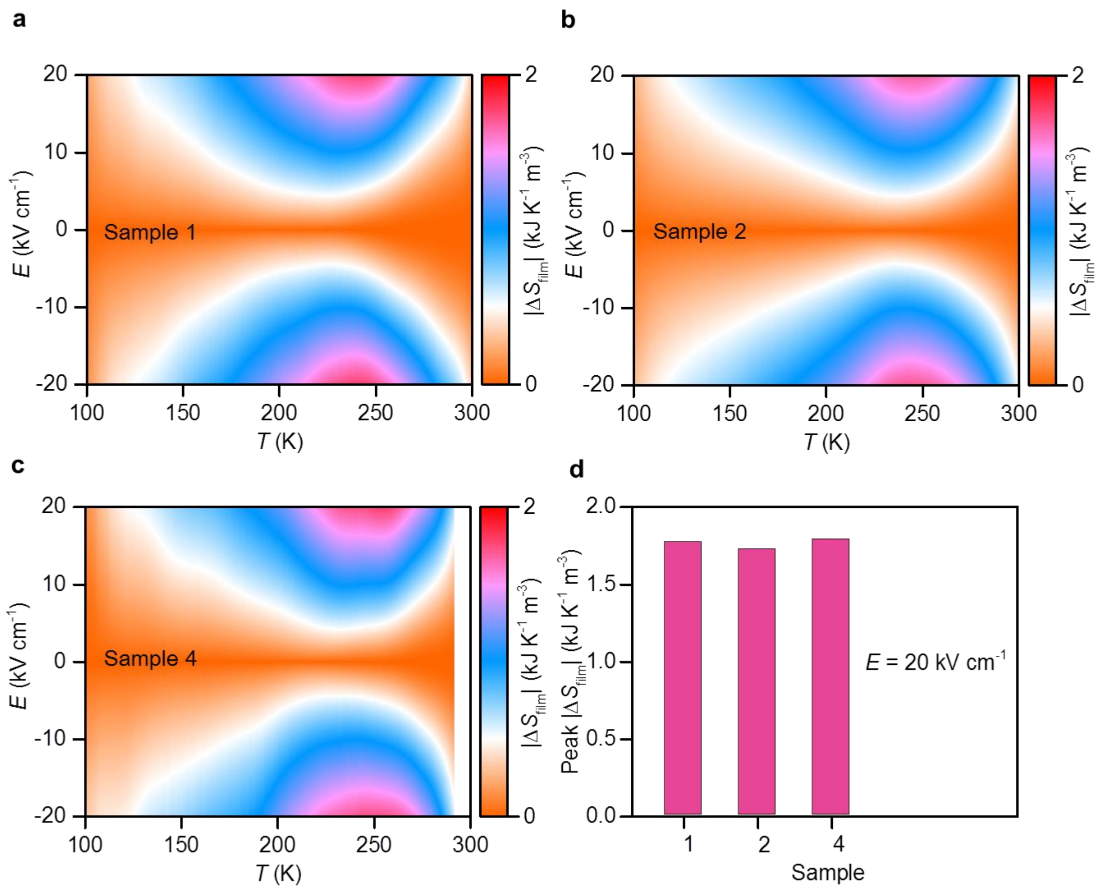

Figure S34. EC effects in different STO//DSO samples. (a-c) $|\Delta S_{\rm film}(T,E)|$ for (a) Sample 1, (b) Sample 2 and (c) Sample 4. (d) Comparison of maximum $|\Delta S_{\rm film}(T,E)|$ values for $|E|$ = 20 kV cm^{-1}. Data for Sample 1 are copied from Fig. 6a.

Note 19. Landau theory of EC effects in STO//DSO

We predict the dielectric response, electrical polarization and EC behaviour of our strained STO film by employing the canonical Landau model[S17,S18] for STO films under equibiaxial in-plane strain S_m, setting $S_m = +1\%$ to match our experimental conditions (Fig. 2d,e in the main paper), and ignoring the 0.02% difference between in-plane principal strains (which is reasonable as the corresponding correction to film energy density is very small). The calculations were performed using Mathematica software.

In (1), we write an expression for the energy density $\widetilde{F}$ of the strained STO film in terms of S_m and 18 other parameters. In (2), we present the parameter constraints that set the observed ferroelectric phase for $S_m = +1\%$, and we investigate phase stability. In (3), we present all 19 model parameters. In (4), we show for properties of interest (dielectric behaviour, polarization and EC effects) that there is good agreement between the predictions of the model and our experimentally derived results, and we discuss the origin of minor discrepancies.

The total polarization comprises the field-dependent symmetry-breaking polarization associated with the ferroelectric transition (P_1 in the Landau free energy, P at equilibrium) and a field-induced polarization that is not captured by Landau theory, as explained in ref. [S19], and as seen later by comparing Fig. S37c and Fig. S37d. We infer that these field-induced contributions show no substantial temperature dependence as the EC predictions of the Landau model reproduce well the EC predictions of the indirect method (Fig. S38-S39).

Notation

- The ‘film’ subscript is omitted in all Landau work in order to facilitate the use of other subscripts, and distinguish Landau parameters from those derived from experiment.
- At zero field, the equilibrium value of P_1 is the spontaneous polarization P_s.
- At finite field, the equilibrium value of P_1 is denoted P, and setting $E_1 = E$ permits us to report $P(T,E)$ with no subscripts.

(1) Energy density

For bulk STO at temperature T, with directions i = 1, 2, 3 parallel to the <001>$_{pc}$ lattice directions, the relevant order parameters are the polarization $\boldsymbol{P} = (P_1, P_2, P_3)$ and an antiferrodistortive distortion $\boldsymbol{q} = (q_1, q_2, q_3)$. In an applied electric field $\boldsymbol{E} = (E_1, E_2, E_3)$ and under strain S_i (i = 1, 2, ..., 6), the Helmoltz free energy density F of STO is given by the following expansion:

$$
\begin{aligned}
F = {} & F_0 + \alpha_1\left(P_1^2 + P_2^2 + P_3^2\right) + \alpha_{11}\left(P_1^4 + P_2^4 + P_3^4\right) + \alpha_{12}\left(P_1^2P_2^2 + P_1^2P_3^2 + P_2^2P_3^2\right) \\
& + \beta_1\left(q_1^2 + q_2^2 + q_3^2\right) + \beta_{11}\left(q_1^4 + q_2^4 + q_3^4\right) + \beta_{12}\left(q_1^2q_2^2 + q_1^2q_3^2 + q_2^2q_3^2\right) \\
& + \tfrac{1}{2} C_{11}\left(S_1^2 + S_2^2 + S_3^2\right) + C_{12}(S_1S_2 + S_1S_3 + S_2S_3) + \tfrac{1}{2} C_{44}\left(S_4^2 + S_5^2 + S_6^2\right) \\
& - g_{11}\left(S_1P_1^2 + S_2P_2^2 + S_3P_3^2\right) - g_{12}\left[S_1\left(P_2^2 + P_3^2\right) + S_2\left(P_1^2 + P_3^2\right) + S_3\left(P_1^2 + P_2^2\right)\right] \\
& - g_{44}(S_4P_2P_3 + S_5P_1P_3 + S_6P_1P_2) - \lambda_{11}\left(S_1q_1^2 + S_2q_2^2 + S_3q_3^2\right) \\
& - \lambda_{12}\left[S_1\left(q_2^2 + q_3^2\right) + S_2\left(q_1^2 + q_3^2\right) + S_3\left(q_1^2 + q_2^2\right)\right] - \lambda_{44}\left(S_4q_2q_3 + S_5q_1q_3 + S_6q_1q_2\right) \\
& - t_{11}\left(P_1^2q_1^2 + P_2^2q_2^2 + P_3^2q_3^2\right) - t_{12}\left[P_1^2\left(q_2^2 + q_3^2\right) + P_2^2\left(q_1^2 + q_3^2\right) + P_3^2\left(q_1^2 + q_2^2\right)\right] \\
& - t_{44}\left(P_1P_2q_1q_2 + P_1P_3q_1q_3 + P_2P_3q_2q_3\right) - (E_1P_1 + E_2P_2 + E_3P_3)\ ,
\end{aligned}
$$

where F_0 is the free energy when $P_i = q_i = E_i = S_i = 0$; α_i, β_i are temperature-dependent functions (see Table 1 in Section (3) of this Supplementary Note); α_{ij}, β_{ij}, g_{ij}, λ_{ij}, t_{ij} are assumed to be temperature independent; and C_{ij} are the elastic constants of the high-temperature cubic phase that we assume to be temperature independent and valid below the Curie temperature.

Our uniformly strained STO film has a mechanically free upper surface, and we will set the x_3 axis orthogonal to the film-substrate interface. The energy density $\widetilde{F}$ of our film is described by the Legendre transformation of F:

$$\widetilde{F} = F - \sigma_3S_3 - \sigma_4S_4 - \sigma_5S_5\ ,$$

where the mechanical stresses $\sigma_{3,4,5}$ are defined under the assumption of an undistorted (rectangular) reference frame. These stresses are all zero ($\sigma_{3,4,5} = 0$), such that $\widetilde{F} = F$.

Our film has fixed values of S_1, S_2, and $S_6 = 0$, where $S_1 = S_2 = S_m$ for misfit strain $S_m = \frac{a_{film} - a_{bulk}}{a_{bulk}}$. Strains S_3, S_4, and S_5 are determined by minimizing the energy density $\frac{\partial \widetilde{F}}{\partial S_{3,4,5}} = 0$, such that:

$$S_3 = \frac{1}{C_{11}}\left[g_{12}\left(P_1^2 + P_2^2\right) + g_{11}P_3^2 + \lambda_{12}\left(q_1^2 + q_2^2\right) + \lambda_{11}q_3^2 - 2C_{12}S_m\right]\ ,$$

$$S_4 = \frac{g_{44}}{C_{44}}P_2P_3 + \frac{\lambda_{44}}{C_{44}}q_2q_3\ ,$$

$$S_5 = \frac{g_{44}}{C_{44}}P_1P_3 + \frac{\lambda_{44}}{C_{44}}q_1q_3\ .$$

By substituting for $S_{1\text{–}6}$ into $\widetilde{F}$, we find:

$$\widetilde{F} = F_0 + \frac{1}{C_{11}}\left(C_{11}^2 + C_{11}C_{12} - 2C_{12}^2\right)S_m^2 + \tilde{\alpha}_1\left(P_1^2 + P_2^2\right) + \tilde{\alpha}_3 P_3^2 + \tilde{\alpha}_{11}\left(P_1^4 + P_2^4\right) + \tilde{\alpha}_{33}P_3^4$$
$$+ \tilde{\alpha}_{12}(P_1P_2)^2 + \tilde{\alpha}_{13}\left(P_1^2 + P_2^2\right)P_3^2 + \tilde{\beta}_1\left(q_1^2 + q_2^2\right) + \tilde{\beta}_3 q_3^2 + \tilde{\beta}_{11}\left(q_1^4 + q_2^4\right) + \tilde{\beta}_{33}q_3^4 + \tilde{\beta}_{12}\left(q_1q_2\right)^2$$
$$+ \tilde{\beta}_{13}\left(q_1^2 + q_2^2\right)q_3^2 - \tilde{t}_{11}\left(P_1^2q_1^2 + P_2^2q_2^2\right) - \tilde{t}_{33}P_3^2q_3^2 - \tilde{t}_{12}\left(P_1^2q_2^2 + P_2^2q_1^2\right) - \tilde{t}_{13}\left(P_1^2 + P_2^2\right)q_3^2$$
$$- \tilde{t}_{31}P_3^2\left(q_1^2 + q_2^2\right) - \tilde{t}_{44}\left(P_1P_2q_1q_2 + P_1P_3q_1q_3 + P_2P_3q_2q_3\right) - \left(E_1P_1 + E_2P_2 + E_3P_3\right),$$

where the renormalized parameters $\tilde{\alpha}_i$, $\tilde{\alpha}_{ij}$, $\tilde{\beta}_i$, $\tilde{\beta}_{ij}$, $\tilde{t}_{ij}$ are given as follows:

$$\tilde{\alpha}_1 = \alpha_1 - \left(g_{11} + g_{12} - \frac{2C_{12}}{C_{11}}g_{12}\right)S_m\,,\ \tilde{\alpha}_3 = \alpha_1 + 2\left(\frac{C_{12}}{C_{11}}g_{11} - g_{12}\right)S_m\,,$$
$$\tilde{\alpha}_{11} = \alpha_{11} - \frac{g_{12}^2}{2C_{11}}\,,\ \tilde{\alpha}_{33} = \alpha_{11} - \frac{g_{11}^2}{2C_{11}}\,,$$
$$\tilde{\alpha}_{12} = \alpha_{12} - \frac{g_{12}^2}{C_{11}}\,,\ \tilde{\alpha}_{13} = \alpha_{12} - \frac{g_{11}g_{12}}{C_{11}} - \frac{g_{44}^2}{2C_{44}}\,,$$
$$\tilde{\beta}_1 = \beta_1 - \left(\lambda_{11} + \lambda_{12} - \frac{2C_{12}}{C_{11}}\lambda_{12}\right)S_m\,,\ \tilde{\beta}_3 = \beta_1 + 2\left(\frac{C_{12}}{C_{11}}\lambda_{11} - \lambda_{12}\right)S_m\,,$$
$$\tilde{\beta}_{11} = \beta_{11} - \frac{\lambda_{12}^2}{2C_{11}}\,,\ \tilde{\beta}_{33} = \beta_{11} - \frac{\lambda_{11}^2}{2C_{11}}\,,$$
$$\tilde{\beta}_{12} = \beta_{12} - \frac{\lambda_{12}^2}{C_{11}}\,,\ \tilde{\beta}_{13} = \beta_{12} - \frac{\lambda_{11}\lambda_{12}}{C_{11}} - \frac{\lambda_{44}^2}{2C_{44}}\,,$$
$$\tilde{t}_{11} = t_{11} + \frac{g_{12}\lambda_{12}}{C_{11}}\,,\ \tilde{t}_{33} = t_{11} + \frac{g_{11}\lambda_{11}}{C_{11}}\,,\ \tilde{t}_{12} = t_{12} + \frac{g_{12}\lambda_{12}}{C_{11}}\,,$$
$$\tilde{t}_{13} = t_{12} + \frac{g_{12}\lambda_{11}}{C_{11}}\,,\ \tilde{t}_{31} = t_{12} + \frac{g_{11}\lambda_{12}}{C_{11}}\,,\ \tilde{t}_{44} = t_{44} + \frac{g_{44}\lambda_{44}}{C_{44}}\,.$$

We will use our experimentally determined value of S_m, and take all other parameters from ref. [S17] except for α_{12} (ref. [S20]) and α_{11} and g_{11} (fitted to data).

(2) The stable ferroelectric phase with S_m = +1%

Three possible ferroelectric phases

An equibiaxial in-plane strain of S_m = +1% implies $\boldsymbol{q}$ = 0 for all temperatures above ~115 K (Fig. 4 in ref. [S20]). For simplicity, we will also set $\boldsymbol{q}$ = 0 below ~115 K, which is reasonable as the data we show below ~115 K falls in a small range of temperatures that lies far from temperatures of interest near T_C = 243 K. For a ferroelectric phase which thus has $\boldsymbol{q}$ = 0, it is shown in ref. [S20] that our zero-field energy density $\tilde{F}$ permits the following spontaneous polarizations below the Curie temperature:

$$\boldsymbol{O}_1^\mathrm{F}: (P_1, P_2, P_3) = P_\mathrm{s}(1, 0, 0) \; ,$$

$$\boldsymbol{O}_2^\mathrm{F}: (P_1, P_2, P_3) = \frac{P_\mathrm{s}}{\sqrt{2}}(1, 1, 0) \; ,$$

$$\boldsymbol{T}_1^\mathrm{F}: (P_1, P_2, P_3) = P_\mathrm{s}(0, 0, 1) \; ,$$

where P_s is the magnitude of the spontaneous polarization, and $\boldsymbol{O}$ and $\boldsymbol{T}$ denote orthorhombic and tetragonal symmetries, respectively. The corresponding energy densities are:

$$\boldsymbol{O}_1^\mathrm{F}: \tilde{F}_{100} = \tilde{\alpha}_1 P_\mathrm{s}^2 + \tilde{\alpha}_{11} P_\mathrm{s}^4 \; ,$$

$$\boldsymbol{O}_2^\mathrm{F}: \tilde{F}_{110} = \tilde{\alpha}_1 P_\mathrm{s}^2 + \frac{1}{2}\left(\tilde{\alpha}_{11} + \frac{1}{2}\tilde{\alpha}_{12}\right) P_\mathrm{s}^4 \; ,$$

$$\boldsymbol{T}_1^\mathrm{F}: \tilde{F}_{001} = \tilde{\alpha}_3 P_\mathrm{s}^2 + \tilde{\alpha}_{33} P_\mathrm{s}^4 \; ,$$

where we have dropped the first two terms $[F_0 + \frac{1}{C_{11}}\left(C_{11}^2 + C_{11}C_{12} - 2C_{12}^2\right)S_\mathrm{m}^2]$ from our general expression for $\tilde{F}$, as these terms are constant and independent of P_s.

The stable $\boldsymbol{O}_1^\mathrm{F}$ phase

The experimentally observed in-plane polarization lies along $[100]_{pc}$ (Supplementary Note 1), and so $\boldsymbol{O}_1^\mathrm{F}$ is the global minimum. By considering leading terms in the energy expressions above, we have $\tilde{\alpha}_1 - \tilde{\alpha}_3 < 0$ to ensure that the minima representing the $\boldsymbol{O}_{1,2}^\mathrm{F}$ phases lie at lower energies than the minimum representing the $\boldsymbol{T}_1^\mathrm{F}$ phase. Then, by comparing the energies of the $\boldsymbol{O}_{1,2}^\mathrm{F}$ phases, we have $\tilde{\alpha}_{11} - \frac{1}{2}\tilde{\alpha}_{12} < 0$ to ensure that the $\boldsymbol{O}_1^\mathrm{F}$ phase lies at lower energy than the $\boldsymbol{O}_2^\mathrm{F}$ phase. Given that the thermally driven transition is second order (Fig. 4a and Fig. 5f in the main paper), we have $\tilde{\alpha}_{11} > 0$.

Under the assumption of the three inequalities above, we evaluate fit parameters in the next section (Table 1). Here we use these fit parameters to present a stability diagram for the $\boldsymbol{O}_{1,2}^\mathrm{F}$ phases (Fig. S35a), and we see that our film lies near the boundary between these two phases. We also use our fit parameters to plot the energies of the $\boldsymbol{O}_{1,2}^\mathrm{F}$ phases as a function of polarization P_1 at selected temperatures (Fig. S35b). At 100 K, and thus below the Curie temperature (T_C = 243 K, Fig. 4a in the main paper), the global minimum with finite polarization P_s is correctly associated with the $\boldsymbol{O}_1^\mathrm{F}$ phase.

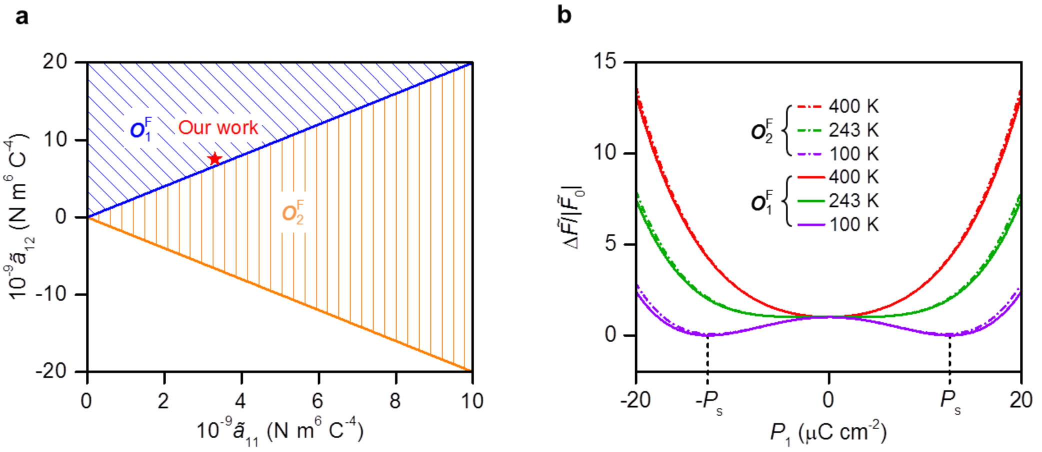

Figure S35. Stability of the zero-field ferroelectric phase in the strained STO film. (a) The $\boldsymbol{O}_1^{\mathrm{F}}$ phase (blue oblique stripes) and $\boldsymbol{O}_2^{\mathrm{F}}$ phase (orange vertical stripes) represent global minima in the energy density $\tilde{F}$. Our STO film adopts the $\boldsymbol{O}_1^{\mathrm{F}}$ phase (red star). The white region represents first-order transitions of no interest here. (b) For the $\boldsymbol{O}_1^{\mathrm{F}}$ phase (solid plots) and the $\boldsymbol{O}_2^{\mathrm{F}}$ phase (dot-dash plots) at selected temperatures, we plot the dependence on the electrical polarization P_1 of the energy density $\Delta\tilde{F}$ with respect to the 100 K global minimum of $\tilde{F}_0 \sim -0.8$ MJ m^{-3} at $P_{\mathrm{s}} = \pm 12.5$ μC cm^{-2}. Note that $\tilde{F}_0$ is calculated here without the first two constant terms of $F_0 + \frac{1}{C_{11}}(C_{11}^2 + C_{11}C_{12} - 2C_{12}^2)S_{\mathrm{m}}^2$. Data from Table 1 in Section (3) of this Supplementary Note.

(3) The 19 model parameters

Table 1 presents all 19 model parameters:

- We set S_m = +1% as explained at the start of this Note.
- We obtained 15 parameters from the canonical Landau parameterization[S17].
- We obtained α_{12} from ref. [S20] rather than ref. [S17] in order to set the $\boldsymbol{O}_1^{\mathrm{F}}$ phase via the aforementioned constraint $\tilde{\alpha}_{11} - \frac{1}{2}\tilde{\alpha}_{12} < 0$. Given that the $\boldsymbol{O}_1^{\mathrm{F}}$ phase has its global free energy minimum along one of the $<100_{>pc}$ directions[S21], it is consistent with our experimental findings (Supplementary Note 1). (The canonical Landau model[S17] also predicted $<100_{>pc}$ originally, but recalculation[S18] with the same input parameters revised this to $<110_{>pc}$.)
- We obtained α_{11} and g_{11} by fitting $\tilde{\alpha}_1(T_C) = 0$ and $2\tilde{\alpha}_1 P + 4\tilde{\alpha}_{11} P^3 = E$ [see part (b) in section (4)] while:
  - setting $\boldsymbol{O}_1^{\mathrm{F}}$ via the aforementioned constraints $\tilde{\alpha}_1 - \tilde{\alpha}_3 < 0$ and $\tilde{\alpha}_{11} - \frac{1}{2}\tilde{\alpha}_{12} < 0$;
  - ensuring that the thermally driven transition is second order via the aforementioned constraint $\tilde{\alpha}_{11} > 0$;
  - setting T_C = 243 K (Fig. 4a in the main paper); and
  - setting a spontaneous low-temperature polarization of P_s = 12.5 μC cm^{-2} (Fig. 5c of the main paper).

Parameter	Units (SI)	Expression or value	Ref.
α_1	N m^2 C^{-2}	4.05×10^7[coth(54/T) – coth(54/30)]	S17
α_{11}	N m^6 C^{-4}	3.31×10^9	Fitted
α_{12}	N m^6 C^{-4}	7.52×10^9	S20
β_1	N m^{-4}	1.32×10^{29}[coth(145/T) – coth(145/105)]	S17
β_{11}	N m^{-6}	1.94×10^{50}	S17
β_{12}	N m^{-6}	3.96×10^{50}	S17
C_{11}	N m^{-2}	3.36×10^{11}	S17
C_{12}	N m^{-2}	1.07×10^{11}	S17
C_{44}	N m^{-2}	1.27×10^{11}	S17
g_{11}	N m^2 C^{-2}	1.47×10^{10}	Fitted
g_{12}	N m^2 C^{-2}	-0.11×10^{10}	S17
g_{44}	N m^2 C^{-2}	0.24×10^{10}	S17
λ_{11}	N m^{-4}	1.3×10^{30}	S17
λ_{12}	N m^{-4}	-2.5×10^{30}	S17
λ_{44}	N m^{-4}	-2.3×10^{30}	S17
t_{11}	N C^{-2}	-3.37×10^{29}	S17
t_{12}	N C^{-2}	0.14×10^{29}	S17
t_{44}	N C^{-2}	6.3×10^{29}	S17
S_m	-	+1%	*

Table S1. Model parameters for the strained STO film. Both α_1 and β_1 vary with T; α_{12} appears as 9.28×10^{-12} cm^6 dyn esu^{-4} in ref. [S20]; and * = Fig. 2d,e in the main paper.

(4) STO//DSO: predictions of the model versus results derived from experiment

Here we show for properties of interest (dielectric behaviour, polarization and EC effects) that there is good agreement between the predictions of the model and our experimentally derived results. Small discrepancies may be attributed to the truncation of the Landau free energy expansion and four material-dependent factors, namely the weak relaxor component (Fig. 4a in the main paper), the small dielectric loss of 2-4% (Supplementary Note 11), small departures from our conformal-mapping assumption of isotropic and homogeneous media (see main paper introduction), and the very small strain relaxation at the upper surface of our otherwise coherently strained film (Fig. 2d,e in the main paper). Two material-independent factors (finite sample size, experimental detection) explain why the dielectric peak measured at zero bias field is not infinite as predicted (Fig. S36).

(a) Relative dielectric permittivity $\varepsilon_r(T,E)$

Landau prediction. Fig. S36a shows $\varepsilon_r(T)$ at selected values of E, evaluated from $\varepsilon_r(T,E) = \chi(T,E) + 1$, where the in-plane susceptibility χ is given via the eigenvalues of the Hessian matrix:

$$\chi_i^{-1} = \varepsilon_0 \left| \frac{\partial^2 \widetilde{F}}{\partial \widehat{P}_i^2} \right|_{\substack{\boldsymbol{q} = (0,\,0,\,0) \\ \boldsymbol{P} = (P,\,0,\,0)}} = \varepsilon_0(2\tilde{\alpha}_1 + 12\tilde{\alpha}_{11}P^2)\,.$$

Here, $\widehat{P}_i$ is the component of polarization along the ith principal axis, and ε_0 is the permittivity of free space. Fig. S36b shows the corresponding information derived from experiment.

Comparison. The Landau prediction of $\varepsilon_r(T)$ matches quite well the corresponding dielectric response $\varepsilon_{\text{film}}(T)$, as shown for selected values of E in Fig. S36. However, the measured peak is finite at zero field because of finite sample size and the finite magnitude of the a.c. measurement field; and the field-driven shift of peak temperature $\Delta T_0 \propto E^{0.66}$, which is necessarily consistent with $\Delta T_0 \propto E^{2/3}$ for second-order phase transitions[S22], differs slightly from the observed shift of $\Delta T_0 \propto E^{0.81}$.

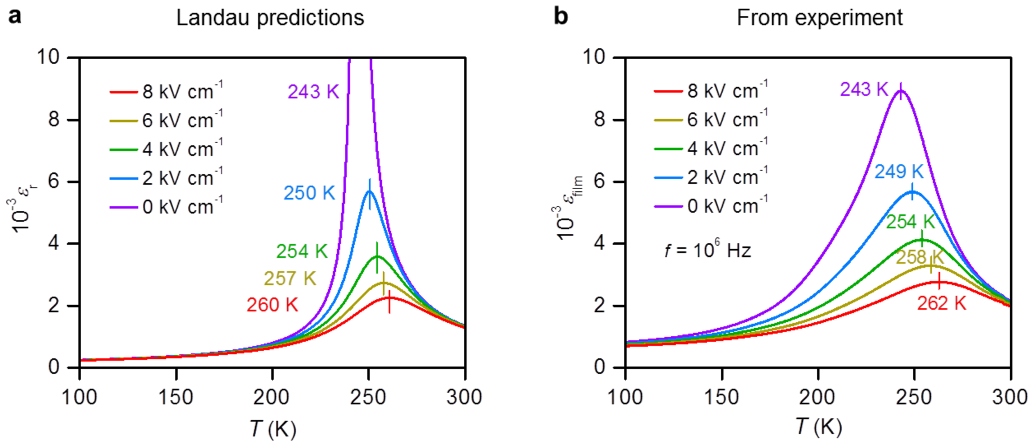

Figure S36. Dielectric response of the strained STO film. (a) Landau prediction of $\varepsilon_r(T)$ at selected values of bias field E, $T_C = 243$ K is an input parameter. (b) The corresponding information derived from experiment (Fig. 4e in the main paper).

(b) $|P(T,E)|$

Landau prediction. Fig. S37a shows $|P(T,E)|$ (selected isofield transects in Fig. S37b), evaluated from the equation of state $2\tilde{\alpha}_1 P + 4\tilde{\alpha}_{11} P^3 = E$, which is obtained from:

$$\left.\frac{\partial \tilde{F}}{\partial P_i}\right|_{\substack{\boldsymbol{q}=(0,0,0)\\ \boldsymbol{P}=(P,0,0)}} = 0,$$

after setting $E_1 = E$.

Comparison. The Landau model reproduces quite well the temperature and field dependences of the polarization (Fig. S37). However, the gradient of the zero-field polarization does not display the predicted discontinuity at T_C, and at all measurement temperatures the Landau polarization necessarily shows a smaller field-enhancement than the measured total polarization (total polarization equals field-dependent Landau polarization plus additional field-induced polarization[S19]).

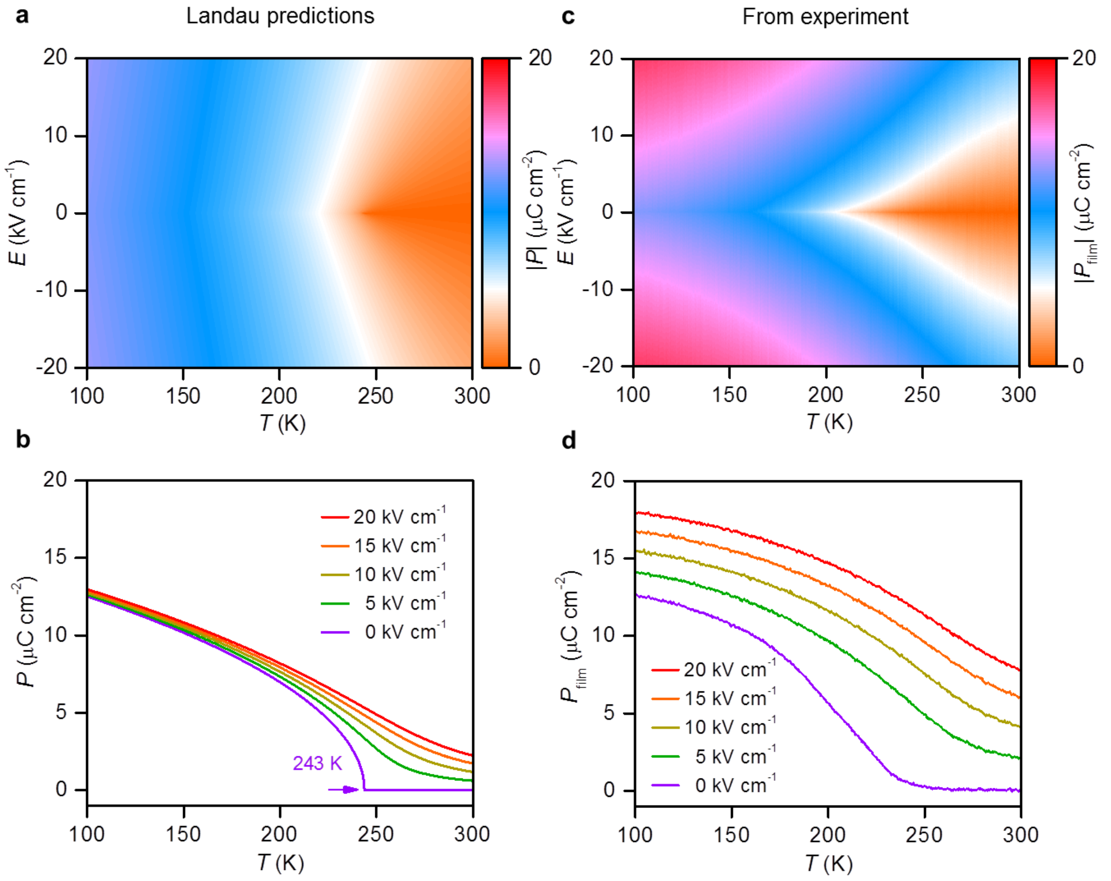

Figure S37. Ferroelectric properties of the strained STO film. (a,b) Landau prediction of (a) $|P(T,E)|$ and (b) $P(T)$ transects at $E = 0$ and selected positive fields. (c,d) The corresponding information derived from experiment (Fig. 5e,f in the main paper).

(c) EC effects

Landau prediction. The entropy of our film is given by $S = S_0 + S_\mathrm{L}$, where S_0 is the entropy when $F = F_0$ (i.e. $P_i = q_i = E_i = S_i = 0$), and where:

$$S_\mathrm{L}(T,E) = -\left.\frac{\partial \widetilde{F}}{\partial T}\right|_{\substack{\boldsymbol{q} = (0,\,0,\,0) \\ \boldsymbol{P} = (P,\,0,\,0)}} = -\left(\frac{\partial \tilde{\alpha}_1}{\partial T}\right) P^2 \, .$$

Note that S_0 and S_L are not to be confused with strain $S_i = S_1, S_2, S_3$.

Using the above expression for S_L, we generate a map of isothermal entropy change $|\Delta S(T,E)| = |S_\mathrm{L}(T,0) - S_\mathrm{L}(T,E)|$ (Fig. S38a), which could equally be generated from the Landau map of $|P(T,E)|$ (Fig. S37a) by using the well known indirect method that we employ in the main paper. Selected isofield transects $|\Delta S(T)|$ through the $|\Delta S(T,E)|$ map are shown in Fig. S38b. For completeness, we show in Fig. S39a the corresponding adiabatic temperature change $|\Delta T(T_\mathrm{s},E)| \sim T_\mathrm{s}|\Delta S(T_\mathrm{s},E)|/c(T_\mathrm{s})$, where T_s denotes starting temperature. Selected isofield transects $|\Delta T(T_\mathrm{s})|$ through the $|\Delta T(T_\mathrm{s},E)|$ map are shown in Fig. S37b.

Comparison. The Landau model captures well the EC behaviour of the film (Fig. S38), first by predicting a peak entropy change (2.1 kJ K^{-1} m^{-3}) that is similar to the corresponding value obtained via experiment (1.8 kJ K^{-1} m^{-3}), and second by predicting an $|E|^{2/3}$ variation in the magnitude of this peak that matches well the $|E|^{0.72}$ variation obtained via experiment ($|E|^{2/3}$ is also consistent with a microscopic model[S23]). However, the Landau model predicts a peak entropy change that is too sharp in temperature and does not shift with field.

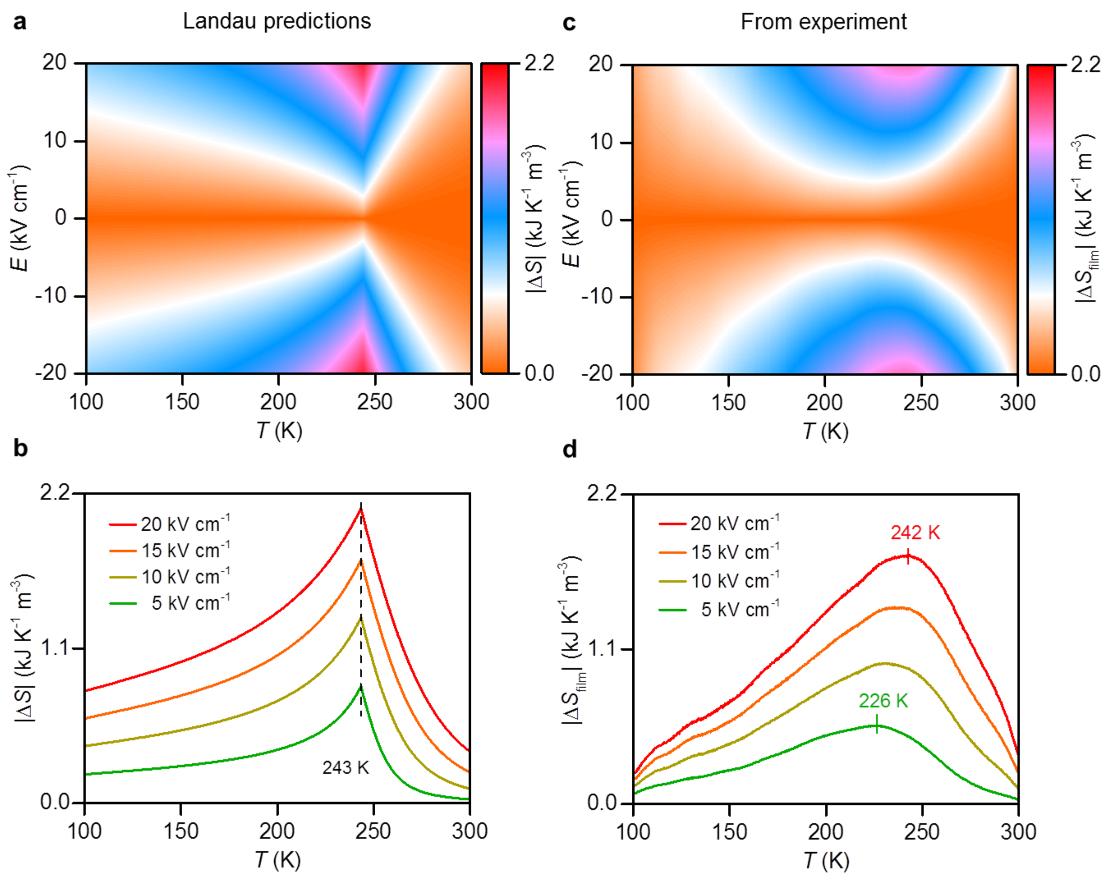

Figure S38. EC entropy change in the strained STO film. (a,b) Landau prediction of (a) $|\Delta S(T,E)| = |S_L(T,0) - S_L(T,E)|$ and (b) $|\Delta S(T)|$ transects at selected positive fields. (c,d) The corresponding information derived from experiment (after Fig. 6a,b in the main paper).

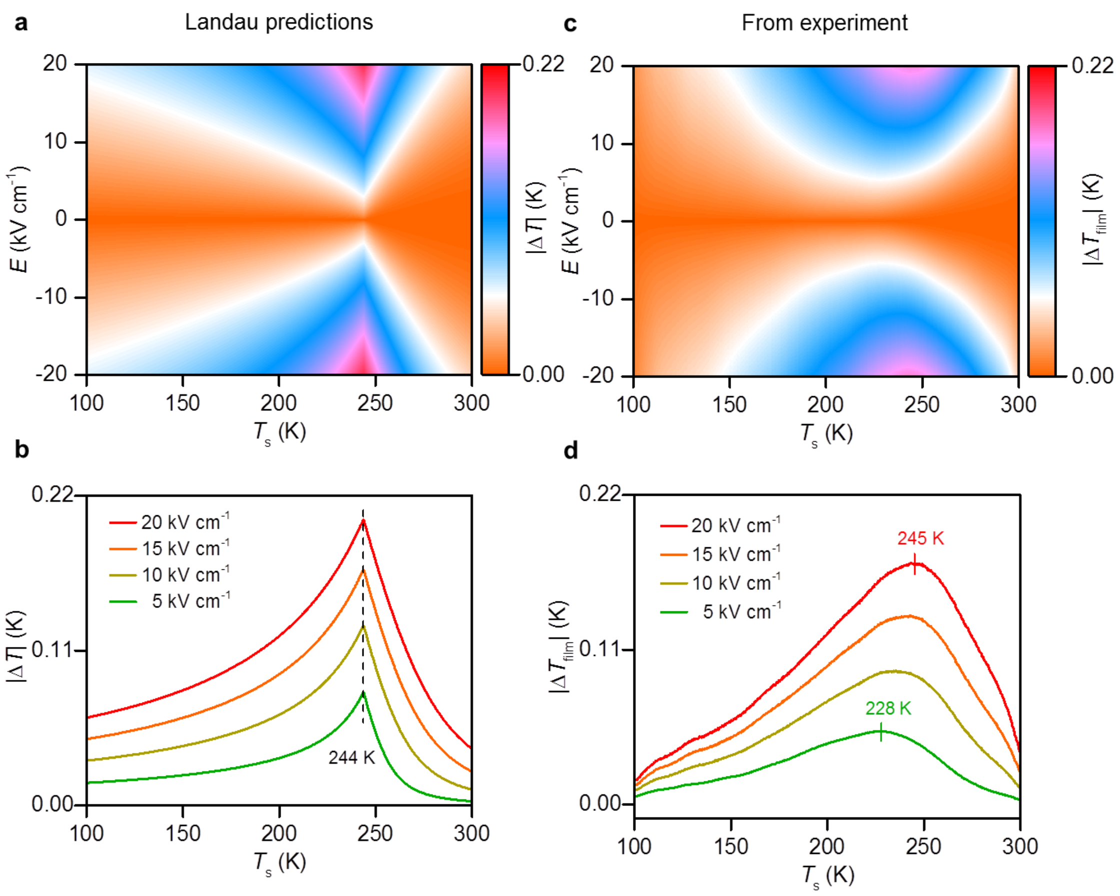

Figure S39. EC temperature change for the strained STO film. (a,b) Landau prediction of $|\Delta T(T_s,E)| \sim T_s|\Delta S(T_s,E)|/c(T_s)$ and (b) $|\Delta T(T_s)|$ transects at selected positive fields (T_s denotes starting temperature). (c,d) The corresponding information derived from experiment (after Fig. S29a,b). For (a), we used both $|\Delta S(T,E)|$ (Fig. S38a) and the volume-normalized heat capacity $c(T_s)$ (Fig. S30).

Note 20. Landau predictions for bulk STO versus results derived from experiment

Here we use the Landau model for STO//DSO (Supplementary Note 19) with zero epitaxial strain (S_m = 0) to predict the dielectric, polarization and EC properties of bulk STO above the 105 K antiferrodistortive transition[S20]. We observe a good match with the corresponding data derived from experiments (Supplementary Note 10) and presented alongside. The 'bulk' subscript is omitted when presenting Landau results in order to facilitate the use of other subscripts.

(a) Relative dielectric permittivity $\varepsilon_r(T,E)$

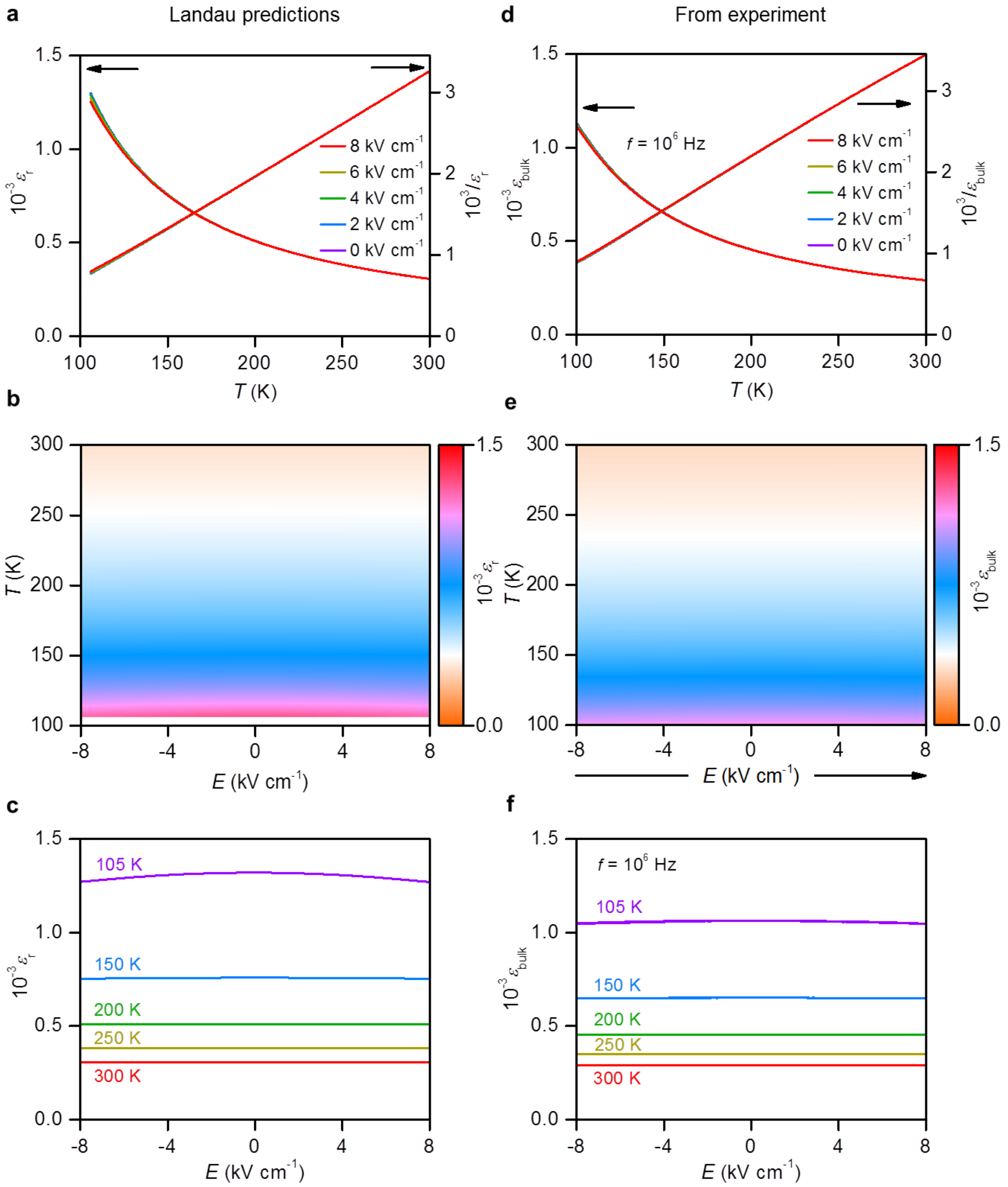

Fig S40. Dielectric response of bulk STO. (a-c) Landau predictions. (a) $\varepsilon_r(T)$ (left axis) and $1/\varepsilon_r(T)$ (right axis). (b) $\varepsilon_r(T,E)$, and (c) $\varepsilon_r(E)$ transects at five temperatures. (d-f) The corresponding information derived from experiment (Fig. S19b,c,e). The small field hysteresis observed experimentally (Fig. S19b-d) is necessarily not seen in the Landau predictions.

(b) |*P*(*T*,*E*)|

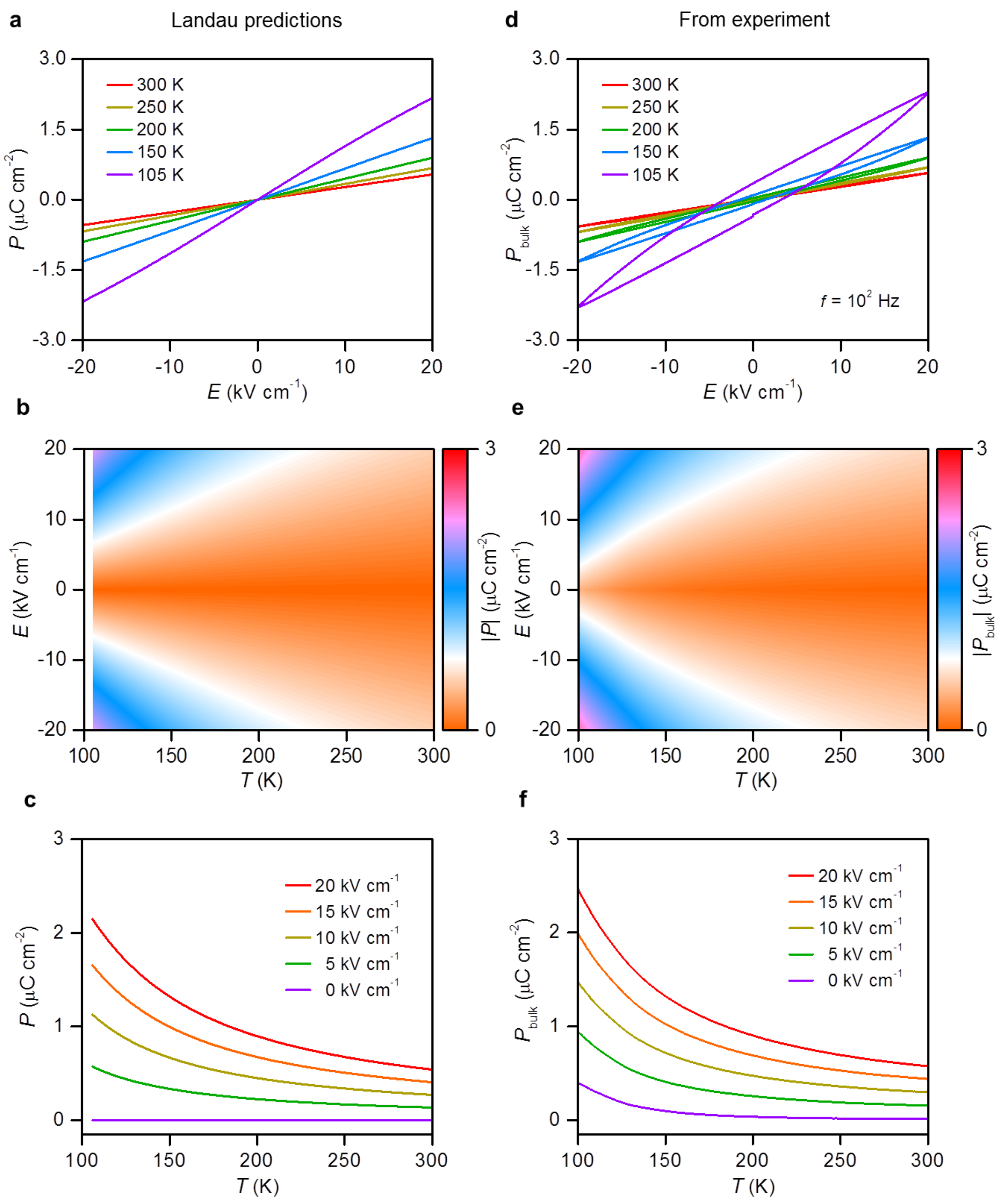

Figure S41. Ferroelectric properties of bulk STO. (a-c) Landau predictions. (a) Five bipolar $P_{\mathrm{bulk}}(E)$ plots, (b) $|P(T,E)|$, and (c) $P(T)$ transects at $E = 0$ and selected positive fields. (d-f) The corresponding information derived from experiment (Fig. S17). The field hysteresis observed experimentally at low temperatures[S12] in (d) is necessarily not seen in the Landau predictions.

(c) EC effects

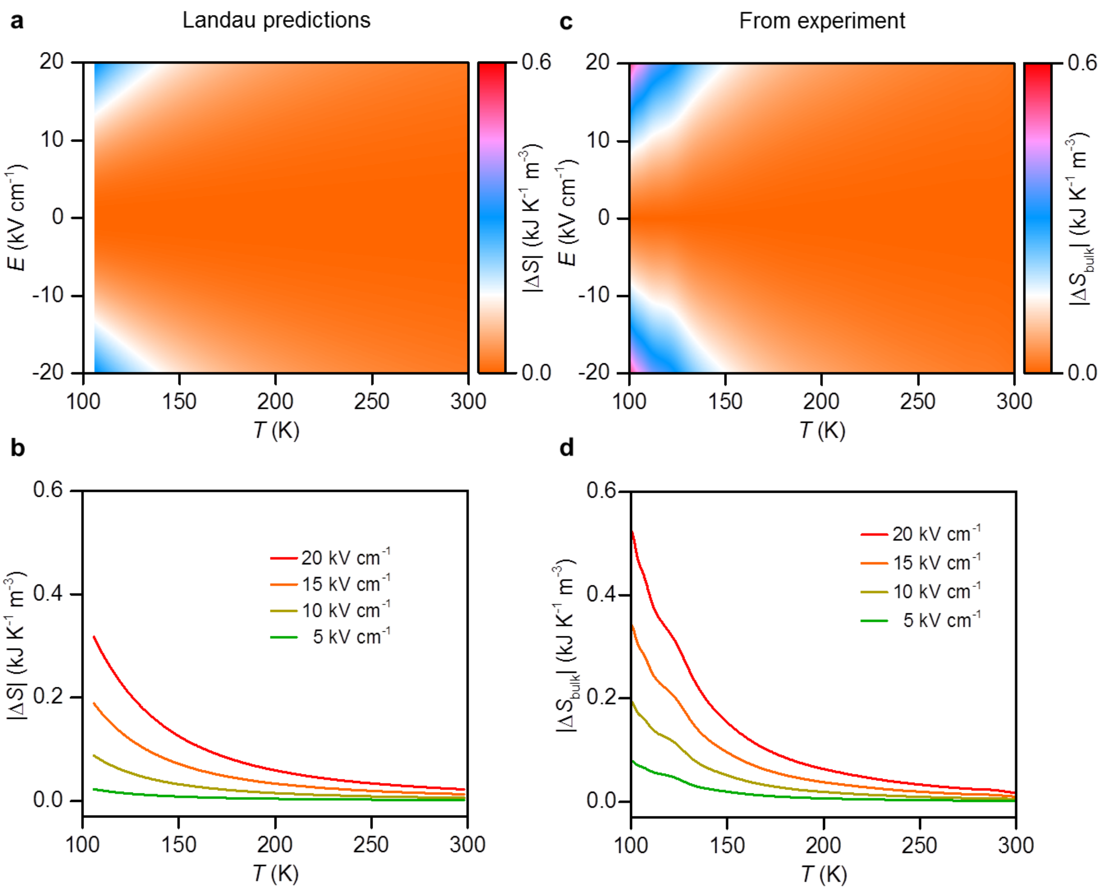

Figure S42. EC entropy change in bulk STO. (a,b) Landau prediction of (a) $|\Delta S(T,E)| = |S_L(T,0) - S_L(T,E)|$ and (b) $|\Delta S(T)|$ transects at selected positive fields. (c,d) The corresponding information derived from experiment (Fig. S18a,b).

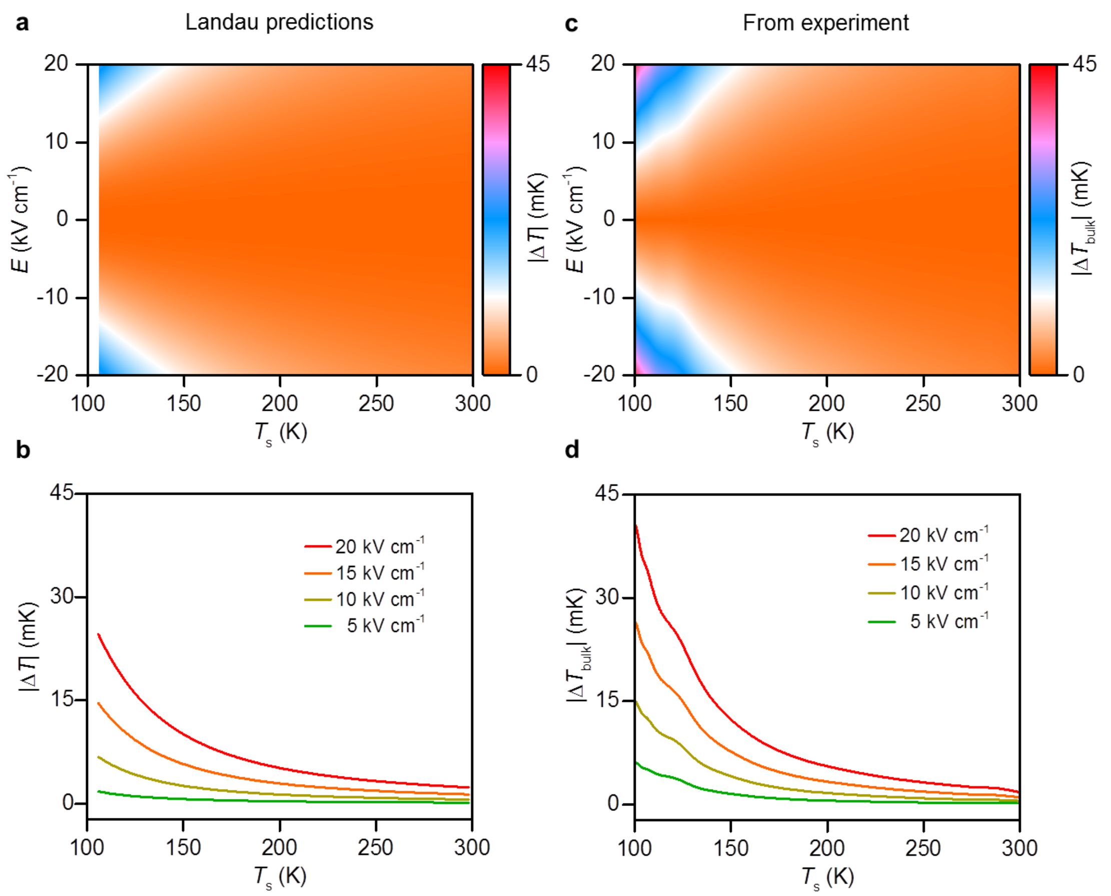

Figure S43. EC temperature change for bulk STO. (a,b) Landau prediction of $|\Delta T(T_s,E)| \sim T_s|\Delta S(T_s,E)|/c_{bulk}(T_s)$ and (b) $|\Delta T(T_s)|$ transects at selected positive fields (T_s denotes starting temperature, volume-normalized heat capacity $c_{bulk}(T_s)$ in Fig. S30). (c,d) The corresponding information derived from experiment (Fig. S18c,d).

Supplementary References

S1. G. N. Greaves, A. L. Greer, R. S. Lakes and T. Rouxel, Poisson’s ratio and modern materials, *Nature Materials* **10**, 823-837 (2011).

S2. R. P. Liferovich and R. H. Mitchell, A structural study of ternary lanthanide orthoscandate perovskites. *Journal of Solid State Chemistry* **177**, 2188-2197 (2004).

S3. L. S. Wu, S. E. Nikitin, M. Frontzek, A. I. Kolesnikov, G. Ehlers, M. D. Lumsden, K. A. Shaykhutdinov, E. J. Guo, A. T. Savici, Z. Gai, A. S. Sefat and A. Podlesnyak, Magnetic ground state of the Ising-like antiferromagnet $DyScO_3$. *Physical Review B* **96,** 144407 (2017).

S4. M. D. Biegalski, Epitaxially strained strontium titanate. *Ph.D. Thesis*, The Pennsylvania State University (2006), available at: https://www.mri.psu.edu/susan-trolier-mckinstry-group/theses-and-dissertations.

S5. S. Gevorgian, L. J. P. Linner and E. L. Kollberg, CAD models for shielded multilayered CPW. *IEEE Transactions on Microwave Theory and Techniques* **43**, 772-779 (1995).

S6. S. S. Gevorgian, T. Martinsson, P. L. J. Linner and E. L. Kollberg, CAD models for multilayered substrate interdigital capacitors. *IEEE Transactions on Microwave Theory and Techniques* **44**, 896-904 (1996).

S7. H. Wu, Z. Zhang, F. Barnes, C. M. Jackson, A. Kain and J. D. Cuchiaro, Voltage tunable capacitors using high temperature superconductors and ferroelectrics. *IEEE Transactions on Applied Superconductivity* **4**, 156-160 (1994).

S8. R. Schinzinger and P. A. A. Laura, Conformal mapping: Methods and applications. *Dover Publications*, United States (1991).

S9. C. Veyres and V. Fouad Hanna, Extension of the application of conformal mapping techniques to coplanar lines with finite dimensions. *International Journal of Electronics* **48**, 47-56 (1980).

S10. J. F. Youngblood, Low temperature dielectric measurements. *Physical Review* **98**, 1201 (1955).

S11. H. E. Weaver, Dielectric properties of single crystals of $SrTiO_3$ at low temperatures. *Journal of Physics and Chemistry of Solids* **11**, 274-277 (1959).

S12. J. Hemberger, P. Lunkenheimer, R. Viana, R. Böhmer and A. Loidl, Electric-field-dependent dielectric constant and nonlinear susceptibility in $SrTiO_3$. *Physical Review B* **52**, 13159 (1995).

S13. M. Fukunaga and Y. Noda, New technique for measuring ferroelectric and antiferroelectric hysteresis loops, *Journal of the Physical Society of Japan* **77**, 64706 (2008).

S14. A. S. Mischenko, Q. Zhang, J. F. Scott, R. W. Whatmore and N. D. Mathur, Giant electrocaloric effect in thin-film $PbZr_{0.95}Ti_{0.05}O_3$. *Science* **311**, 1270-1271 (2006).

S15. X. Moya, S. Kar-Narayan and N. D. Mathur, Caloric materials near ferroic phase transitions. *Nature Materials* **13**, 439-450 (2014).

S16. A. Durán, F. Morales, L. Fuentes and J. M. Siqueiros, Specific heat anomalies at 37, 105 and 455 K in $SrTiO_3$:Pr. *Journal of Physics: Condensed Matter* **20**, 085219 (2008).

S17. N. A. Pertsev, A. K. Tagantsev and N. Setter, Phase transitions and strain-induced ferroelectricity in $SrTiO_3$ epitaxial thin films, *Physical Review B* **61**, 825-829 (2000).

S18. N. A. Pertsev, A. K. Tagantsev and N. Setter, Phase transitions and strain-induced ferroelectricity in $SrTiO_3$ epitaxial thin films, *Physical Review B* **65**, 219901 (2002).

S19. A. P. Levanyuk, B. A. Strukov and A. Cano, Background dielectric permittivity: Material constant or fitting parameter? *Ferroelectrics* **503**, 94-103 (2016).

S20. Y. L. Li, S. Choudhury, J. H. Haeni, M. D. Biegalski, A. Vasudevarao, A. Sharan, H. Z. Ma, J. Levy, Venkatraman Gopalan, S. Trolier-McKinstry, D. G. Schlom, Q. X. Jia, and L. Q. Chen, Phase transitions and domain structures in strained pseudocubic (100) $SrTiO_3$ thin films, *Physical Review B* **73**, 184112 (2006).

S21. M. D. Biegalski, E. Vlahos, G. Sheng, Y. L. Li, M. Bernhagen, P. Reiche, R. Uecker, S. K. Streiffer, L. Q. Chen and V. Gopalan, Influence of anisotropic strain on the dielectric and ferroelectric properties of $SrTiO_3$ thin films on $DyScO_3$ substrates. *Physical Review B* **79**, 224117 (2009).

S22. M. E. Lines and A. M. Glass, Principles and applications of ferroelectrics and related materials. *Clarendon Press*, Oxford (1977).

S23. G. G. Guzmán-Verri and P. B. Littlewood, Why is the electrocaloric effect so small in ferroelectrics? *APL Materials* **4**, 64106 (2016).
